# Bioinspired leaves-on-branchlet hybrid carbon nanostructure for supercapacitors

**DOI:** 10.1038/s41467-018-03112-3

**Published:** 2018-02-23

**Authors:** Guoping Xiong, Pingge He, Zhipeng Lyu, Tengfei Chen, Boyun Huang, Lei Chen, Timothy S. Fisher

**Affiliations:** 1 Birck Nanotechnology Center, Purdue University, West Lafayette, IN 47907 USA; 2 School of Mechanical Engineering, Purdue University, West Lafayette, IN 47907 USA; 3 Department of Mechanical Engineering, University of Nevada, Reno, NV 89557 USA; 4 State Key Laboratory of Powder Metallurgy, Central South University, 410083 Changsha, China; 5 Department of Mechanical Engineering, Mississippi State University, Starkville, MS 39762 USA; 6 School of Mechanical Engineering, Yangtze University, 434023 Jingzhou, China; 7 Department of Mechanical & Aerospace Engineering and California nanoSystems Institute, University of California, Los Angeles, CA 90095 USA

## Abstract

Designing electrodes in a highly ordered structure simultaneously with appropriate orientation, outstanding mechanical robustness, and high electrical conductivity to achieve excellent electrochemical performance remains a daunting challenge. Inspired by the phenomenon in nature that leaves significantly increase exposed tree surface area to absorb carbon dioxide (like ions) from the environments (like electrolyte) for photosynthesis, we report a design of micro-conduits in a bioinspired leaves-on-branchlet structure consisting of carbon nanotube arrays serving as branchlets and graphene petals as leaves for such electrodes. The hierarchical all-carbon micro-conduit electrodes with hollow channels exhibit high areal capacitance of 2.35 F cm^−2^ (~500 F g^−1^ based on active material mass), high rate capability and outstanding cyclic stability (capacitance retention of ~95% over 10,000 cycles). Furthermore, Nernst–Planck–Poisson calculations elucidate the underlying mechanism of charge transfer and storage governed by sharp graphene petal edges, and thus provides insights into their outstanding electrochemical performance.

## Abstract (web summary)

One way to improve the performance of supercapacitors is to use hybrid carbon nanomaterials. Here the authors show a bioinspired electrode design with graphene petals and carbon nanotube arrays serving as leaves and branchlets, respectively. The structure affords excellent electrochemical characteristics.

## Introduction

Supercapacitors have elicited extensive research interest recently as power supplies to complement or even replace batteries because of their high power density, fast recharge capability, and long cycle life^1,2^. To improve energy densities for practical applications, carbon nanomaterials, particularly carbon nanotubes (CNTs)^3^, carbon nanofibers^4^, and graphene^5^, have been widely used as electrode materials because of their high surface area and electrical conductivity^5^. Highly ordered carbon nanomaterials have particularly attracted much attention because of their outstanding electrical and electrochemical properties resulting from their well-aligned structure as compared to those of randomly distributed structures^6–8^. In particular, vertical CNT arrays with a highly ordered structure are among the most promising candidates not only as active materials with improved rate capability and energy density^8,9^, but also as efficient nanotemplates for pseudocapacitive materials with enhanced cyclic stability^10^ in energy storage applications. However, common problems associated with CNT array electrodes include poor nanotube bonding to substrates, low tube-to-tube charge transfer efficiency (because of the weak Van der Waals forces between them) and easy destruction of the tube orientation, resulting in poor mechanical robustness, high internal resistance, and poor cyclic stability^11^. Although several attempts have been made to resolve the foregoing issues^12^, to date, balancing these properties (i.e., electrical conductivity, orientation, and mechanical robustness) for highly oriented carbon electrode materials remains an unmet challenge.

Hybrid carbon nanomaterials with hierarchical structures have been reported to improve the electrochemical performance of carbon-based supercapacitors^13,14^. Inspired by the common phenomenon in nature where leaves generally protect trunks/branchlets of trees, and more importantly increase exposed surface area to absorb CO_2_ (like ions) from the environments (like electrolyte) for photosynthesis, graphene petals (GPs) with large surface area, sharp edges, high electrical conductivity^15,16^, outstanding mechanical properties^17,18^, and promising electrochemical performance^19–21^ could be an ideal structure (like leaves) for integration with oriented CNT arrays (like branchlets) to address the foregoing issues. Moreover, the sharp GP edges in the hybrid structure may increase charge storage and facilitate rapid access of electrolyte ions to the electrodes, leading to improved electrochemical performance for supercapacitors^19,22,23^. To the best of our knowledge, utilizing GPs to enhance CNT arrays synergistically in terms of both mechanical and electrochemical properties for practical supercapacitor applications has not been reported in prior literature, nor have these hybrid structures been employed as nanotemplates for highly pseudocapacitive electrode materials.

**Fig. 1:**
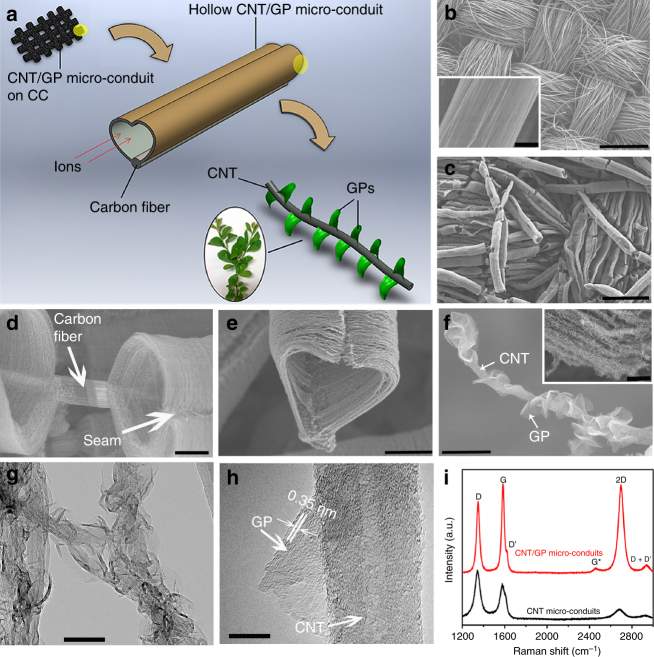
Structural characterization of CNT/GP micro-conduits. **a** Schematic illustration of CNT/GP micro-conduits in a leaves-on-branchlet nanostructure on CC substrates for high-performance supercapacitor electrodes (Note that the yellow shaded areas in the schematic indicate the selected areas to be magnified). **b** Bare CC substrate at low magnification (inset shows the surface of a single carbon fiber). **c** Uniform coverage of CNT micro-conduits on carbon fibers at low magnification. **d** A close-up of CNT micro-conduits on a carbon microfiber. **e** A CNT/GP micro-conduit in a heart shape. **f **A single CNT decorated with many GPs at high magnification (inset shows GPs on CNT micro-conduit array walls). **g** TEM image of the hierarchical structure. **h** High-resolution TEM image of a petal emerging from a nanotube. **i** Comparative Raman spectra of CNT micro-conduits and CNT/GP micro-conduits on CC substrates. Scale bars: **b** 500 μm (inset: 3 μm), **c** 300 μm, **d** 10 μm, **e** 20 μm, **f** 300 nm (inset: 2 μm), **g** 100 nm, **h** 10 nm

In the present work, we report all-carbon hybrid micro-conduit electrodes (Fig. 1a) with GPs decorating highly oriented CNT array walls in a leaves-on-branchlet nanostructure (Fig. 1a right corner)—a design inspired by tree branchlets in nature – via a two-step microwave plasma chemical vapor deposition (MPCVD) process. The micro-conduits with hollow channels were designed, to the best of our knowledge for the first time, to increase accessible electrode surface area to the electrolyte and to facilitate fast diffusion of ions during charge/discharge. GPs significantly enhance mechanical robustness of CNT micro-conduits, and thus preserve the structural orientation during operation. Such synergistically hybrid micro-conduits are demonstrated as efficient nanotemplates for highly pseudocapacitive materials and integrated in symmetric/asymmetric two-terminal devices with outstanding electrochemical performance. Furthermore, Nernst–Planck–Poisson (NPP) simulations elucidate the underlying mechanism of charge transfer and storage governed by the sharp GP edges.

## Results

### Fabrication and structural characterization of CNT/GP micro-conduits

All-carbon CNT/GP micro-conduits were fabricated by a two-step process within an MPCVD chamber described in Methods section. Scanning electron microscopy (SEM) and transmission electron microscopy (TEM) images in Fig. 1 reveal the typical structure and morphology of CNT/GP micro-conduits in the leaves-on-branchlet nanostructure. Before CNT micro-conduit growth, the surface of bare carbon fibers, with an average diameter of ~9 µm, is relatively smooth with some visible trenches as shown in Fig. 1b (inset). After growth for 10 min, unlike the conventional phenomenon in which CNTs grow homogeneously and vertically on carbon fiber surface in the absence of plasma^24^, CNT arrays form in the shape of micro-conduits (outer diameter of 30–40 µm, Fig. 1c, d) from the pre-deposited tri-layer catalysts on the surface of carbon microfibers. Seams are observed on the top of all micro-conduits (Fig. 1c and related images in Supplementary Fig. 1), and these CNT micro-conduits form a heart shape (Fig. 1d, e and Supplementary Fig. 1). The diameters of the nanotubes in the array range from 20 to 30 nm (Supplementary Fig. 1). Such micro-conduit structures with hollow channels increase accessible electrode surface area to the electrolyte and facilitate fast diffusion of ions during charge/discharge, enabling high rate capability and power delivery.

To understand why CNT arrays form in the shape of micro-conduits, we systematically studied the growth phenomenon by adjusting CNT growth durations, keeping all other parameters the same. CNT morphologies corresponding to different growth durations of 1, 2, 3, 5, 7, and 10 min are shown in Supplementary Fig. 2. The SEM images clearly indicate that CNTs grow uniformly from catalysts deposited on carbon fibers at an initial stage, and then gradually form two thin open walls with the influence of plasma. As growth time extends, the height of CNT walls increases, and eventually the tips of the two walls touch each other, forming a hollow conduit with a microscale size. In addition, to further identify the effect of plasma on the formation of CNT micro-conduits during the growth, control experiments were conducted to assess CNT growth without the influence of plasma (Supplementary Methods). SEM images in Supplementary Fig. 3 display the morphologies of CNTs prepared under such conditions, and the results indicate that the plasma crucially affects the formation of such micro-conduit structures possibly due to the internal electric fields in the near-wall region affected by the growing walls. Quantitative analysis of the plasma influence will be carried out in the future simulation work. Moreover, the catalyst and substrate effects on the formation of CNT micro-conduit provided in Supplementary Figs. 4 and 5, respectively, indicate that the growth of CNT micro-conduit is highly dependent on triple-layer metal catalyst and the geometric size of the substrate. Due to the cylindrical structure of carbon microfibers, the varying thickness of catalyst layers on these microfibers will lead to different sizes of catalyst nanoparticles, resulting in different catalytic activity, and thus different CNT growth rates and morphologies.

After GP growth in the same chamber by MPCVD for 18 min (detailed procedure is shown in Methods section), uniform GPs decorate CNTs along the axial direction on the micro-conduit surfaces. The GP growth mechanism has been demonstrated in our previous work^16^. Figure 1e displays a typical CNT/GP micro-conduit in a heart shape at relatively low magnification (more images are shown in Supplementary Fig. 6), in which apparent seams on the top of the micro-conduits are observed. A close-up SEM image of the hybrid micro-conduit shows that the GPs are decorating CNTs homogenously in a bioinspired nanostructure consisting of leaves on a branchlet, in which a CNT serves as a branchlet and GPs as leaves (Fig. 1f). GPs exhibit sharp edges, which can facilitate rapid access of electrolyte ions to the surfaces of electrodes with short ion diffusion length, leading to superior electrochemical performance^25^.

A TEM image in Fig. 1g clearly displays the unique hierarchical structure with uniform distribution of GPs on CNTs. A typical CNT exhibits a distinct hollow core and an outer diameter of ~30 nm with fringes on each side of the tube (Fig. 1h). GPs grown on CNTs display a typical size of 100 nm, smaller than those grown on flat substrates in prior work^16,26^. Moreover, the high-resolution TEM image in Fig. 1h indicates the graphitic nature of GPs and their thickness of several nanometers. Comparative Raman spectra of CNT and CNT/GP micro-conduits on CC substrates are shown in Fig. 1i, in which both spectra contain prominent bands near 1350, 1580, 1620, and 2700 cm^−1^ corresponding to the D, G, D′, and 2D (also called G′) bands, respectively, and the spectrum of CNT/GP micro-conduits exhibits additional prominent bands located near 2450 and 2940 cm^–1^ corresponding to the G* and D + D′ bands, respectively^27,28^. The peak intensity ratio of the D and G peaks (*I*_D_/*I*_G_) and that of the 2D and G peaks (*I*_2D_/*I*_G_) are ~1.35 and 0.35 for CNT micro-conduits, respectively. The *I*_D_/*I*_G_ of micro-conduits after GP decoration reduces to ~0.87, indicating fewer defects of the hybrid CNT/GP nanostructure than bare CNTs. The *I*_2D_/*I*_G_ of CNT/GP micro-conduits is calculated to be 0.99, indicating that the GPs are few-layer graphene. These Raman analyses agree well with the TEM results in Fig. 1g, h. With a GP growth time of 18 min, the mass ratio of GPs to CNTs is ~4:1, with a total areal mass density of 4.75 mg cm^−2^. GP loading can be easily controlled by the growth duration, and longer growth durations give denser GPs on CNT micro-conduits. Supplementary Fig. 7 shows the CNT micro-conduits decorated by GPs with a growth of 35 min, in which a large amount of GPs are tightly configured on CNT walls, forming a highly interconnected network.

### Electrochemical characterization of CNT/GP mechanically robust micro-conduits

**Fig. 2:**
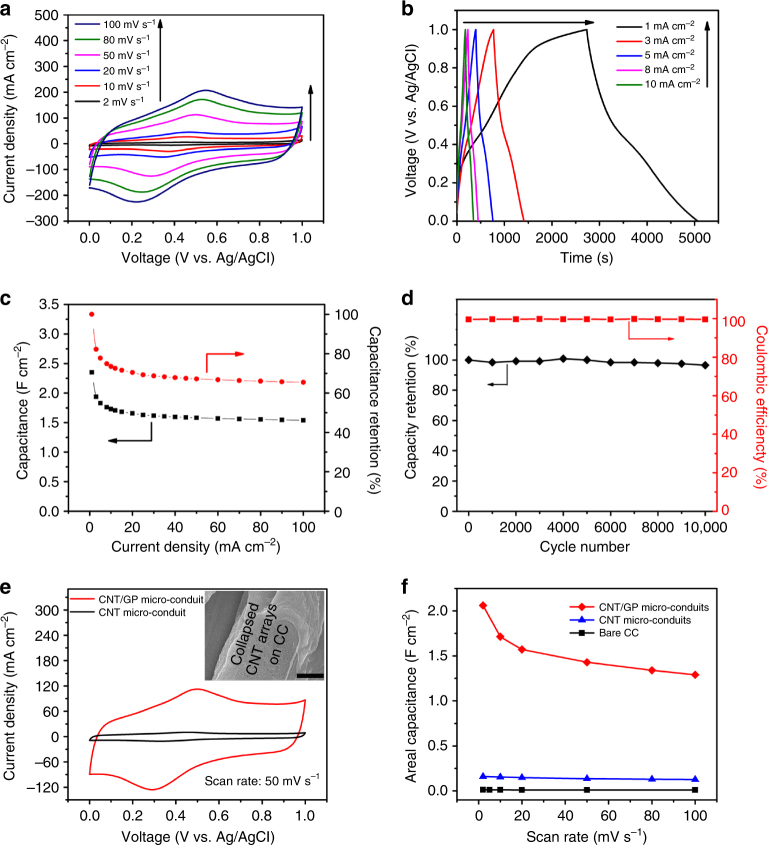
Electrochemical characterization of CNT/GP micro-conduits. **a** CV curves of a typical CNT/GP micro-conduit electrode (GP growth time of 18 min) in a three-electrode configuration with 1 M H_2_SO_4_ aqueous solution at scan rates from 2 to 100 mV s^−1^. **b** Galvanostatic charge/discharge profiles of the CNT/GP micro-conduit electrode at current densities from 1 to 10 mA cm^−2^. **c** Specific capacitances and capacitance retention as functions of current densities of the CNT/GP micro-conduit electrode. **d** Charge/discharge cyclic stability at a current density of 60 mA cm^−2^ and coulombic efficiencies during the test. **e** Comparative CV curves of typical CNT micro-conduit and CNT/GP micro-conduit electrodes at a scan rate of 50 mV s^–1^. The inset (Scale bar: 5 μm) shows that the bare CNT micro-conduit structure collapses and forms a matt-like structure on the CC micro-fiber. **f** Comparison of areal specific capacitances of bare CC, CNT micro-conduit, and CNT/GP micro-conduit electrodes at different scan rates from 2 to 100 mV s^−1^

Prior to the electrochemical characterization of CNT/GP micro-conduit electrodes in 1 M H_2_SO_4_ aqueous electrolyte, electrochemical activation to make them hydrophilic is necessary, because carbon materials are generally hydrophobic by nature^2^. Figure 2a displays cyclic voltammetry (CV) curves of the CNT/GP micro-conduit electrode at scan rates of 2, 10, 20, 50, 80, and 100 mV s^–1^ in a voltage range between 0 to 1 V. After the electrochemical oxidization process, the CNT/GP micro-conduit surface is functionalized with oxygen-containing functional groups, significantly improving the wettability of the hybrid all-carbon nanostructure, and thus increasing charge storage^21^. CV curves at all scan rates exhibit two mild redox peaks, which can be attributed to the oxidation/reduction of the oxygen-containing functional groups in the acidic electrolyte^29^.

Figure 2b displays galvanostatic charge/discharge profiles of the CNT/GP micro-conduit electrode at different current densities ranging from 1 to 10 mA cm^−2^ (profiles at higher current densities from 40 to 100 mA cm^−2^ are available in Supplementary Fig. 8). Mild potential plateaus in the charge/discharge curves are consistent with the potentials of redox peaks observed in the CV curves. Based on the charge/discharge curves, the areal capacitance of the CNT/GP micro-conduit electrode calculated by the method described in Supplementary Methods reaches 2.35 F cm^−2^ at a current density of 1 mA cm^−2^, 10–1000 times higher than those of the state-of-the-art carbon nanostructure-based electrodes that fall in the range of 0.001–0.1 F cm^−2^ ^30–32^. Meanwhile, the specific capacitance of the CNT/GP micro-conduit electrode based on the total mass of CNTs and GPs is calculated to be ~500 F g^−1^ at a current density of 1 mA cm^−2^, which is substantially higher than the reported values (in the range of 100–385 F g^−1^) for contemporary all-carbon electrode materials (Supplementary Table 1).

Figure 2c shows the areal capacitance and capacitance retention of the CNT/GP micro-conduit electrode plotted as functions of discharge current density. As the current density increases from 1 to 100 mA cm^−2^, areal capacitances of the micro-conduit electrode display a gradual attenuation from 2.35 F cm^−2^ at a current density of 1 mA cm^−2^ to 1.54 F cm^−2^ at a current density of 100 mA cm^−2^, with a capacitance retention of 66%. Such a high rate capability is comparable to or higher than those reported for many carbon-based electrodes^33,34^. The areal capacitances of CNT/GP micro-conduit electrodes with different GP growth durations (5, 10, 18, 25, and 35 min) have been plotted as a function of scan rates in Supplementary Fig. 9. The results reveal a relationship between the edge density of GP and capacitance of CNT/GP micro-conduit electrodes, and indicate that an optimal GP growth time exists for superior electrochemical performance because of a balance between surface area and ion transfer kinetics. Comparative Nyquist plots for bare CNT and CNT/GP micro-conduit electrodes recorded from 0.1 Hz to 1 MHz with an alternating current (AC) perturbation amplitude of 5 mV are provided in Supplementary Fig. 10, from which the series resistance *R*_e_ (derived from electrode, electrolyte, and the interface between electrode and electrolyte) of CNT and CNT/GP micro-conduit electrodes is measured to be 1.85 and 2.8 Ω, respectively. Moreover, negligible semicircles in the high frequency region from impedance spectra of both electrodes indicate a low charge transfer resistance *R*_ct_ of CNT and CNT/GP micro-conduit electrodes. The cyclic stability of CNT/GP micro-conduit electrodes was evaluated at a current density of 60 mA cm^−2^ in the potential range between 0 and 1 V over 10,000 cycles, as shown in Fig. 2d. The CNT/GP micro-conduit electrode exhibits ~95% capacitance retention over 10,000 charge/discharge cycles and high coulombic efficiencies close to 100%, indicating excellent long-term cyclic stability and high charge-storage efficiency with negligible side reactions.

To demonstrate the synergistic effects of GPs on the electrochemical performance of hierarchical CNT/GP micro-conduits, bare CNT micro-conduit electrodes without GPs were fabricated and characterized under the same conditions for comparison. Figure 2e displays the comparative CV curves of CNT micro-conduit and CNT/GP micro-conduit electrodes at a scan rate of 50 mV s^–1^ (CV curves of bare CNT micro-conduit electrodes from 2 to 100 mV s^–1^ are provided in Supplementary Fig. 11a). Comparative areal capacitances under different scan rates for bare CC, CNT micro-conduit, and CNT/GP micro-conduit electrodes are shown in Fig. 2f. At a scan rate of 2 mV s^–1^, the areal capacitance of the CNT/GP micro-conduit electrode is more than an order of magnitude higher than that of the CNT micro-conduit electrode. Galvanostatic charge/discharge profiles of the CNT electrode at different current densities ranging from 1 to 10 mA cm^−2^ are provided in Supplementary Fig. 11b. Based on these charge/discharge curves, the areal capacitance of the CNT electrode is calculated to be 0.167 F cm^−2^ at a current density of 1 mA cm^−2^, an order of magnitude lower than that of the CNT/GP micro-conduit electrode and in agreement with the results calculated from CV curves.

The substantially enhanced electrochemical performance of CNT/GP micro-conduit electrodes is attributed to the synergistic effects of the two carbon nanostructures. First, GPs enhance the mechanical robustness. As demonstrated in Fig. 2e inset and Supplementary Fig. 12, bare CNT micro-conduits suffer from severe structural damage and deformation during normal operation in electrolyte, indicating their poor mechanical integrity and stability (Supplementary Fig. 12a and 12b). Once wetted by the aqueous electrolyte, CNTs densify locally and form collapsed layers on the carbon fiber surface (Fig. 2e inset and Supplementary Fig. 12d). In sharp contrast, after GP decoration of CNT micro-conduits, the leaves-on-branchlet nanostructure remains intact during electrochemical measurements and even after being immersed in concentrated strong acids consisting of H_2_SO_4_ and HNO_3_ (v/v = 3:1) at elevated temperature ( > 40°C) overnight as a test of mechanical integrity under extreme conditions (Supplementary Fig. 13). Moreover, the CNT/GP micro-conduit electrodes were ultrasonically treated in a water bath for 30 min and the results are provided in Supplementary Fig. 14, further revealing the high mechanical robustness of CNT/GP micro-conduit structures. These results demonstrate that GPs vividly enhance the mechanical robustness of CNT micro-conduits, and the hybrid micro-conduits possess outstanding electrochemical stability against potential electrolytes, providing an essential prerequisite as high-performance all-carbon active materials or an efficient platform for highly pseudocapacitive materials. Second, GPs enhance surface area and electrical conductivity. Highly oriented CNT arrays possess good electrical properties along the axial direction of nanotubes; however, because of weak Van der Waals forces between nanotubes in the array and sparse packing (i.e., gaps among nanotubes), charge transfer between adjacent nanotubes may not be efficient. GP decoration on CNT micro-conduits enhances the surface area and electrical behavior by forming a leaves-on-branchlet structure and tightly interconnected network, leading to superior electrochemical performance. The nitrogen (N_2_) adsorption–desorption isotherms of CNT/GP micro-conduit electrodes with different GP growth times are provided in Supplementary Fig. 15, and the measured BET surface area (with GP growth time of 18 min) is 30.6 m^2^ g^−1^, which is nearly 2 times greater than that of the bare CNT micro-conduit structure (15.5 m^2^ g^−1^) (Supplementary Fig. 15), 100 times greater than that of CC/GPs (0.32 m^2^ g^−1^) and 300 times greater than that of pure CC (0.11 m^2^ g^−1^), confirming the large specific surface area of CNT/GP micro-conduit electrodes. Figure 1g and Supplementary Figs. 6 and 7 show that the tightly interconnected 3D network provides a crucial prerequisite for the superior electrochemical performance of CNT/GP micro-conduit electrodes.

### Efficient nanotemplates for highly pseudocapacitive materials

The CNT/GP micro-conduit structure not only provides high performance as an active electrode material, but it also can serve as an efficient nanotemplate for pseudocapacitive materials. In this section, transition metal hydroxide (Ni-Co hydroxide) and conducting polymer (polyaniline, PANI) were chosen separately as typical pseudocapacitive materials to demonstrate the potential of CNT/GP micro-conduits for pseudocapacitive electrode applications. Electrodeposition durations of Ni-Co hydroxide and PANI on nanotemplates followed the optimized conditions described in prior work (Supplementary Methods)^17,22^. The morphologies of Ni-Co hydroxide and PANI electrodeposited on nanotemplates are provided in Supplementary Fig. 16 and Fig. 17, respectively.

**Fig. 3:**
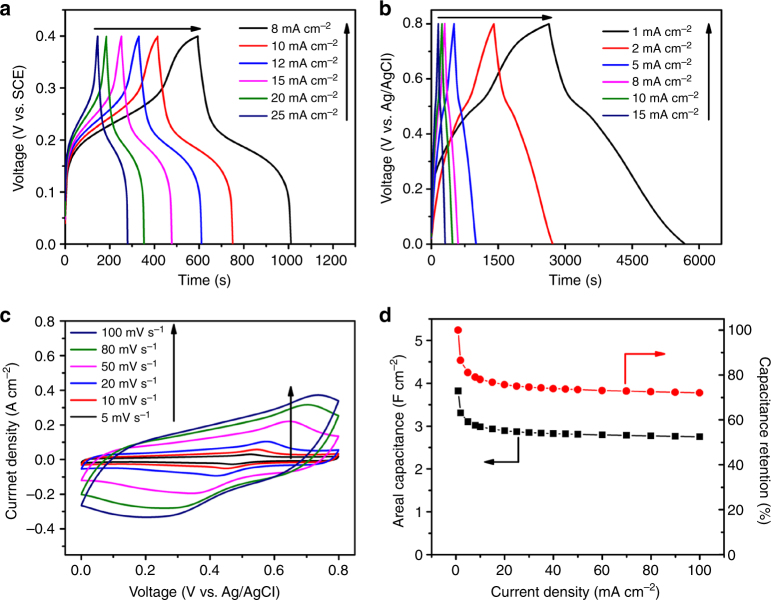
Electrochemical performance of pseudocapacitive CNT/GP micro-conduit electrodes. **a** Galvanostatic charge/discharge profiles of a CNT/GP/Ni-Co hydroxide micro-conduit electrode in 2 M KOH aqueous electrolyte at different current densities in the voltage range between 0 and 0.4 V vs. SCE. **b** Galvanostatic charge/discharge profiles of a CNT/GP/PANI micro-conduit electrode in 1 M H_2_SO_4_ aqueous solution at different current densities in the voltage range between 0 and 0.8 V vs. Ag/AgCl. **c** CV curves of the CNT/GP/PANI micro-conduit electrode at scan rates from 5 to 100 mV s ^– 1^ with a voltage window of 0.8 V. **d** Areal capacitance and capacitance retention as a function of current density of the CNT/GP/PANI micro-conduit electrode calculated from charge/discharge curves

Figure 3a displays galvanostatic charge/discharge profiles of a CNT/GP/Ni-Co hydroxide micro-conduit electrode (with a hydroxide electrodeposition time of 3 min) in 2 M KOH aqueous electrolyte at low current densities in the voltage range between 0 and 0.4 V vs. SCE (curves at higher current densities up to 100 mA cm^−2^ are shown in Supplementary Fig. 18a). At a current density of 8 mA cm^−2^, the areal capacitance of the hybrid electrode reaches 8.7 F cm^−2^, which is ~5 times higher than that (1.8 F cm^−2^) of the CNT/GP micro-conduit electrode, and even higher than that (~7.5 F cm^−2^) of a previously reported GP foam/Ni-Co hydroxide electrode (electrodeposition time of 3 min)^17^. At a current density of 100 mA cm^−2^, the areal capacitance exhibits ~6.1 F cm^−2^, which is ~71% compared to that at 8 mA cm^−2^, indicating high rate capability (Supplementary Fig. 18b). CV curves for scan rates from 2 to 30 mV s^−1^ for the CNT/GP/Ni-Co hydroxide micro-conduit electrode are provided in Supplementary Fig. 18c. Redox peaks are clearly observed, indicating the existence of faradaic reactions between Ni-Co hydroxide and electrolytes. A low series resistance in the CNT/GP/Ni-Co hydroxide micro-conduit electrode is demonstrated in the results of Supplementary Fig. 18d.

Figure 3b shows charge/discharge curves of a CNT/GP/PANI micro-conduit electrode (with a PANI electropolymerization time of 5 min) in 1 M H_2_SO_4_ aqueous electrolyte at low current densities in the voltage range between 0 and 0.8 V vs. Ag/AgCl. Charge/discharge curves at higher current densities up to 100 mA cm^−2^ are shown in Supplementary Fig. 19a. CV curves of the CNT/GP/PANI micro-conduit electrode in 1 M H_2_SO_4_ aqueous solution at scan rates from 5 to 100 mV s^–1^ are displayed in Fig. 3c. Figure 3d shows areal capacitances of the hybrid electrode calculated from charge/discharge curves in Fig. 3b and Supplementary Fig. 19a as a function of current densities. Figure 3d reveals that the areal capacitance reaches as high as 3.82 F cm^−2^ at a current density of 1 mA cm^−2^, which is ~1.6 times higher than that (2.35 F cm^−2^) of CNT/GP micro-conduit electrodes, and even higher than that (<2 F cm^−2^) of GP/PANI electrodes at a low current density^22^. At a high current density of 100 mA cm^−2^, the areal capacitance of the CNT/GP/PANI electrode still remains ~2.75 F cm^−2^, exhibiting high capacitance retention of 72% compared to that at 1 mA cm^-2^, indicating a rate capability higher than those of PANI-based electrodes in prior work^35,36^. The Nyquist plot for CNT/GP/PANI micro-conduit electrodes recorded from 0.1 Hz to 1 MHz with an AC perturbation amplitude of 5 mV is provided in Supplementary Fig. 19b. The Nyquist plots for the two pseudocapacitive electrodes indicate that internal resistances in both systems with CNT/GP micro-conduits as nanotemplates are quite low, leading to high capacitance, high energy and power density, and high rate capability.

### Electrochemical performance of CNT/GP micro-conduit-based supercapacitor devices

**Fig. 4:**
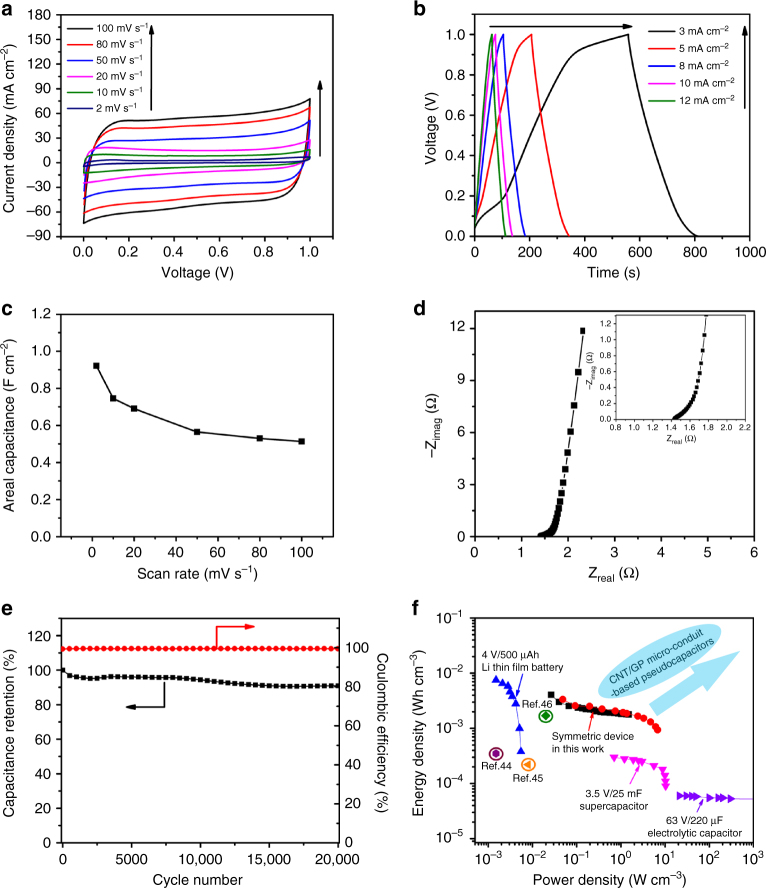
Electrochemical performance of two-terminal symmetric supercapacitor devices. **a** CV curves of the symmetric supercapacitor device at scan rates from 2 to 100 mV s^−1^ with a voltage range between 0 and 1 V in 1 M H_2_SO_4_. **b** Galvanostatic charge/discharge curves of the present device at low current densities from 3 to 12 mA cm^−2^ in the voltage range between 0 and 1 V. **c** Capacitance of the device as a function of scan rate. **d** Nyquist plot for the present symmetric device recorded from 0.1 Hz to 1 MHz with an AC perturbation amplitude of 5 mV. **e** Cyclic stability and coulombic efficiencies of the present supercapacitor. **f** Comparative Ragone plot of a typical electrolytic capacitor, a Li-ion thin-film battery, a commercial supercapacitor (from ref. ^39^), contemporary energy devices [44–46], and the present symmetric device

To further demonstrate the functional properties of such micro-conduit electrodes for practical applications, two-terminal symmetric all-carbon supercapacitors were assembled with two identical CNT/GP micro-conduit electrodes (Supplementary Methods) and then electrochemically characterized. These supercapacitor devices were tested in 1 M H_2_SO_4_ aqueous electrolyte, and the characteristic electrochemical performance of a typical symmetric device is provided in Fig. 4. CV curves recorded at scan rates ranging from 2 to 100 mV s^–1^ (Fig. 4a) display a nearly ideal rectangular shape, and these curves maintain a good shape even at scan rates up to 1000 mV s^–1^ (Supplementary Fig. 20a), indicating fast ion diffusion, high rate capability, and low internal resistance. Figure 4b shows galvanostatic charge/discharge curves of the symmetric device at different current densities ranging from 3 to 12 mA cm^−2^ (curves at higher current densities from 30 to 100 mA cm^−2^ are shown in Supplementary Fig. 20b). Areal capacitances of the device are plotted as a function of corresponding scan rates in Fig. 4c. At a scan rate of 2 mV s^–1^, the areal capacitance of the device reaches 0.92 F cm^−2^, which is superior to those of state-of-the-art carbon-based supercapacitors^37–40^. As shown in Fig. 4c, with increasing scan rates the areal capacitances of the device exhibit a gradual decrease from 0.92 F cm^−2^ at 2 mV s^–1^ to 0. 52 F cm^−2^ at 100 mV s^–1^, with a capacitance retention close to 60%.

Equivalent series resistance is a crucial factor affecting the electrochemical performance of supercapacitor devices. A Nyquist plot for the present symmetric device is recorded from 0.1 Hz to 1 MHz with an AC perturbation amplitude of 5 mV (Fig. 4d). Calculated from the impedance spectrum in Fig. 4d, the series resistance *R*_e_ is 1.43 Ω. Moreover, the negligible semicircle in the high-frequency region indicates a low charge transfer resistance *R*_ct_ (Fig. 4d inset). The low *R*_e_ and *R*_ct_ values indicate that the symmetric supercapacitor possesses a low internal resistance, deriving from the low electrical resistivity of the electrode material, high diffusivity of electrolyte ions, and low charge transfer resistance at interfaces between the electrodes and electrolyte. Cyclic stability of the as-prepared device was tested over 20,000 galvanostatic charge/discharge cycles at a current density of 80 mA cm^−2^ in the voltage range from 0 to 1 V. As shown in Fig. 4e, the present symmetric device exhibits a gradual decrease in capacitance over 20,000 cycles, with a capacitance retention of ~91% compared to the first cycle that is substantially higher than those of the state-of-the-art carbon material-based supercapacitor devices reported in prior work^41–43^. Moreover, the device exhibits high coulombic efficiencies (>99%, calculated by the method described in Supplementary Methods) over the 20,000 cycles, indicating high charge transfer efficiencies over long-term cycling.

A comparative Ragone plot (normalized by the volume of the entire fabricated device) is given in Fig. 4f to compare the performance of the present all-carbon device with various contemporary energy storage devices^44–46^, typical electrolytic capacitors, commercial supercapacitors, and lithium thin-film batteries cited from ref. ^39^. Energy and power densities calculated from both CV and galvanostatic charge/discharge measurement techniques are provided here for comparison and should be complementary to reflect the overall performance of the device. Figure 4f shows that energy and power densities calculated from the two independent measuring techniques agree well with each other. The device exhibits an average energy density up to ~4 mWh cm^−3^, which is more than 10 times higher than a commercial 3.5 V/25-mF supercapacitor and comparable to the upper range of a lithium thin-film battery, and delivers a power density up to ~6.5 mW cm^−3^, which is over two orders of magnitude higher than typical lithium thin-film batteries. Meanwhile, a comparative Ragone plot (per total mass of active materials, CNTs and GPs) is also provided and compared with state-of-the-art reports on carbon-based supercapacitor devices in Supplementary Fig. 21, which indicates that the energy density of the present symmetric device reaches ~16.4 Wh kg^−1^, and the device also delivers a power density up to ~27 kW kg^−1^. These performance metrics are significantly higher than those of contemporary carbon-based supercapacitors^47–49^, indicating the outstanding overall performance of the present symmetric all-carbon supercapacitor.

### Simulation of charge transfer and storage behavior

**Fig. 5:**
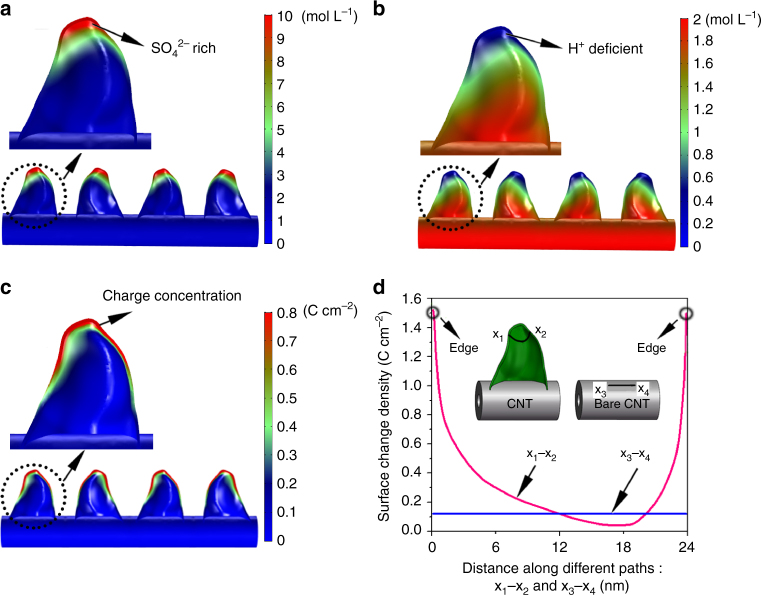
Simulations of CNT/GP micro-conduit electrodes in 1 M H_2_SO_4_ at a voltage of 1 V. **a** Contour plot of counter-ion (SO_4_^2–^) distribution (initial density: 1 M). **b** Contour plot of co-ion (H^+^) distribution (initial density: 2 M). **c** Contour plot of surface charge density distribution . **d** Comparisonof surface charge density between CNT/GP and bare CNT cases. The magenta line corresponds to the path *x*_1_–*x*_2_ passing through both edge and basal regions of a typical petal in the CNT/GP case, and blue line corresponds to the path *x*_3_–*x*_4_ on the top outer surface of a bare CNT along its axial direction

To understand the experimentally measured electrochemical performance of the CNT/GP micro-conduit structure, we performed atomically informed NPP calculations of the Gouy–Chapman model to elucidate the underlying mechanisms of charge transfer and storage (see Methods section). Figure 5 shows the calculated distributions of charge, counter-ions (SO_4_^2–^), and co-ions (H^+^) at equilibrium in supercapacitor devices consisting of symmetric CNT/GP micro-conduit electrodes in a 1 M H_2_SO_4_ aqueous electrolyte solution subjected to a voltage of 1 V. Due to device symmetry, only the positive electrode was selected for detailed calculations and analyses. As indicated in the Supplementary Eq. (7), ion transport behavior is governed by both diffusion (first term) and electrostatic (second term) forces; in particular, the latter’s contribution increases as charge accumulates. Therefore, the positive electrode immersed in electrolyte solution initially attracts counter-ions (SO_4_^2–^), whereas repelling co-ions (H^+^) primarily due to electrostatic forces when ion densities are relatively uniform, thus rendering the diffusive driving force small (Supplementary Fig. 22). Because of the unique sharp edges of the leaves-on-branchlet nanostructure, counter-ions (SO_4_^2–^) do not distribute uniformly on the electrode surface, but tend to accumulate near the sharp edges of GPs, as shown in Fig. 5a. Meanwhile, a deficiency of co-ions (H^+^) is also observed at the edge region of GPs as shown in Fig. 5b. Consequently, net charge, equal to the density difference of counter-ions and co-ions (i.e., $C_{{\mathrm{SO}}_4} - C_{\mathrm{H}}$), aggregates at the edge regions of GPs as shown in Fig. 5c. The net charge aggregation in turn results in a larger electrostatic driving force near the edges, and thus leads to accumulation of SO_4_^2–^, as well as deficiency of H^+^, providing a greater net charge concentration near the edges until the diffusion and electrostatic forces reach a balance. Such a strongly non-uniform distribution of charge becomes increasingly prevalent when approaching the tip of petals with a large curvature.

To quantify the edge effect of GPs, the counter-ion concentration, co-ion concentration, and surface charge density $\rho ^s$ were calculated along one typical path (*x*_1_–*x*_2_) passing through both the edge and basal regions of petals as an example, and the results are plotted (magenta line) in Supplementary Fig. 23a, Supplementary Fig. 23b, and Fig. 5d, respectively. The calculated counter-ion concentration at the edge region is 3.5 times higher than that at the basal region, while the co-ion concentration at the sharp edges is 3 times lower than that at the basal region along the chosen path. Moreover, comparison of the surface charge density at the edge region $\rho _{\mathrm{edge}}^s$ and at the basal region $\rho _{\mathrm{basal}}^s$ yields a charge concentration factor (CCF, defined as CCF = $\rho _{\mathrm{edge}}^s/\rho _{\mathrm{basal}}^s$) of ~8 along the chosen path (Fig. 5d). The obtained charge concentration at the GP edges is consistent with recent density functional theory (DFT) and molecular dynamics (MD) results reported by Bo et al.^23^ in which GP electrodes were simplified as parallel vertically oriented single-layer graphene arrays. A significant charge concentration at the GP edge region has been well recognized to enhance the kinetics of electrolyte ion penetration into GP interiors, thus resulting in an improved accessibility of electrolyte^23^.

To further explore the unique edge effect of GPs, a bare CNT electrode without petals was modeled for comparison under the same boundary and initial conditions, and the results are shown in Fig. 5d and Supplementary Fig. 24. As presented in Fig. 5d (blue line), the surface charge density is almost uniform along a typical path *x*_3_–*x*_4_ in Fig. 5d on the outer surface of the CNT along the axial direction. More importantly, the surface charge density of the CNT/GP micro-conduit electrode, whether at the edge or throughout the basal region, exhibits a substantial enhancement compared to that of the bare CNT electrode as shown in Supplementary Fig. 24. The overall effect of such a charge density enhancement can be characterized by areal capacitance (see Supplementary Methods) for the two cases. The areal capacitance of the CNT/GP electrode was predicted to be 1.78 F cm^−2^, and the corresponding value was 0.26 F cm^−2^ for the bare CNT case at equilibrium under voltage control mode subjected to a voltage of 1 V, thus presenting more than six-fold improvement in areal capacitance owing to the existence of GPs, qualitatively consistent with the foregoing experimental results in which the petals produce substantial enhancement in areal capacitance.

## Discussion

The superior electrochemical performance of CNT/GP micro-conduit electrodes to that of bare CNT micro-conduit electrodes (an order-of-magnitude improvement in capacitance, Fig. 2f) can be attributed to the unique leaves-on-branchlet nanostructure and 3D interconnected all-carbon network, providing substantial advantages in electrochemical, mechanical, and electrical properties, which are explained in detail as follows: (1) The shape of CNT/GP micro-conduits is maintained compared to the bare CNT case in aqueous electrolyte and even in strong acid H_2_SO_4_ and HNO_3_ (v/v = 3:1) at elevated temperature (Supplementary Fig. 13). (2) GP decoration on CNTs enhances electrical contact between CNT tubes by forming interconnected 3D networks, thus increasing electron transport efficiency between the tubes and reducing overall internal resistance. (3) Binder-free attachment of the CNT micro-conduits to carbon cloth fibers by GP interconnection reduces interfacial resistance, and also allows electrolyte ions to diffuse easily through the hollow channels of the micro-conduits. (4) The detailed modeling results show that the unique leaves-on-branchlet nanostructure with sharp edges present significantly higher surface charge density than basal regions, indicating that the edges contribute significantly more to capacitance than basal regions; therefore, increasing edge density should facilitate better capacitive performance, which has been corroborated by experimental results reported by Bo. et al.^23^ The non-uniform characteristics of the charge, counter-ion (SO_4_^2–^), and co-ion (H^+^) distributions (Fig. 5) are consistent with DFT and MD calculations provided by Bo. et al.^23^ Such a substantial charge concentration at the edges benefits electrolyte ionic penetration into GP interiors and enhances accessibility of electrolyte, leading to higher areal capacitance compared to the bare CNT case. The simulation results, which predict a 6.5-fold enhancement in areal capacitance, are qualitatively consistent with the foregoing experimental measurements (10-fold enhancement). The discrepancies in the areal capacitance between the numerical calculation and experimental measurements may be because our NPP calculations did not consider the contribution from mechanical integrity (e.g., cracking, collapse, and shedding of bare CNT micro-conduits) and chemical reactions observed in the experiments. In order to elucidate charge transfer mechanism at the nanoscale with higher modeling accuracy, dimensionless Poisson and Langevin equations^50^ that can capture such size effects at the nanoscale will be formulated in the future models.

Moreover, CNT/GP micro-conduit electrodes have been demonstrated as efficient nanotemplates for highly pseudocapacitive materials in foregoing sections. Since energy density is proportional to capacitance (*C*) and the square of voltage (*V*^*2*^), energy-power curves of CNT/GP micro-conduit pseudocapacitors are expected to move toward the upper right corner of the Ragone plot shown in Fig. 4f, achieving higher energy and power densities. For example, in Supplementary Fig. 25, we have demonstrated a snapshot for such asymmetric supercapacitors based on CNT/GP/Ni-Co hydroxide micro-conduits as positive electrodes in 2 M KOH aqueous electrolyte. Although not optimized yet, the asymmetric device exhibits 8.7 mWh cm^−3^, high rate capability, and low internal resistance (Supplementary Fig. 25). We believe that electrochemical performance of CNT/GP micro-conduit asymmetric supercapacitors can be further improved by choosing appropriate high-performance pseudocapacitive materials, negative electrode, and electrolyte.

In summary, the present results demonstrate that hybrid CNT/GP micro-conduits in a bioinspired leaves-on-branchlet structure are promising as active materials for high-performance supercapacitors and efficient nanotemplates for pseudocapacitors with outstanding performance. The novel all-carbon micro-conduits in a bioinspired leaves-on-branchlet nanostructure presented in this work is particularly well-suited for practical implementation as high-performance symmetric and asymmetric supercapacitor electrodes, indicating great potential in the applications for energy storage.

## Methods

### Synthesis of CNT/GP micro-conduits

All-carbon CNT/GP micro-conduits were fabricated by a two-step process. First, commercial plain carbon cloth (CC, Fuel Cell Earth LLC) was used directly as the substrate for CNT micro-conduit synthesis by MPCVD. Before the growth, Ti/Al/Fe tri-layer catalysts (nominally 30/10/5 nm) were deposited on both sides of CC substrates by e-beam physical vapor deposition at a base pressure of 5 × 10^−7^ Torr. During the growth, H_2_ (50 sccm) and CH_4_ (10 sccm) were introduced as gas sources for CNT growth, with a total pressure of 10 Torr. The plasma power during the growth was 300 W, and the growth time was 10 min. CNT mass density was measured to be 0.9 mg cm^−2^. Subsequently, GPs were further grown on CNT micro-conduits in the same MPCVD system with a condition of H_2_ (50 sccm) and CH_4_ (10 sccm) as the primary feed gases at 30 Torr total pressure. The plasma power was 600 W during the GP growth process. With a total GP growth time of 18 min, GP areal mass density was 3.85 mg cm^−2^. The preparation details are provided in Supplementary Methods.

### Material characterization

The morphology and microstructure of electrodes were characterized by field emission scanning electron microscope (SEM, Hitachi S-4800) operated at 5 kV and transmission electron microscopy (TEM, Japan FEM-2100F) with an accelerating voltage of 200 kV. TEM samples for CNT/GP micro-conduit structure analysis were prepared by scratching a sample surface with a razor blade to remove the deposited material into a vial with acetone followed by ultrasonic bath treatment for several minutes, after which a drop of the obtained suspension was cast onto a lacey carbon 300 mesh copper TEM grid. Raman characterization was performed with LabRAM HR spectrometer (HORIBA Scientific) with a fixed laser excitation wavelength of 532 nm, power of 5 mW, spot size of ~1 μm, and magnification of 50 ×.

### Electrochemical measurements

Detailed fabrication procedures of CNT/GP/PANI and CNT/GP/Ni-Co hydroxide electrodes are provided in Supplementary Methods. The electrochemical performance of electrodes was evaluated using a Gamry Echem Testing System (Gamry Instruments, Inc., USA) in a three-electrode configuration at room temperature. A CNT/GP (or CNT/GP/PANI) micro-conduit electrode served as the working electrode using 1 M H_2_SO_4_ as electrolyte. Pt mesh and Ag/AgCl were used as the counter and reference electrodes, respectively. A SCE reference electrode was used to test CNT/GP/Ni-Co hydroxide electrodes in 2 M KOH electrolyte. Symmetric and asymmetric supercapacitor devices consisting of CNT/GP micro-conduit electrodes were fabricated (see preparation details in Supplementary Methods) and tested at room temperature. Symmetric supercapacitors were electrochemically characterized in a two-electrode configuration cell in 1 M H_2_SO_4_ aqueous electrolyte. The methods to calculate specific capacitances, energy, and power densities are provided in Supplementary Methods.

### NPP modeling

NPP calculations of the Gouy–Chapman model were employed to elucidate the underlying mechanisms of charge transfer and storage, as well as ion diffusion, governed by the Poisson equation and the Nernst–Plank equation, respectively. The governing equations were simultaneously solved with an implicit algorithm for the purpose of iterative coupling by the finite element (FE) method using the commercial software COMSOL 5.2. The FE-based NPP calculations run with the inputs of properties from the MD simulations (the schematic representation of the MD simulation systems has been provided in Supplementary Fig.26) to render the atomically informed modelling. The simulation details are provided in Supplementary Methods.

### Data availability

The data that support the findings of this study are available from the corresponding authors upon request.

## Electronic supplementary material

Supplementary Information

Peer Review File
