# Supplementary material for: Bioinspired leaves-on-branchlet hybrid carbon nanostructure for supercapacitors
Source: Nat Commun. 2018 Feb 23;9:790. doi: 10.1038/s41467-018-03112-3 (PMC5824788; doi:10.1038/s41467-018-03112-3)
Supplement: Supplementary file 1 — Supplementary Information [file 41467_2018_3112_MOESM1_ESM.pdf]

Supplementary Information

Supplementary Figures

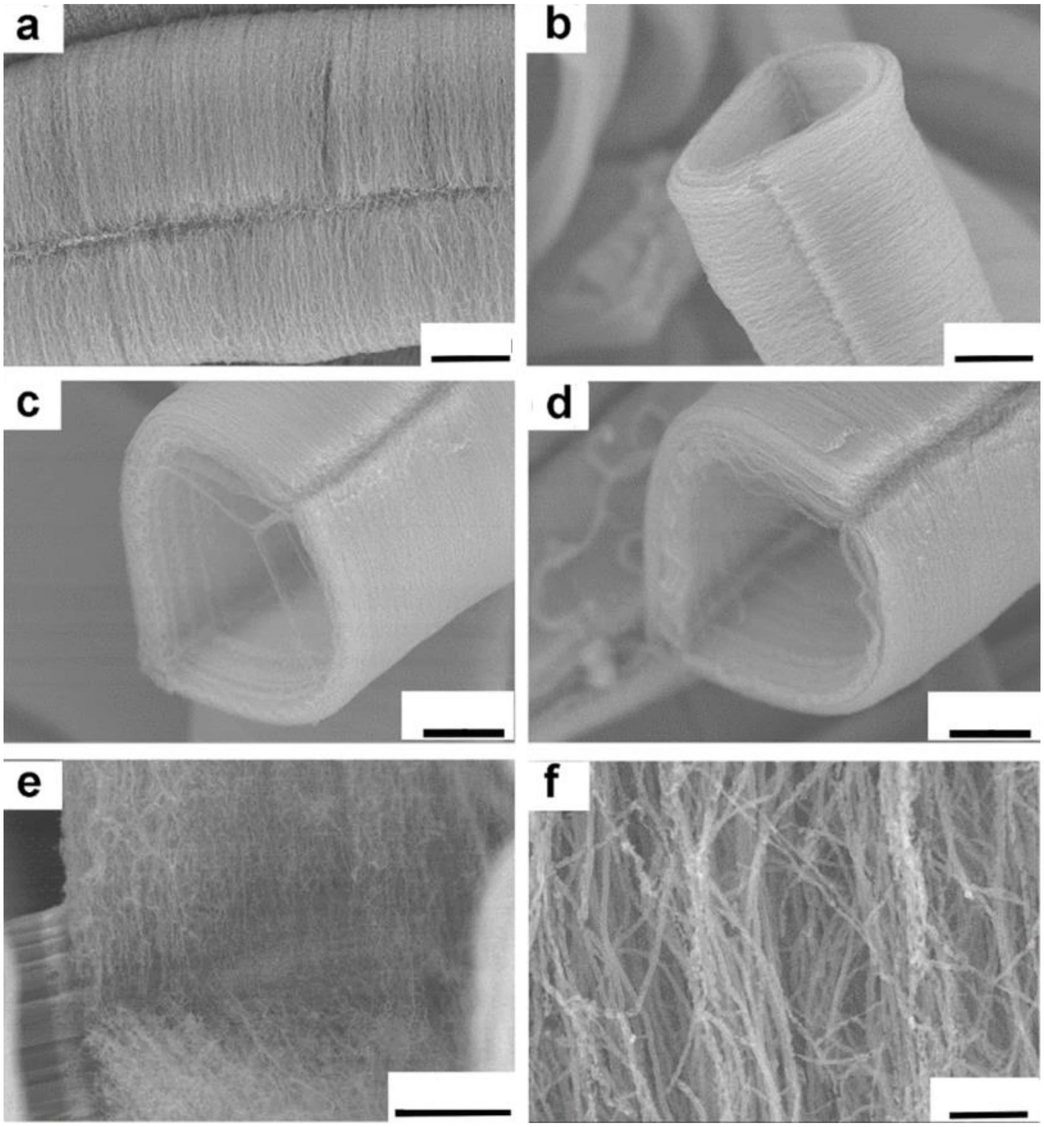

Supplementary Figure 1│SEM images of CNT micro-conduit structures. (a, b) A typical CNT micro-conduit, and (c, d) CNT micro-conduits in a heart shape. (e)The interface between a carbon microfiber and conduit walls. (f) CNTs within micro-conduits at high magnification. Scale bars: (a, c, d) 10 μm, (b) 15 μm, (e) 5 μm, (f) 400 nm.

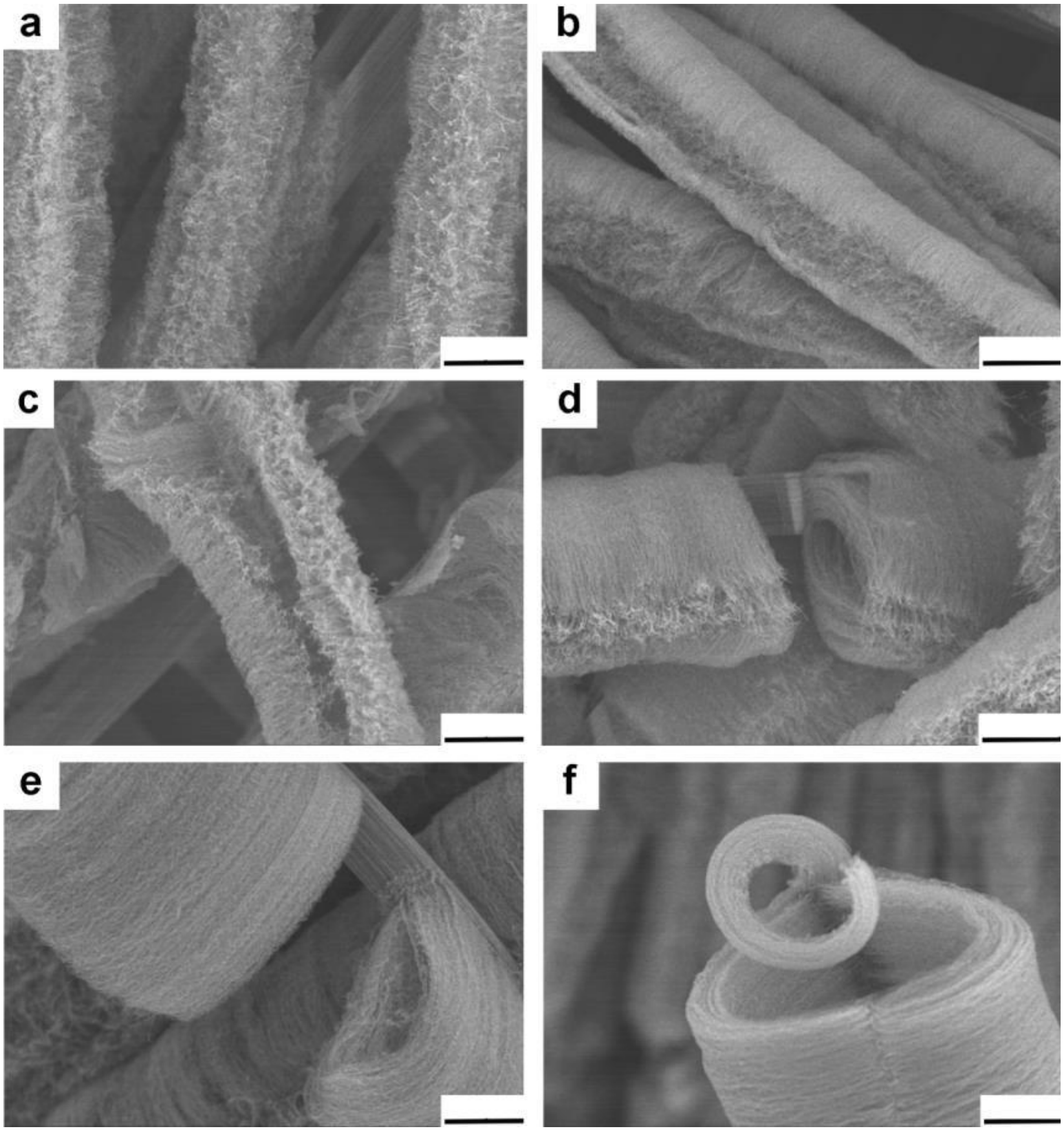

Supplementary Figure 2 | CNTs grown on carbon cloth under different growth durations. (a) 1 min, (b) 2min, (c) 3 min, (d) 5 min, (e) 7 min, and (f) 10 min. Scale bars: (a)–(f) 10 μm.

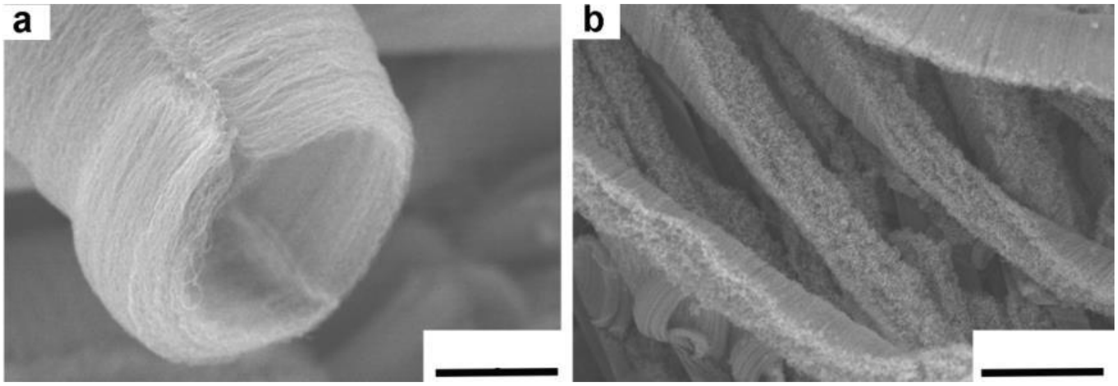

Supplementary Figure 3 │ SEM images of CNTs on CC under different plasma conditions. (a) With plasma, (b) with plasma shielded. Scale bars: (a)–(b) 20 μm.

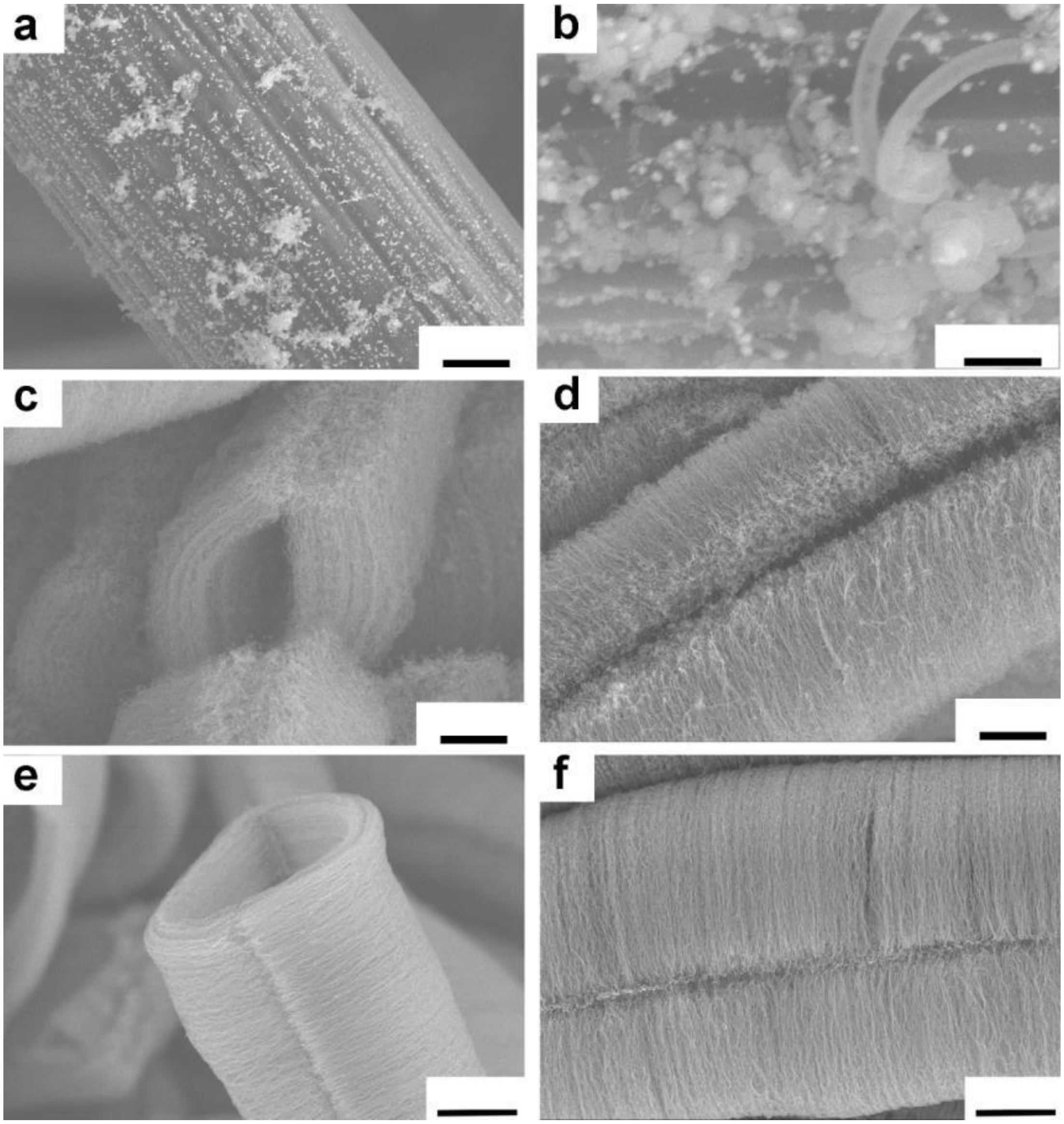

Supplementary Figure 4 | SEM to show morphologies of CNTs using different catalysts: (a, b) Only Fe as catalyst. (c, d) Ti/Fe as catalyst. (e, f) Ti/Al/Fe as catalyst. Scale bars: (a) 2 μm, (b) 500 nm, (c, d, f) 10 μm, (e) 15 μm.

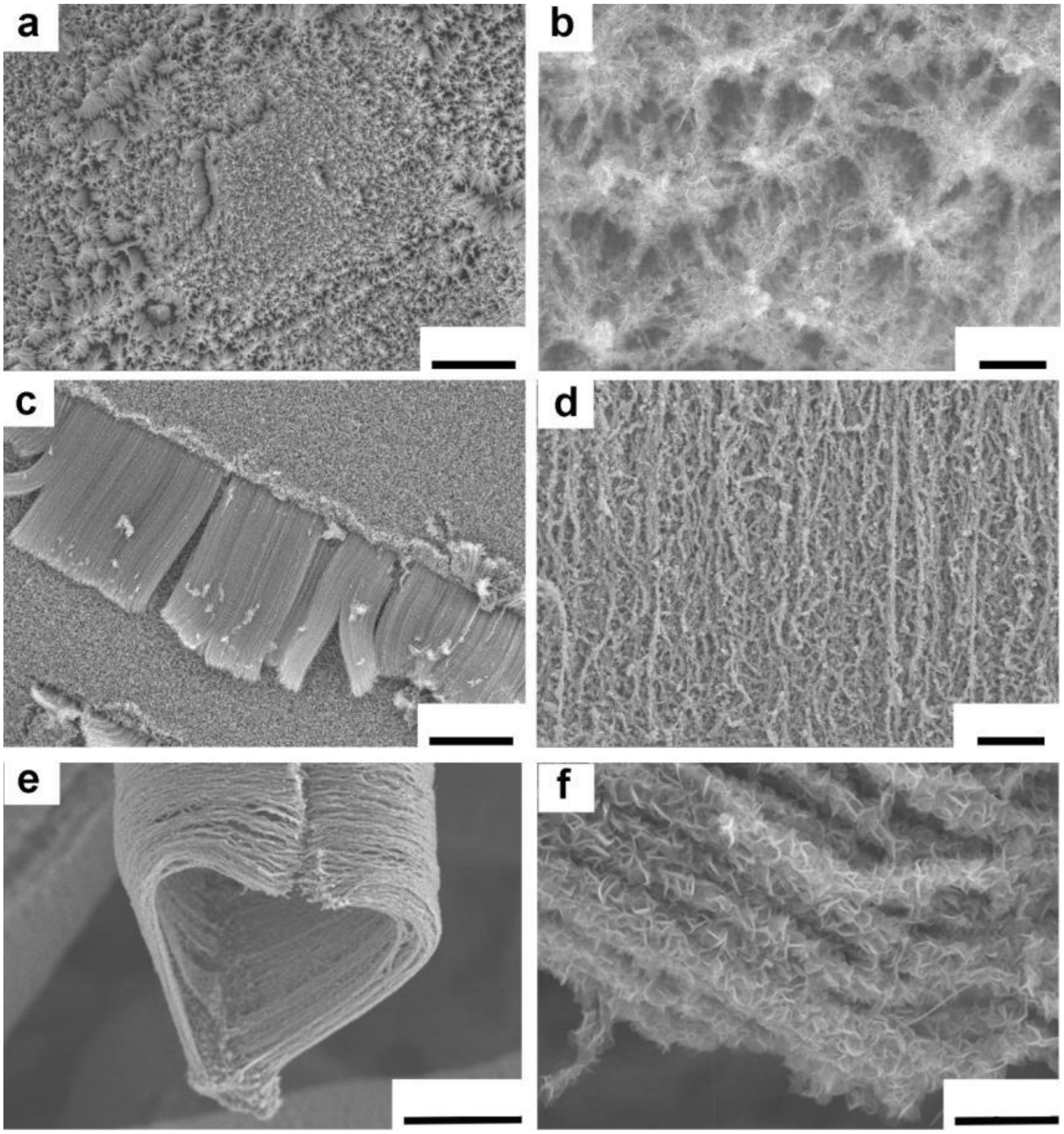

Supplementary Figure 5 | SEM images of morphologies of CNTs grown on various substrates with further decoration of GPs: Buckypaper as substrate at (a) low magnification and (b) high magnification. Si wafer as substrate at (c) low magnification and (d) high magnification. Carbon cloth as substrate at (e) low magnification and (f) high magnification. Scale bars: (a, c) 30 μm, (b, f) 2 μm, (d) 5 μm, (e) 20 μm. CNT micro-conduits successfully formed only when using carbon cloth as a substrate, within which carbon microfibers exhibit a diameter of several micrometers. The catalyst layers (Ti/Al/Fe: 30/10/5 nm) were deposited on both sides of the carbon cloth substrates, resulting in a gradually varying thickness of catalyst layers around carbon fibers because of the cylindrical structure of the carbon fibers. The different thicknesses of catalyst layers lead to different sizes of catalyst nanoparticles, resulting in different catalytic activity and thus different CNT growth rates and morphologies.

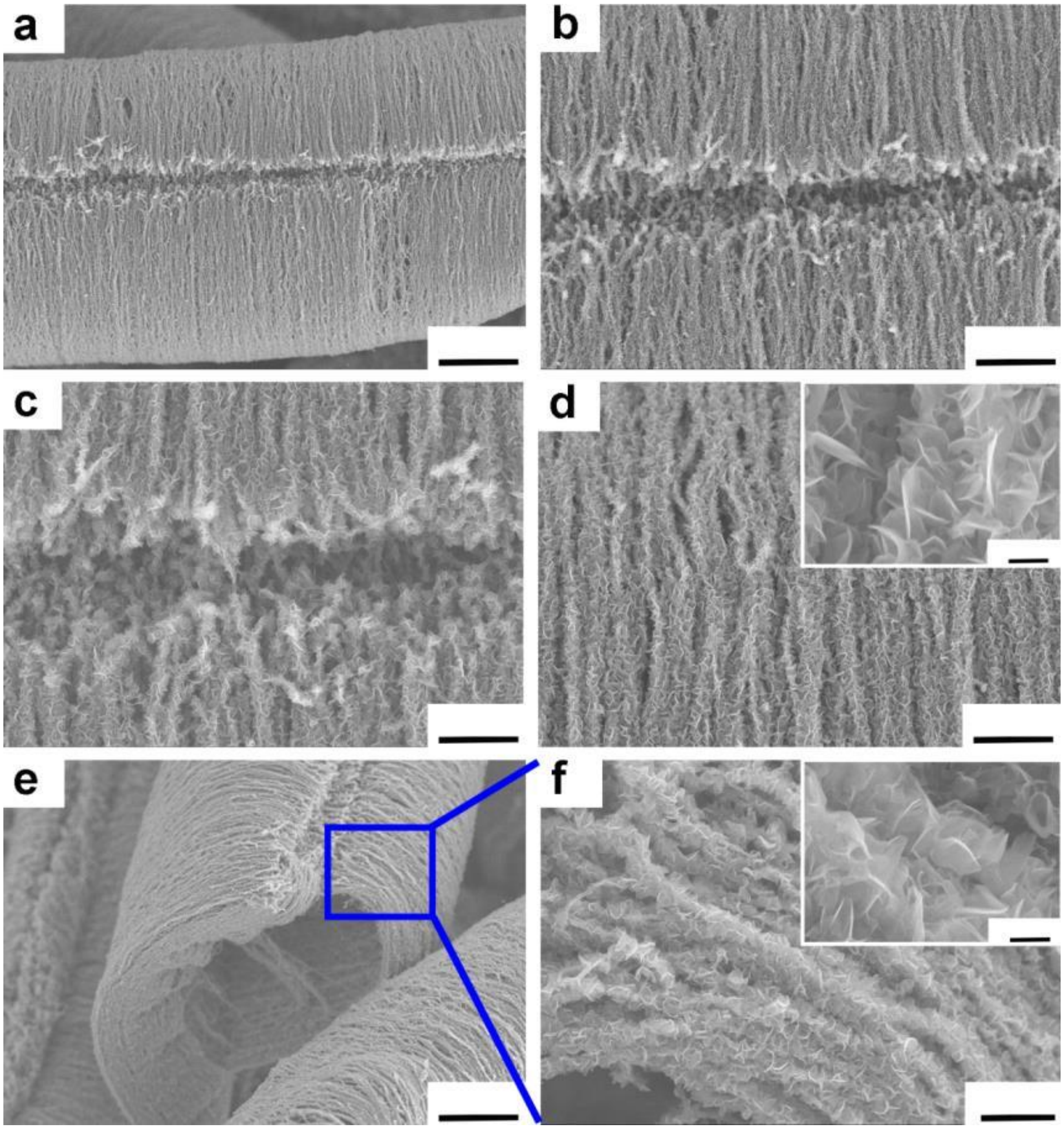

Supplementary Figure 6│SEM images of CNT/GP micro-conduits. (a) Top view of a CNT/GP micro-conduit. (b) Seam structure at the top of a CNT/GP micro-conduit. (c) Seam structure at high magnification. (d) GPs grown on CNT micro-conduits (the inset shows GPs with sharp edges). (e) Tilted cross-section view of CNT/GP micro-conduits. (f) Magnified image of the blue box in (e) to show uniform coverage of GPs on CNT micro-conduits (the inset displays GPs at high magnification). Scale bars: (a, e) 10 μm, (b) 4 μm, (c) 2 μm, (d) 2 μm (inset: 200 nm), (f) 1 μm (inset: 200 nm).

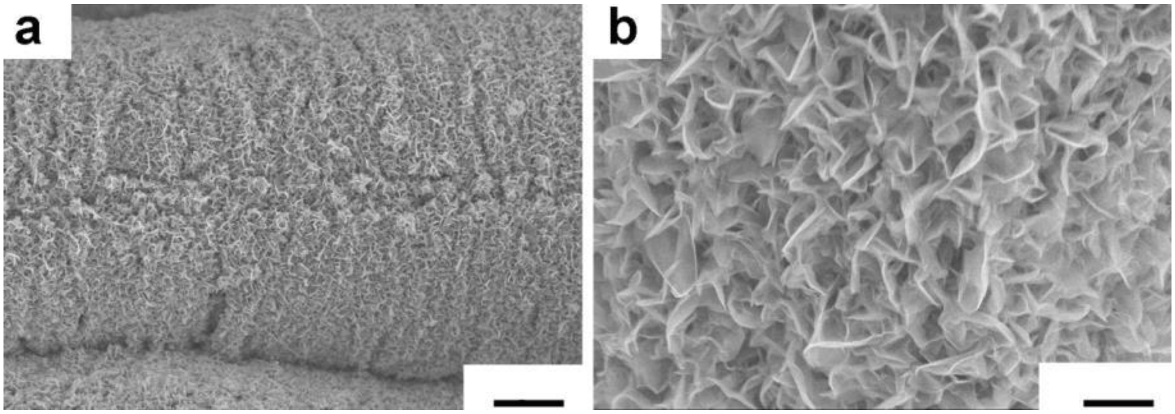

Supplementary Figure 7│SEM images of CNT/GP micro-conduits with a growth duration of 35 min. (a) CNT/GP micro-conduit structure at low magnification. (b) GPs with sharp edges on CNT micro-conduits at high magnification. Scale bars: (a) 5 μm, (b) 300 nm.

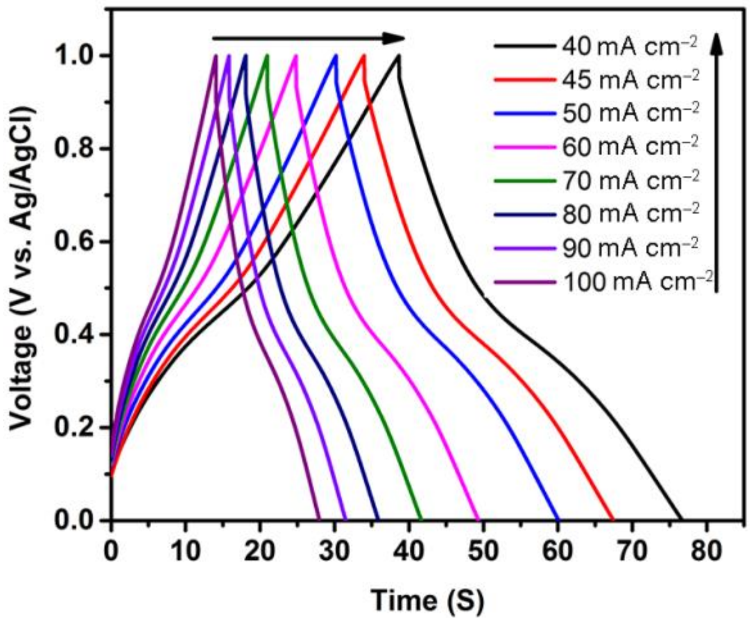

Supplementary Figure 8│Galvanostatic charge/discharge profiles of CNT/GP micro-conduit electrodes at high current densities ranging from 40 to 100 mA cm^{-2} between 0 to 1 V vs. Ag/AgCl.

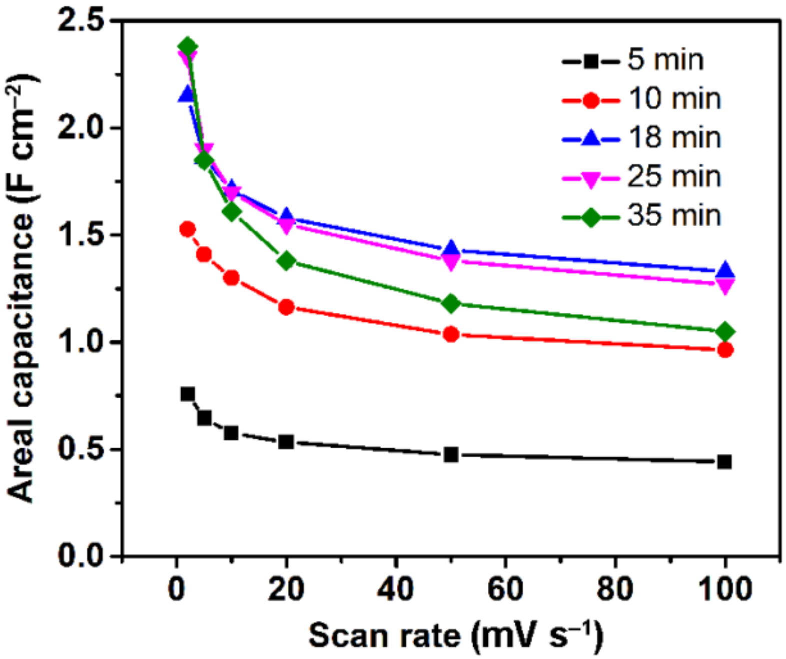

Supplementary Figure 9 | Areal capacitances of electrodes with different GP growth durations (5, 10, 18, 25 and 35 min) as a function of scan rates. The results reveal a relationship between the edge density of GPs and capacitance of CNT/GP micro-conduit electrodes, and indicate that an optimal GP growth time should exist for superior electrochemical performance because of a balance between surface area and ion transfer kinetics.

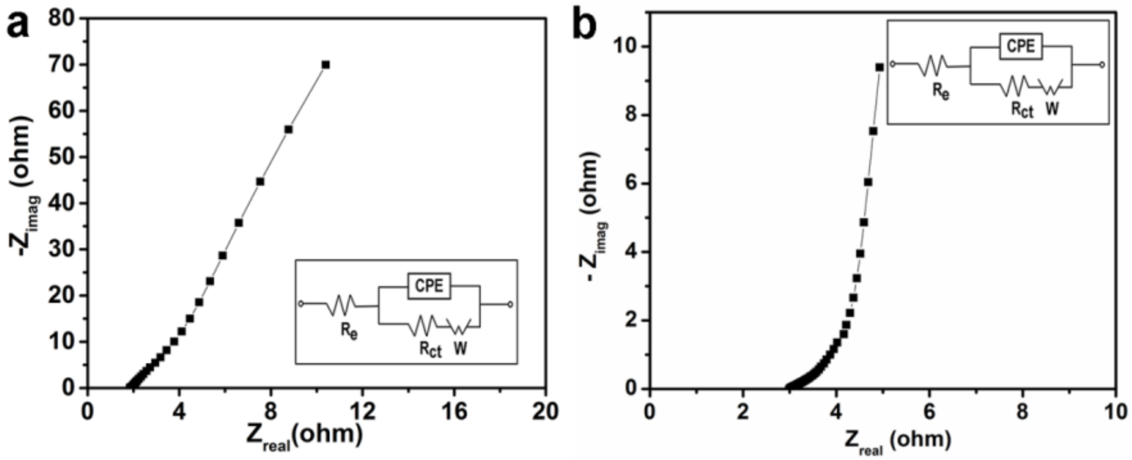

Supplementary Figure 10 │ (a) Nyquist plot recorded from 0.1 Hz to 1 MHz for a CNT micro-conduit electrode (the inset shows the equivalent circuit). (b) Nyquist plot recorded from 0.1 Hz to 1 MHz for a CNT/GP micro-conduit electrode. The bulk electrolyte resistance R_e of CNT and CNT/GP micro-conduit electrodes is measured to be 1.85 and 2.8 Ω, respectively. Moreover, negligible semicircles in the high frequency region from impedance spectra of both electrodes indicate a low charge transfer resistance R_{ct} of CNT and CNT/GP micro-conduit electrodes.

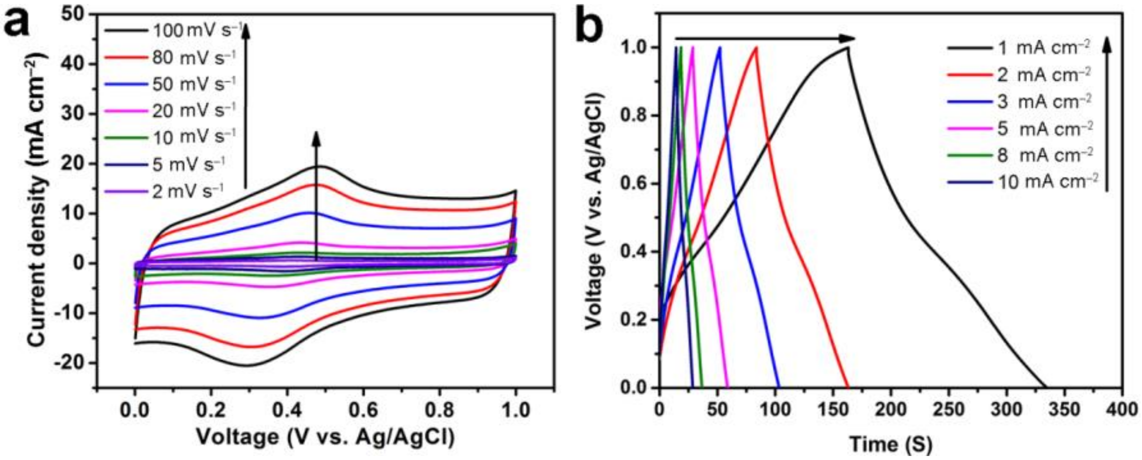

Supplementary Figure 11│Electrochemical performance of CNT micro-conduit electrodes in a three-electrode configuration with 1 M H_2SO_4 aqueous solution. (a) CV curves at scan rates from 2 to 100 mV s^{-1} in the voltage window between 0 and 1 V vs. Ag/AgCl. (b) Galvanostatic charge/discharge curves at current densities from 1 to 10 mA cm^{-2} in the voltage window between 0 and 1 V vs. Ag/AgCl. The capacitance of the CNT micro-conduit electrode calculated from the charge/discharge curves is 0.167 F cm^{-2} at a current density of 1 mA cm^{-2}.

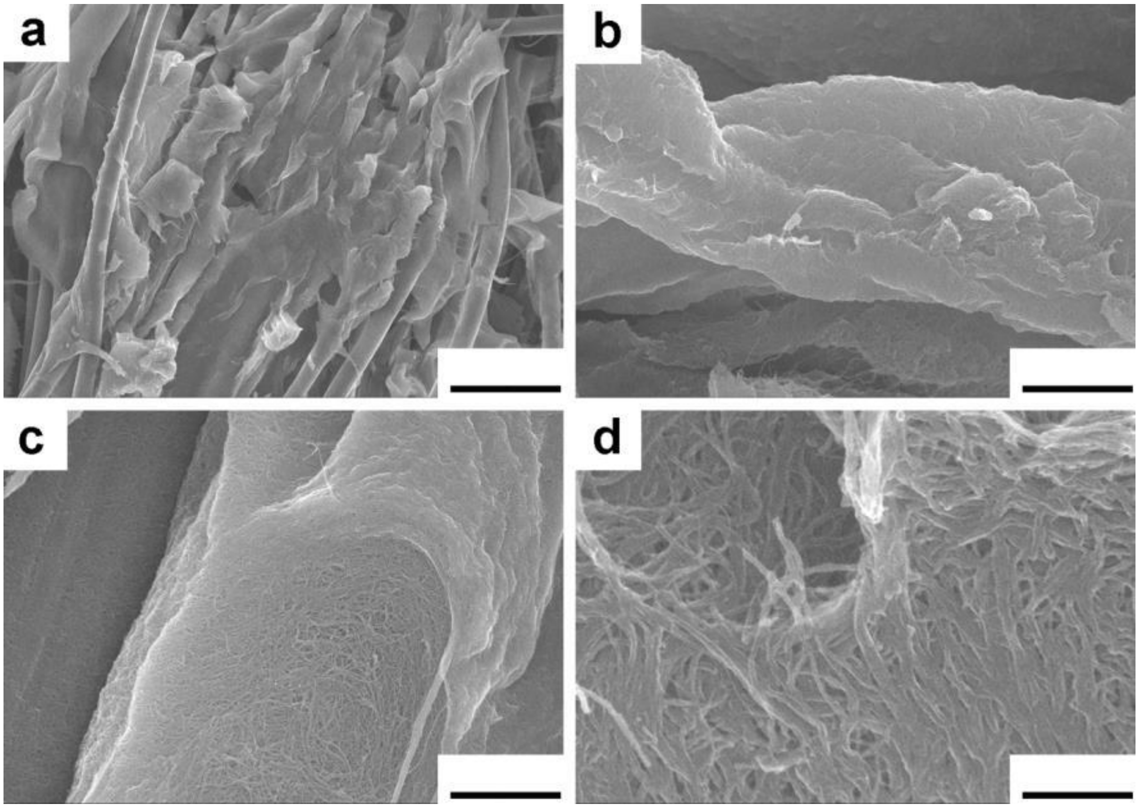

Supplementary Figure 12│SEM images of bare CNT micro-conduits on CC after electrochemical oxidation. (a) CNTs on CC at low magnification. (b) CNTs at high magnification. (c) CNT aggregation on carbon fiber surface. (d) Aggregated and collapsed CNT layers on a carbon fiber surface at high magnification. Scale bars: (a) 50 μm, (b, c) 5 μm, (d) 500 nm.

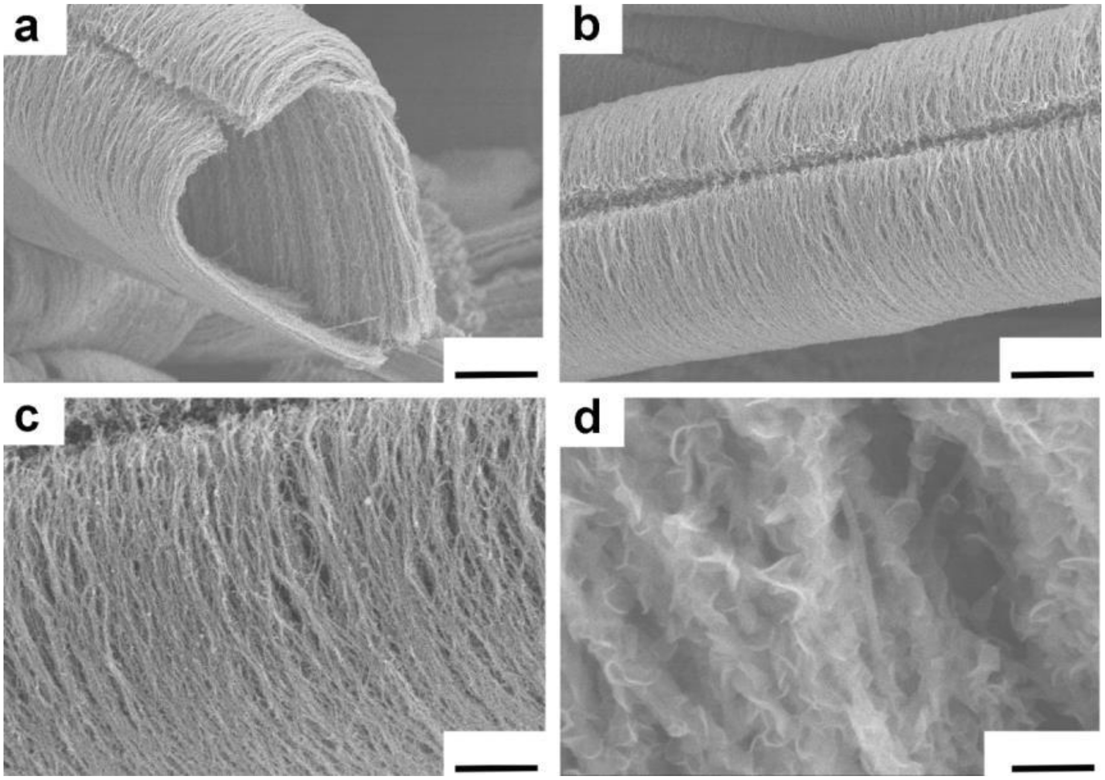

Supplementary Figure 13│SEM images of the CNT/GP micro-conduit structure after treatment in strong acids consisting of H_2SO_4 (assay 96 %) and HNO_3 (assay 70 %) (v/v = 3:1). (a) A typical treated CNT/GP micro-conduit structure in a heart shape. (b) Top view of a treated CNT/GP micro-conduit. (c) GPs grown on CNT micro-conduit walls after treatment. (d) Leaves-on-branchlet structure at high magnification after treatment. Scale bars: (a, b) 10 μm, (c) 4 μm, (d) 400 nm.

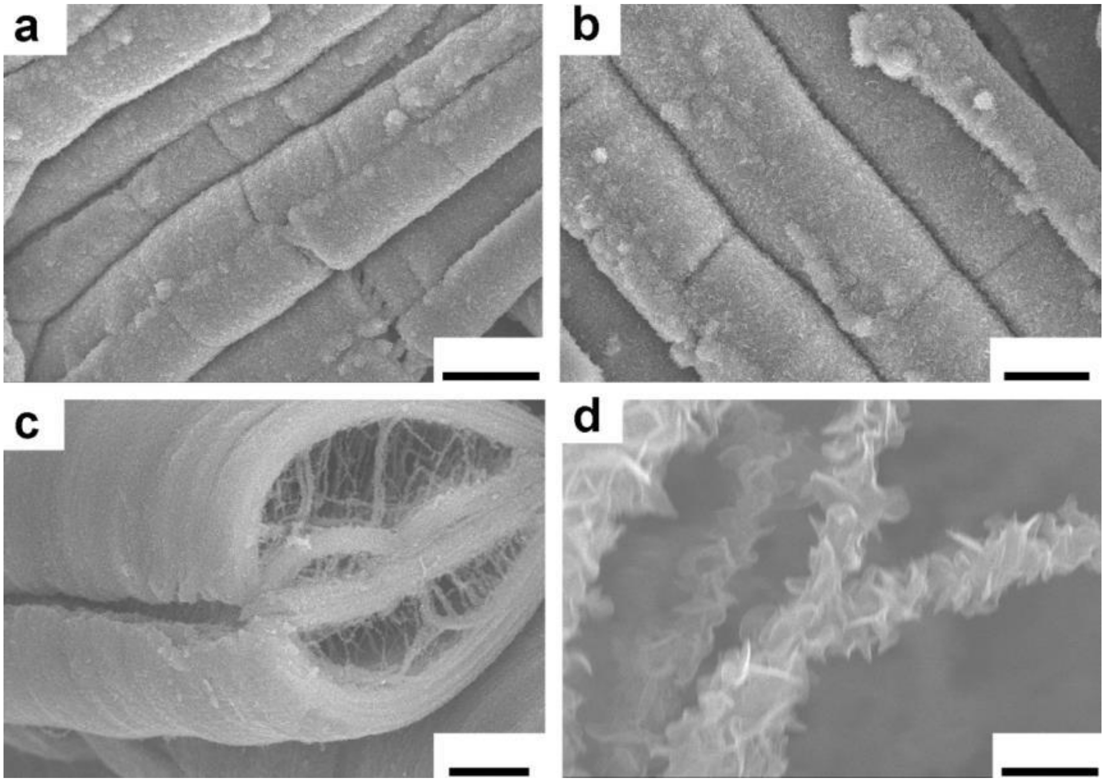

Supplementary Figure 14│SEM images of the CNT/GP micro-conduit structure after ultrasonic treatment for 30 mins. (a, b) Top views of treated CNT/GP micro-conduits. (c) A typical treated CNT/GP micro-conduit structure in a heart shape. (d) Leaves-on-branchlet structure at high magnification after treatment. Scale bars: (a) 30 μm, (b) 20 μm, (c) 10 μm, (d) 1 μm.

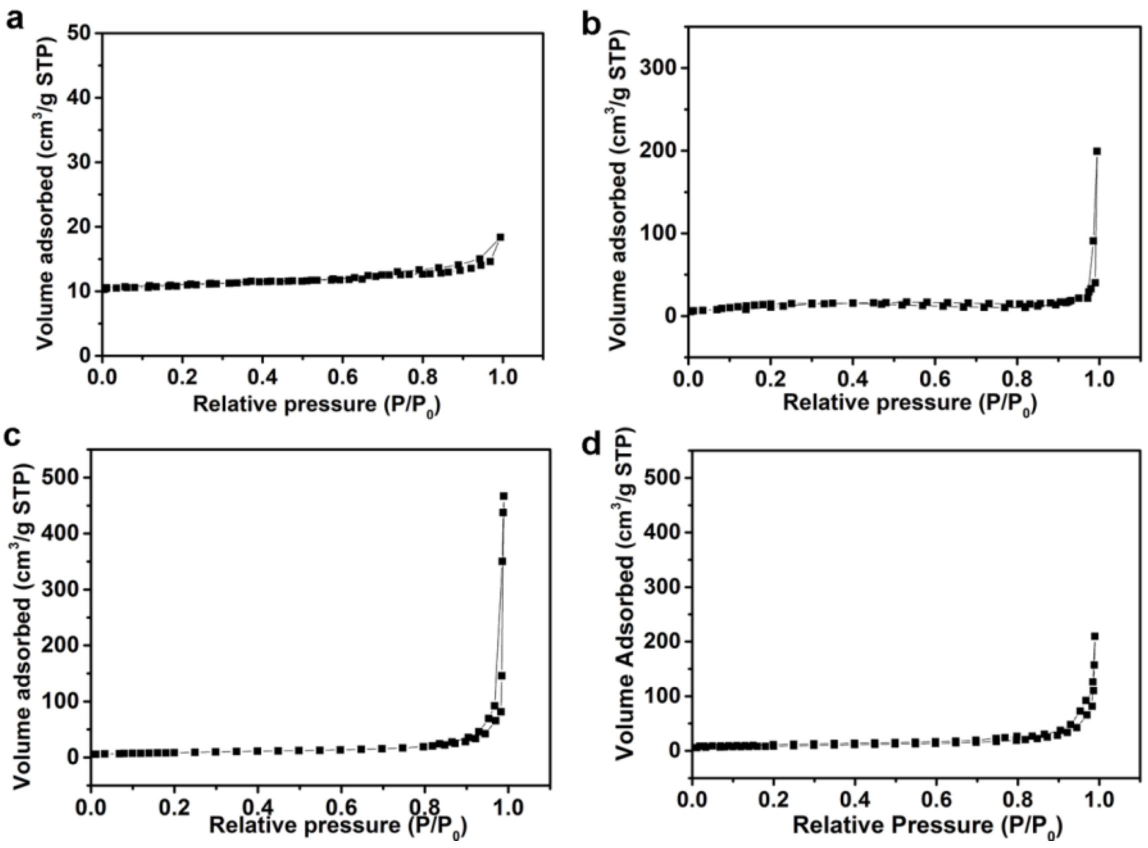

Supplementary Figure 15 | Nitrogen (N_2) adsorption-desorption isotherm curves of (a) bare CNT micro-conduit electrode, (b) CNT/GP micro-conduit electrode with GP growth time of 10 min, (c) CNT/GP micro-conduit electrode with GP growth time of 18 min, (d) CNT/GP micro-conduit electrode with GP growth time of 45 min.

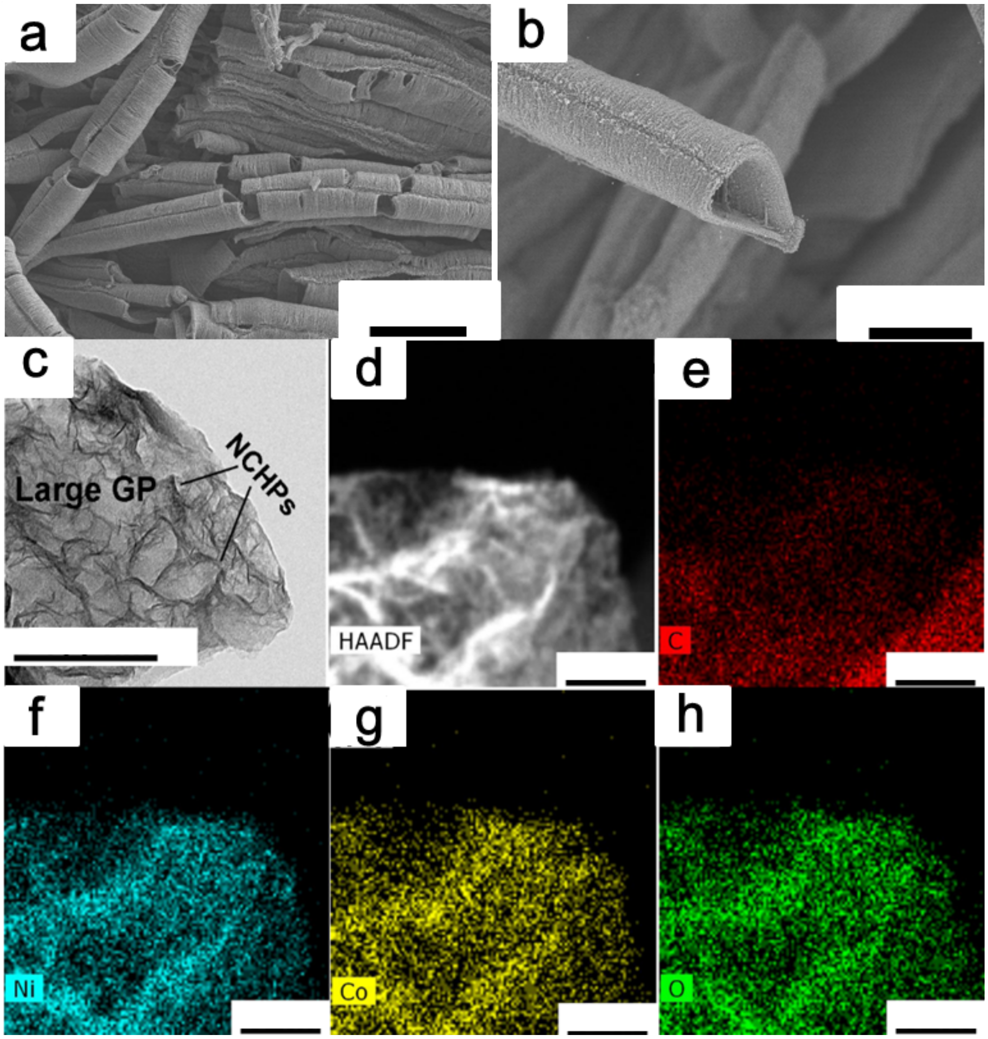

Supplementary Figure 16 | Microstructure of Ni-Co hydroxide petals (NCHPs) electro-deposited on CNT/GP micro-conduit: (a) SEM image of CNT/GP/NCHP micro-conduits at low magnification. (b) SEM image of typical " heart-shape " morphology after NCHP deposition. (c) TEM image of a GP covered with NCHPs. (d) High-angle annular dark field (HAADF) scanning TEM image of a magnified part of a GP covered with many small NCHPs. (e-h) Elemental mapping showing uniform spatial distribution of elements in (d): (e) to (h) correspond to C, Ni, Co and O element maps, respectively. Scale bars: (a) 50 μm, (b) 15 μm, (c) 100 nm, (d)–(h) 20 nm.

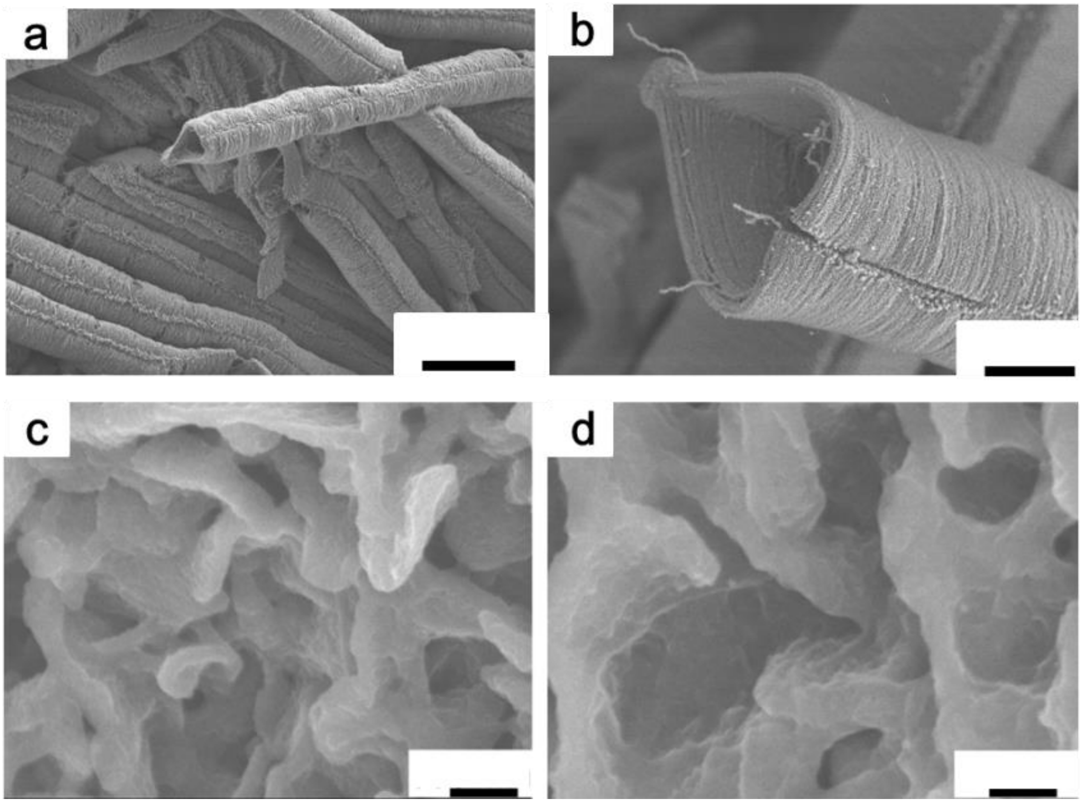

Supplementary Figure 17 | SEM images of CNT/GP/PANI micro-conduits: (a) At low magnification. (b) A typical micro-conduit in a heart-shape. (c) Uniform PANI coating on GP surface. (d) Rough surface of the electrode after electrodeposition of PANI. Scale bars: (a) 50 μm, (b) 15 μm, (c) 300 nm, (d) 200 nm.

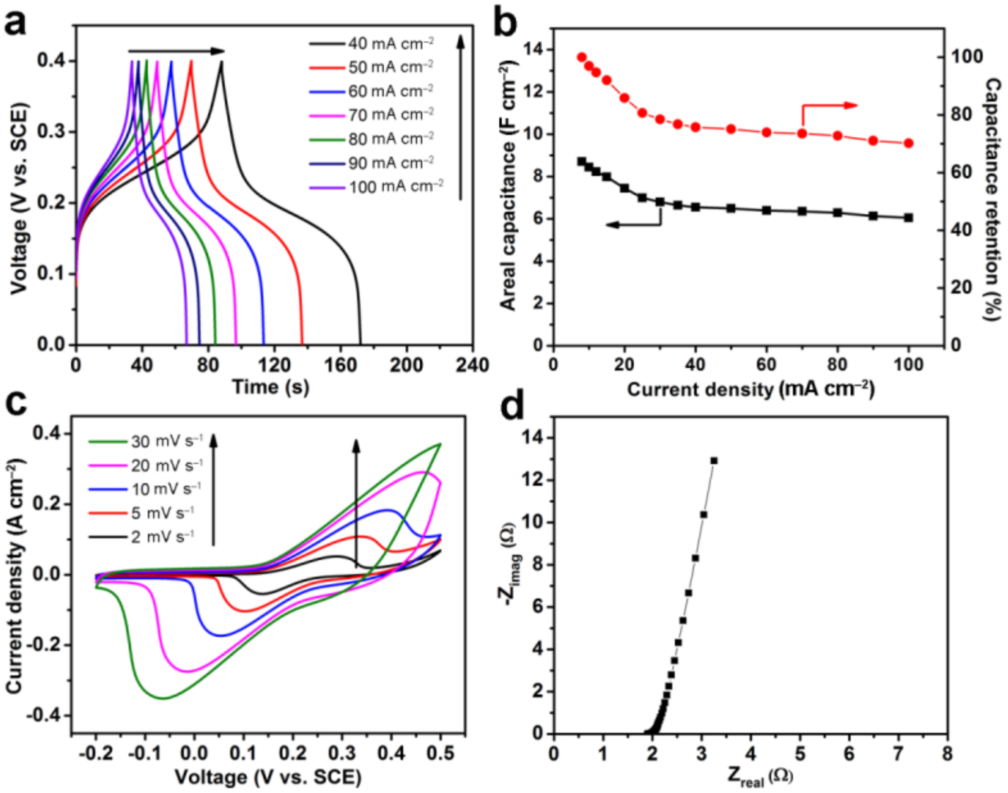

Supplementary Figure 18 | Electrochemical performance of the CNT/GP/Ni-Co hydroxide micro-conduit electrode. (a) Galvanostatic charge/discharge curves at high current densities from 40 to 100 mA cm^{-2}. (b) Areal capacitances and capacitance retention as a function of current density calculated from the galvanostatic charge/discharge curves. (c) CV curves at scan rates from 2 to 30 mV s^{-1} with a voltage window from –0.2 to 0.5 V vs. SCE. (d) Nyquist plot recorded from 0.1 Hz to 1 MHz. The R_e calculated from the impedance spectrum is 1.8 Ω, and the negligible R_{ct} value indicates low electrical resistivity of the hybrid electrodes.

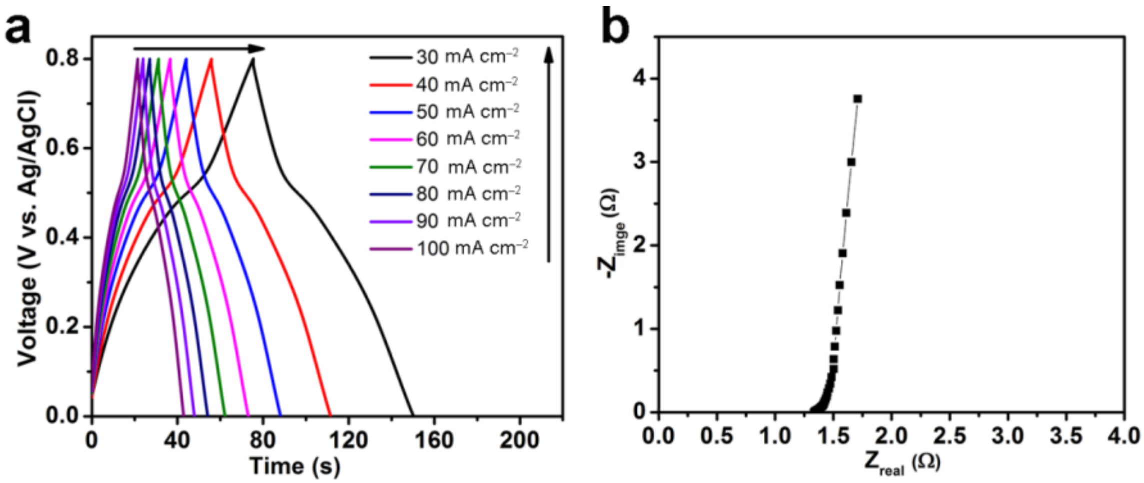

Supplementary Figure 19│Electrochemical performance of the CNT/GP/PANI micro-conduit electrode. (a) Galvanostatic charge/discharge curves at high current densities from 30 to 100 mA cm^{-2}. (b) Nyquist plot recorded from 0.1 Hz to 1 MHz. The internal resistance for the electrode calculated from the impedance spectrum is approx. 1.3 Ω.

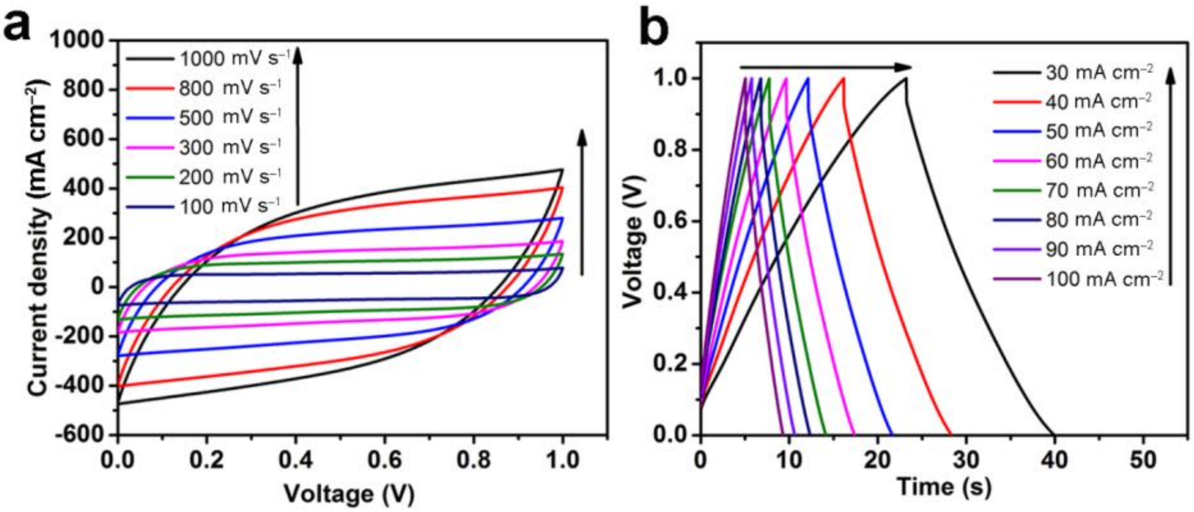

Supplementary Figure 20 | (a) CV curves of the symmetric supercapacitor device at scan rates from 100 to 1000 mV s^{-1} with a voltage window from 0 to 1 V. (b) Galvanostatic charge/discharge curves of the symmetric supercapacitor device at high current densities from 30 to 100 mA cm^{-2} in the voltage range between 0 and 1 V.

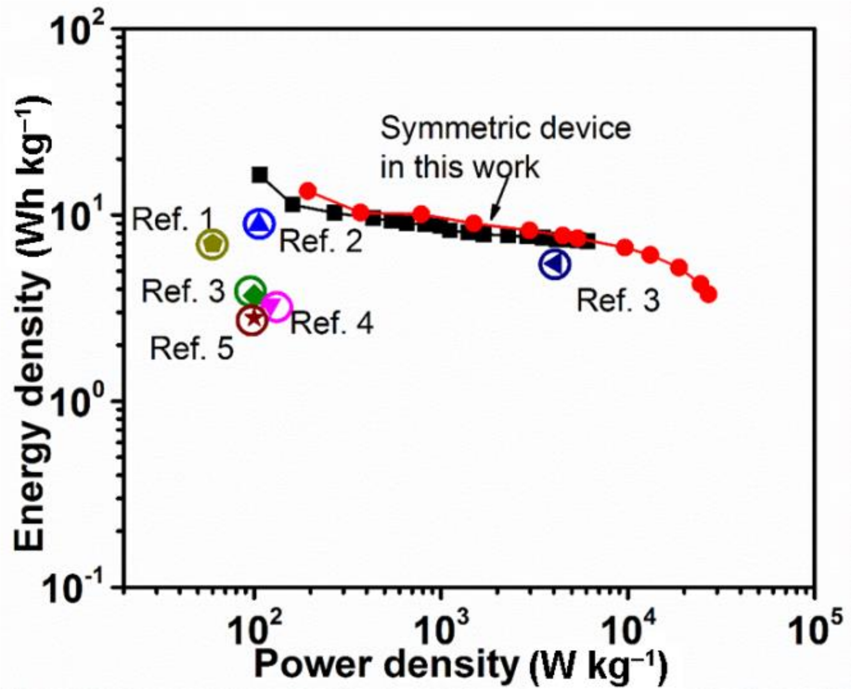

Supplementary Figure 21 | Comparative Ragone plot (based on active material mass) of contemporary energy devices and the present symmetric CNT/GP micro-conduit device. The energy and power densities of the preset device were calculated using both cyclic voltammetry (CV, in red circle) and constant-current charge/discharge (CD, in black rectangle) methods. The results calculated from the two methods agree well with each other. Energy density of the present symmetric device reaches up to 16.4 Wh kg^{-1} (calculated based on the CV results), and the device also deliver a power density up to 27 kW kg^{-1} (calculated based on the CD results), which are significantly higher than those of contemporary carbon-based supercapacitors[1, 2, 3, 4, 5], indicating outstanding overall performance of the symmetric all-carbon supercapacitor in this work.

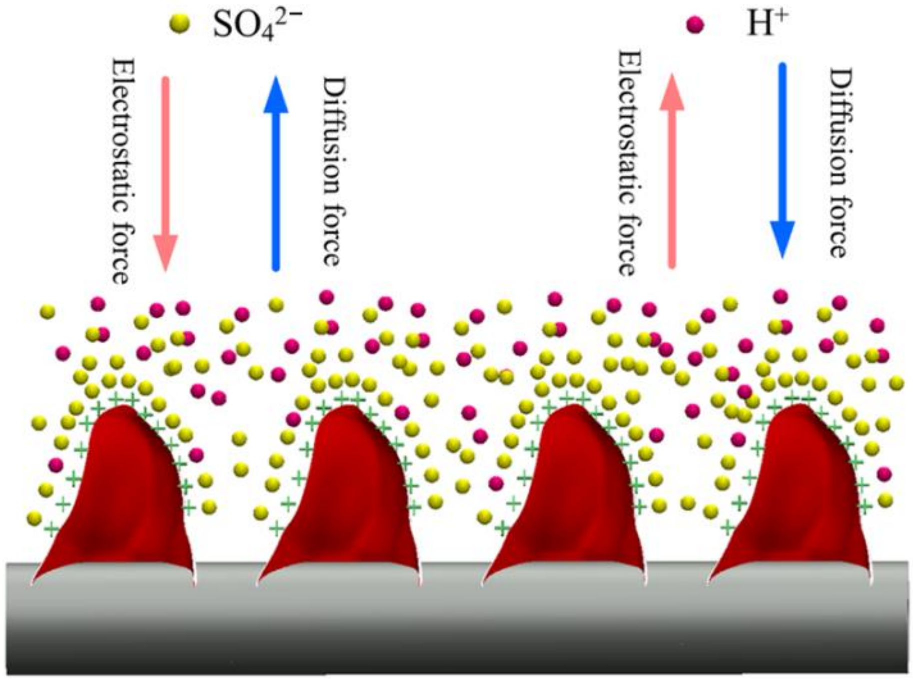

Supplementary Figure 22 │ Schematic of the ion transport and accumulation behavior, and the directions of diffusion and electrostatic driving forces for both counter-ions (SO_4^{2-}) and co-ions (H^+). During supercapacitor operation, the electrostatic force generally opposes the diffusion force. Equilibrium is achieved once the two driving forces reach a balance.

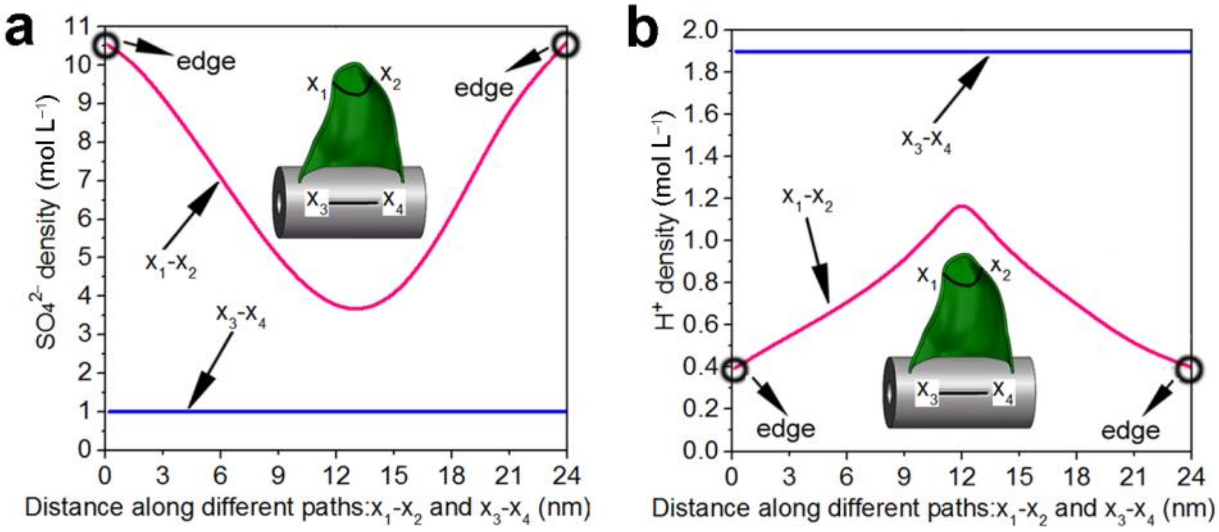

Supplementary Figure 23│ Ion distribution plots along chosen lines in CNT/GP micro-conduit electrodes with a leaves-on-branchlet structure in 1 M H_2SO_4 aqueous electrolyte subjected to an applied voltage of 1 V. (a) Counter-ion (SO_4^{2-}) distribution; (b) co-ion (H^+) distribution.

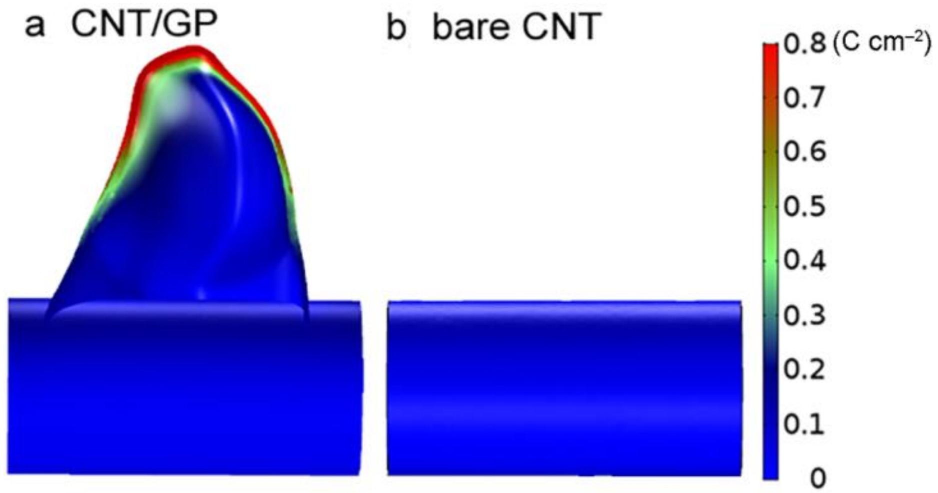

Supplementary Figure 24 | Comparison of surface charge density between the (a) CNT/GP and (b) bare CNT cases.

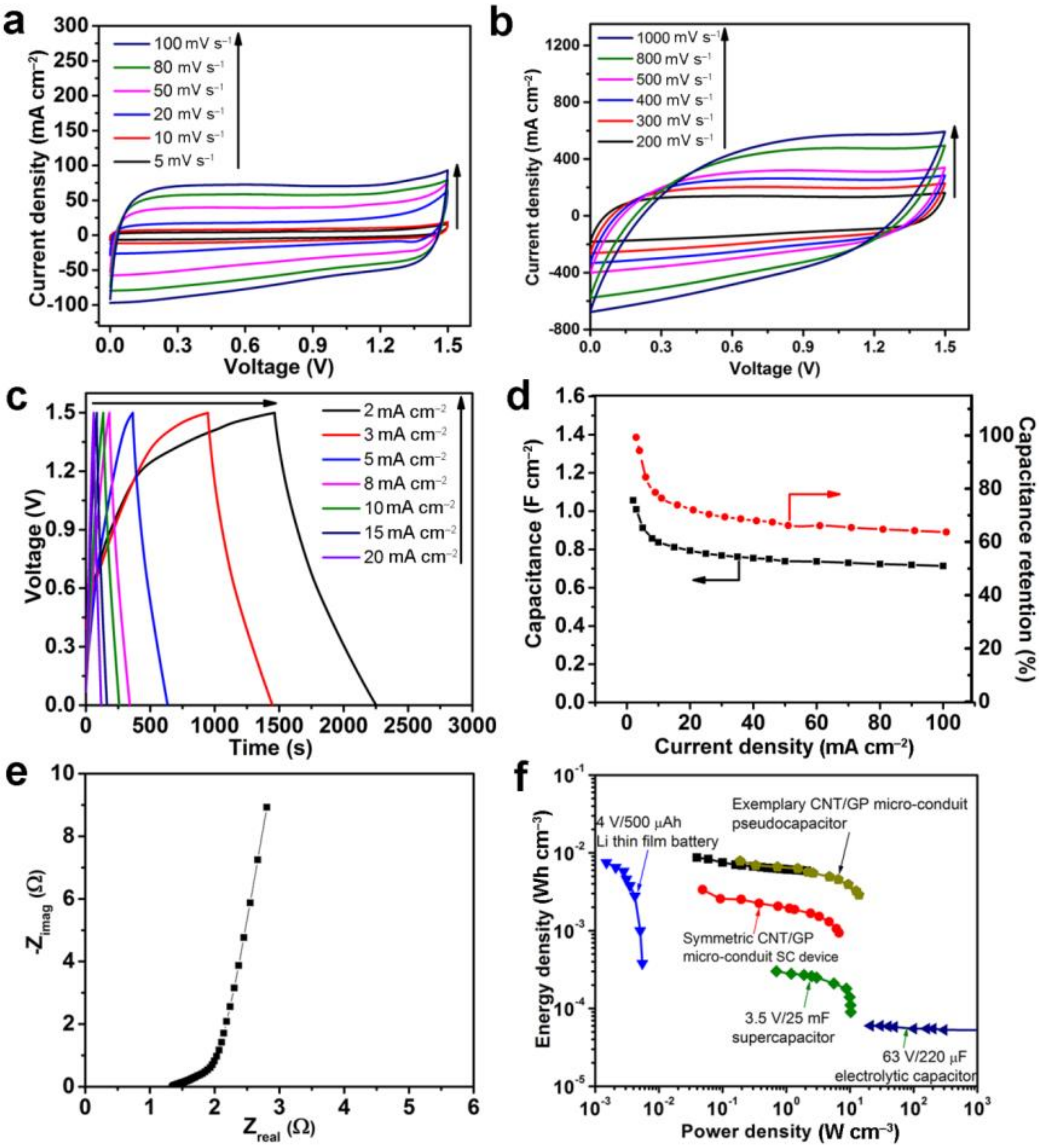

Supplementary Figure 25 | Electrochemical performance of the asymmetric device tested in a two-terminal configuration in 2 M KOH aqueous electrolyte solution. The asymmetric supercapacitor was assembled by sandwiching a commercial separator (Celgard™) between the positive and negative electrodes. A chemically treated electrode consisting of CNT/GP micro-conduits was used as the negative electrode, and the positive electrode is CNT/GP micro-conduits coated with Ni-Co hydroxide (electrodeposition time of 3 min). (a) CV curves at scan rates from 5 to 100 mV s^{-1} with a voltage window from 0 to 1.5 V. (b) CVs at high scan rates from 100 to 1000 mV s^{-1}. (c) Galvanostatic charge/discharge curves at current densities from 2 to 20 mA cm^{-2} in the voltage range between 0 and 1.5 V. (d) Capacitance and corresponding capacitance retention as functions of current density. (e) Nyquist plot recorded from 0.1 Hz to 1 MHz with an AC perturbation amplitude of 5 mV. (f) Comparative Ragone plot of the present asymmetric device (per volume of the fabricated device), a typical electrolytic capacitor, a Li-ion thin-film battery, a commercial supercapacitor (from ref. [6]).

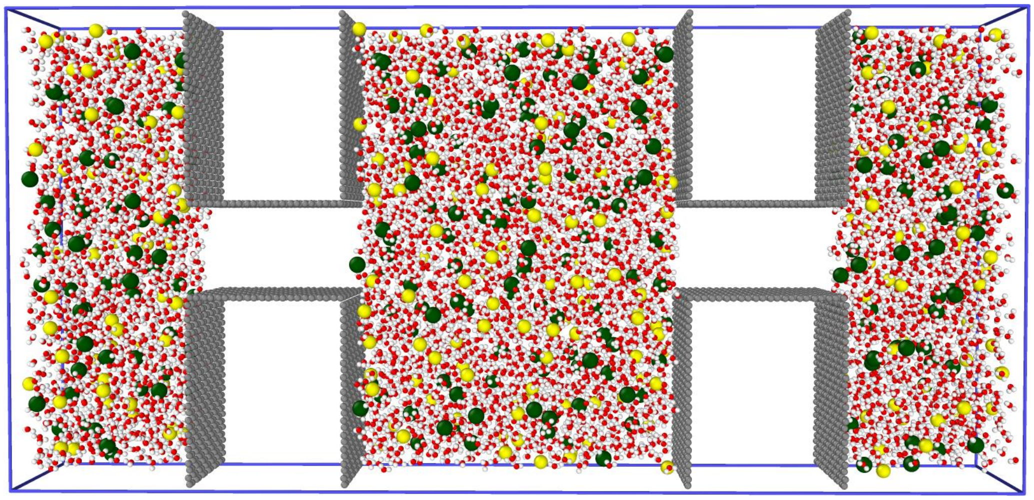

Supplementary Figure 26 │ Schematic representation of the molecular dynamics simulation systems for a confined NaCl electrolyte. Grey spheres represent graphene carbon atoms, and two face-to-face graphene sheets construct a channel. Yellow spheres represent Na^+ ions, dark green spheres represent Cl^- ions, and red and white spheres represent oxygen and hydrogen atoms of water, respectively.

Supplementary Table 1

A summary of the capacitances of state-of-the-art carbon-based electrodes

Materials	Specific capacitance ($F\ g^{-1}$)	Areal capacitance ($F\ cm^{-2}$)	Ref
Graphene/CNT film	120	-	[6]
Graphene/carbon sphere	198	-	[7]
Graphene aerogel-nanodiamond	143	-	[8]
graphene-cellulose paper	120	0.081	[9]
3D graphene-CNT sandwich	385	-	[10]
N doping CNT-graphene	180	-	[11]
CNT grown on graphene architecture	-	0.00065	[12]
Pillared graphene paper	138	-	[13]
Carbon nanoparticle electrodes	-	0.0109	[14]
Graphene-CNT composites	290.6	-	[15]
Graphene/MnO_2/CNT	372	-	[2]
Graphene/MnO_2	310	-	[16]
Graphene/MnO_2/CNT on Ni foam	251	-	[17]
MnO_2/CNT/reduce graphene oxide	193	-	[18]
CNT/GPs micro-conduits	500	2.35	This work

Supplementary Methods

Synthesis of CNT micro-conduits on carbon cloth substrates: Commercial plain carbon cloth (CC, Fuel Cell Earth LLC) was used as the substrate without further processing for CNT micro-conduit synthesis by MPCVD. Compared to conventional CVD methods, MPCVD is more efficient, less time-consuming and more controllable in designing tailored nanostructures for practical applications because of the existence of plasma[19, 20, 21]. The bioinspired leaves-on-branchlet hybrid structure with GPs grown on CNT micro-conduit was prepared through a two-step MPCVD process conducted within the same chamber that simplifies the fabrication process of the hierarchical all-carbon electrode materials. The only difference between the two-step MPCVD growth processes is the variation of growth conditions such as plasma power, growth time, and pressure. Furthermore, for industrial applications, MPCVD is also a quite promising, cost-effective method for large-scale synthesis of graphene electrode materials. CC was chosen as the substrate because of its high electrical conductivity, good mechanical flexibility and excellent electrochemical stability against acidic and alkaline electrolytes. The plasma source consists of a 2.45 GHz frequency microwave power supply with variable power. Catalyst metal was deposited on both sides of carbon cloth substrates by a Leybold e-beam evaporator at a base pressure of $5x10^{-7}$ Torr. Then the substrates were loaded on a 55-mm-diameter Mo puck in the MPCVD chamber without the insulating pillar. During the growth of CNT micro-conduits, substrates were annealed in N_2 environment while substrate temperature ramped from room temperature to 800 °C. Once the desired temperature was reached, N_2 was switched off. Control experiments to study the CNT growth without the influence of plasma were conducted with a piece of porous carbon cloth placed on the top of the sample to shield the plasma during growth.

Decorating GPs on CNT micro-conduits: For GP growth, CNT micro-conduits on CC substrates, elevated 15 mm above a 55-mm-diameter Mo puck by ceramic spacers, were inserted into the same MPCVD system. This plasma is sufficient to heat the samples from room temperature up to approx. 1100 °C, as measured by a dual-wavelength pyrometer (Williamson PRO 92). The GP growth process is catalyst-free. The mass loading of active materials (e.g., CNT, GPs) was evaluated by measuring the weight difference of a substrate before and after the MPCVD process using a microbalance with an accuracy of 1 μg. Mass measurements were carried out at least three times with different samples, and the averaged value was used as the active mass of CNT/GPs. The specific capacitance based on total mass of CNT/GP/CC is calculated to be 73.6 F g^{-1} at 1 mA cm^{-2}.

Electrodeposition of pseudocapacitive materials on CNT/GP micro-conduit nanotemplates: Electrodeposition of Ni-Co hydroxide (or PANI) on micro-conduits was conducted in a three-electrode system consisting of a CNT/GP micro-conduit as the working electrode, a Pt mesh as the counter electrode, and a saturated calomel electrode (SCE) (or Ag/AgCl) as the reference electrode. The detailed procedures have been previously described in detail elsewhere[22, 23]. Briefly, Ni-Co hydroxide was electrodeposited on electrochemically treated CNT/GP micro-conduits at a constant potential of −1.0 V versus SCE in the aqueous solution containing 0.1 M $Ni(NO_3)_2$ and 0.03M $Co(NO_3)_2$ at room temperature for 3 min. Electropolymerization of PANI was carried out on micro-conduits immersed in a solution (20 mL) containing aniline monomers (0.05 M) and H_2SO_4 (0.5 M) at 0.8 V versus Ag/AgCl for 5 min.

Supercapacitor device fabrication: Symmetric supercapacitors were assembled by sandwiching a commercial separator (Celgard™) between two CNT/GP micro-conduit electrodes, and electrochemically characterized in a two-electrode configuration cell in 1 M H_2SO_4 aqueous

electrolyte solution. The asymmetric supercapacitor was assembled by sandwiching a commercial separator (Celgard™) between the positive and negative electrodes, with a chemically treated electrode consisting of CNT/GP micro-conduits as the negative electrode and CNT/GP micro-conduits coated with Ni-Co hydroxide (electrodeposition time of 3 min) as the positive electrode.

Electrochemical tests: Cyclic voltammogram at different scan rates and constant current galvanostatic charge/discharge measurement techniques were adopted to characterize both single electrodes and supercapacitor devices for evaluation of electrochemical performance. Electrochemical impedance spectroscopy (EIS) measurements were carried out with an AC perturbation amplitude of 5 mV in the frequency ranging from 1 MHz to 0.1 Hz. The methods to calculate specific capacitances, energy and power densities are provided below.

Calculation details:

The specific capacitance of the electrodes/devices is calculated from charge/discharge curve based on:

$$C_M = (I \times \Delta t) / (\Delta V \times M) \tag{1}$$

where C_M can represent specific capacitance (F g^{-1}), areal capacitance (F cm^{-2}) or volumetric capacitance (F cm^{-3}). I (A) is the applied current, Δt (s) is the discharge time, ΔV (V) is the discharge potential range, and M can be mass, geometric area or volume of the electrodes/devices in g, cm^2 or cm^3, respectively.

Specific capacitances derived from CV tests are calculated from:

$$C_M = \frac{1}{2sM(V_h - V_l)} \oint_{V_l \to V_h \to V_l} I(V)\,\mathrm{d}V \tag{2}$$

where s is scan rate in V/s; V_h and V_l are high and low potential limits of the CV tests in V; I is the instantaneous current in CV curves; and V is the applied voltage in V.

The average energy density E (Wh kg^{-1}) and power density P (W kg^{-1}) derived from galvanostatic charge/discharge tests are calculated from:

$$E = CV^2 / 2M \tag{3}$$

$$P=E/\Delta t \tag{4}$$

Where V (V) is the applied voltage, C (F) is the capacitance of the symmetric devices, M is the total active material mass of both electrodes in symmetric devices, and Δt (s) is the discharge time.

The coulombic efficiency (η) is the ratio of the number of unit charges supplied during charging compared to the number extracted during discharging, reflecting the electrode charge storage efficiency. The coulombic efficiency measured in this study is calculated from:

$$\eta= Q_{discharge}/Q_{charge}= It_{\mathrm{discharge}}/It_{\mathrm{charge}}= t_{\mathrm{discharge}}/t_{\mathrm{charge}} \tag{5}$$

Nernst-Planck-Poisson modeling details:

Nernst-Planck-Poisson (NPP) calculations of the Gouy-Chapman model were employed to elucidate the underlying mechanisms of charge transfer and storage, as well as ion diffusion, governed by the Poisson equation

$$\nabla \cdot (\varepsilon_0 \varepsilon_r \nabla \varphi) = -\sum_i z_i e C_i \tag{6}$$

and the Nernst-Plank equation

$$\frac{\partial C_i}{\partial t} = \nabla \cdot \left[D_i \nabla C_i + \frac{D_i C_i}{k_B T} z_i e \nabla \varphi \right] \tag{7}$$

respectively, where ε_0 is the permitivity of vacuum, ε_r is the relative permittivity of the medium, φ is the elestrostatic potential, D_i is the diffusivity of chemical species i, C_i is the density of the species, z_i is the valency of the species, e is the elementary charge, k_B is Boltzmann's constant, and T is temperature.

The geometries arising from the experimental setup were used in the FE model with a petal base width of 60 nm, edge thickness of 1 nm, the gap between two petals of 30 nm, and nanotube diameter of 30 nm. The CNT/GP micro-conduit electrode was considered to be immersed in 1 M H_2SO_4 aqueous electrolyte and subjected to a voltage of 1 V. Since the electrolyte domain size is much larger than that of electrode, the eletrolyte boundary in our half model was considered as the middle of bulk electrolyte solution. Consequently, the potential boundary conditions for Poisson's equation were specified as 0.5 V for the positive electrode and 0 V for the electrolyte boundary due to symmetry. The boundary conditions for the time-dependent Nernst-Plank equation were specified as the eletrolyte boundary ionic concentration of 1 M SO_4^{2-} and 2 M H^+. All other boundaries were specified as zero flux. Areal capacitance from the non-uniform charge distribution is calculated as

$$C_A = \frac{\int A(\mathbf{x})\rho^s(\mathbf{x})dA}{\int A(\mathbf{x})dA \times V} \tag{8}$$

where ρ^s and A are the surface charge density and occupied area corresponding to the point of interest **x,** respectively. V is the applied voltage.

Since the electrolyte domain size is much larger than that of electrode, the eletrolyte boundary in our half model was considered as the middle of bulk electrolyte solution. Therefore, the boundary conditions for Eq. 7 were specified as the eletrolyte boundary ionic concentration of 1

M SO_4^{2-} and 2 M H^+. All other boundaries were specified as zero flux. The required material properties were specified as the relative permittivity of electrolyte: 3, CNT: 15 and GPs: 81. The diffusion coefficients were set as H^+: 6.5x10^5 cm^2 s^{-1} and SO_4^{2-}: 2x10^5 cm^2 s^{-1}, according to Ref. [24]. To establish grid independence, the system mesh was set as 333816 tetrahedral elements with a smallest element size of 0.2 nm based on solution convergence trials.

Molecular Dynamics simulation details:

Molecular dynamics (MD) simulations were performed to calculate the size effect of the ion diffusion coefficients in the confined graphene nanochannels. Fig. S26 shows a schematic representation of the simulation model of confined eletrolyte NaCl. The simulations were performed in a cubic box at room temperature. The pressure for bulk systems is 300 ±150 atmospheres, similar to in the literature.[25] The results show that the behavior of ions diffusion is almost unchanged once the size of confined nanochannels exceed 5 nm. According to Ref. [26], the effective ionic diameters of NaCl eletrolyte and H_2SO_4 electrolyte are basically comparable, as 0.19–0.76 nm for Na^+, 0.36–0.66 nm for Cl^-, 0.22–0.56 nm for H^+ and 0.58-0.76 nm for SO_4^{2-}. Thus, such a over 5 nm size-indepence bahavior is fairly spectulated for the H_2SO_4 electrolyte. Based on statistically calculation of experimental results indicates the gap between two graphene petals are about 10–40 nm. In this regard, the size effect of confined nanochannels is reasonably ignored.

References

1. He, Y. *et al.* Freestanding three-dimensional graphene/MnO_2 composite networks as ultralight and flexible supercapacitor electrodes. *ACS nano* **7**, 174–182 (2012).

2. Cheng, Y., Lu, S., Zhang, H., Varanasi, CV. & Liu, J. Synergistic effects from graphene and carbon nanotubes enable flexible and robust electrodes for high-performance supercapacitors. *Nano Lett.* **12**, 4206–4211 (2012).

3. Choi, BG., Yang, M., Hong, WH., Choi, JW. & Huh, YS. 3D macroporous graphene frameworks for supercapacitors with high energy and power densities. *ACS nano* **6**, 4020–4028 (2012).

4. Zhou, W. *et al.* Flexible wire-like all-carbon supercapacitors based on porous core–shell carbon fibers. *J. Mater. Chem. A* **2**, 7250–7255 (2014).

5. Wu, Z-S. *et al.* High-energy MnO2 nanowire/graphene and graphene asymmetric electrochemical capacitors. *ACS nano* **4**, 5835–5842 (2010).

6. Yu, D. & Dai, L. Self-assembled graphene/carbon nanotube hybrid films for supercapacitors. *J. Phys. Chem. Lett.* **1**, 467–470 (2009).

7. Guo, CX. & Li, CM. A self-assembled hierarchical nanostructure comprising carbon spheres and graphene nanosheets for enhanced supercapacitor performance. *Energy Environ. Sci.* **4**, 4504–4507 (2011).

8. Wu, Q., Sun, Y., Bai, H. & Shi, G. High-performance supercapacitor electrodes based on graphene hydrogels modified with 2-aminoanthraquinone moieties. *Phys. Chem. Chem. Phys.* **13**, 11193–11198 (2011).

9. Weng, Z. *et al.* Graphene–cellulose paper flexible supercapacitors. *Adv. Energy Mater.* **1**, 917–922 (2011).

10. Fan, Z. *et al.* A three-dimensional carbon nanotube/graphene sandwich and its application as electrode in supercapacitors. *Adv. Mater.* **22**, 3723–3728 (2010).

11. You, B., Wang, L., Yao, L. & Yang, J. Three dimensional N-doped graphene–CNT networks for supercapacitor. *Chem. Commun.* **49**, 5016–5018 (2013).

12. Kim, Y-S., Kumar, K., Fisher, FT. & Yang, E-H. Out-of-plane growth of CNTs on graphene for supercapacitor applications. *Nanotech.* **23**, 015301 (2011).

13. Wang, G. *et al.* Flexible Pillared Graphene-Paper Electrodes for High-Performance Electrochemical Supercapacitors. *Small* **8**, 452–459 (2012).

14. Yuan, L. *et al.* Flexible solid-state supercapacitors based on carbon nanoparticles/MnO2 nanorods hybrid structure. *ACS nano* **6**, 656–661 (2011).

15. Cheng, Q. *et al.* Graphene and carbon nanotube composite electrodes for supercapacitors with ultra-high energy density. *Phys. Chem. Chem. Phys.* **13**, 17615–17624 (2011).

16. Yan, J. *et al.* Fast and reversible surface redox reaction of graphene–MnO 2 composites as supercapacitor electrodes. *Carbon* **48**, 3825–3833 (2010).

17. Zhu, G. *et al.* Highly conductive three-dimensional MnO 2–carbon nanotube–graphene–Ni hybrid foam as a binder-free supercapacitor electrode. *Nanoscale* **6**, 1079–1085 (2014).

18. Lei, Z., Shi, F. & Lu, L. Incorporation of MnO2-coated carbon nanotubes between graphene sheets as supercapacitor electrode. *ACS Appl. Mater. Inter.* **4**, 1058–1064 (2012).

19. Meyyappan, M. A Review Of Plasma Enhanced Chemical Vapour Deposition Of Carbon Nanotubes. *J. Phys. D Appl.Phys.* **42**, 103–109 (2009).

20. Kobashi, K., Nishimura, K., Kawate, Y. & Horiuchi, T. Synthesis of diamonds by use of microwave plasma chemical-vapor deposition: Morphology and growth of diamond films. *Phys. Rev. B Condensed Matter* **38**, 4067–4674 (1988).

21. Bo, Z. *et al.* Plasma-enhanced chemical vapor deposition synthesis of vertically oriented graphene nanosheets. *Nanoscale* **5**, 5180–5204 (2013).

22. Xiong, G., Meng, C., Reifenberger, RG., Irazoqui, PP. & Fisher, TS. Graphitic Petal Electrodes for All-Solid-State Flexible Supercapacitors. *Adv. Energy Mater.* **4**, 1300515: 1–9 (2014).

23. Xiong, G. *et al.* Hierarchical Ni–Co Hydroxide Petals on Mechanically Robust Graphene Petal Foam for High-Energy Asymmetric Supercapacitors. *Adv. Funct. Mater.* **26**, 5460–5470 (2016).

24. Umino, S. & Newman, J. Diffusion of sulfuric acid in concentrated solutions. *J. Electrochem. Soc.* **140**, 2217–2221 (1993).

25. Kong, J. *et al.* Temperature dependence of ion diffusion coefficients in NaCl electrolyte confined within graphene nanochannels. *Phys. Chem. Chem. Phys.* **19**, 7678–7685 (2017).

26. Pilon, L., Wang, H. & D'Entremont A. Recent Advances in Continuum Modeling of Interfacial and Transport Phenomena in Electric Double Layer Capacitors. *J. Electrochem. Soc.* **162**, A5158–A5178 (2015).
