# Supplementary material for: Bioinspired leaves-on-branchlet hybrid carbon nanostructure for supercapacitors
Source: Nat Commun. 2018 Feb 23;9:790. doi: 10.1038/s41467-018-03112-3 (PMC5824788; doi:10.1038/s41467-018-03112-3)
Supplement: Supplementary file 2 — Peer Review File [file 41467_2018_3112_MOESM2_ESM.pdf]

Reviewers' comments:

Reviewer #1 (NKT, CD1d, Breg)(Remarks to the Author):

This manuscript has investigated interactions between CD1d-expresssing regulatory B cells and iNKT cells. Using a rheumatoid arthritis model in mice, the authors show that CD1d-mediated antigen presentation by Bregs is critical for iNKT cell function. These interactions were required for the suppressive effects of iNKT cell activation on arthritis. These effects were independent of IL-10 production by Breg cells. From these findings, the authors conclude a critical role of CD1d on regulatory B cells for regulating iNKT cell function, in a manner that is independent of IL-10 production by B cells.

General comments:

The manuscript includes an impressive amount of data, with a variety of complementary approaches such as cell depletion, knockout mice, mixed bone marrow chimeras, adoptive transfers, etc. The data are convincing, well-described, and potentially important for understanding interactions between regulatory B cells and iNKT cells during autoimmunity and inflammatory responses.

Minor comments:

1. Mizoguchi et al. (Immunity 2002; 16:219-230) were the first to provide evidence for a role of CD1d on B cells in controlling inflammatory responses in a colitis model. In this system, CD1d and IL-10 were important, but not NKT cells. This report should be included and differences with the present work may be briefly discussed. For example, in some cases signaling through CD1d (e.g., via antibody stimulation) has been shown to influence functions of CD1d-expressing cells, providing one potential explanation for CD1d-dependent but NKT cell-independent effects (I believe the Mizoguchi paper largely used B cells purified based on CD1d expression, presumably using mAbs that could have potentially activated the B cells).

2. A variety of prior studies have studied the role of iNKT cells and iNKT cell activation on different models of rheumatoid arthritis. This includes Coppieters et al. (J. Immunol. 2007; 179:2300-2309); Horikoshi et al. (PLoSOne 2012; e51215); Miellot et al. (EJI 2005; 35:3704-3713), and several others. Although the model employed here is different than most of the models employed in these prior studies, these studies should be briefly discussed, because the studies reported here might not apply to other experimental arthritis models. Ideally, the key finding would be confirmed in a separate arthritis model, but considering the extensive amount of data already included in the manuscript, a balanced discussion of that particular limitation of the study appears sufficient.

Reviewer #2 (Breg, B/T interaction)(Remarks to the Author):

This study by Oleinika and colleagues describe a role for CD1d on T2-MZP in suppressing arthritis in mice via their interaction with iNKT cells in an IL-10-independent manner, which is supported by the data shown. Less convincing is the mechanism whereby the suppression occurs. The data shown suggesting that the mechanism is by induction of IFN-g production of iNKT cells and suppression of CD4 T cell differentiation is less convincing. The manuscript is well written and provides an explanation for why B cells that express high levels of CD1d exhibit regulatory function. This result will be of interest to the field.

Specific Comments
1. Figure 3H. The purpose of the arrowheads should be stated in the figure legend.

2. Fig. 5B-C. While the data may be significant the changes observed are so small that they are not likely biologically relevant.

3. Figure 5D. In this figure it is claimed that differentiation of CD4+ T cells is examined. In the results it is stated that the T cells come from mice with arthritis, yet in the figure legend this is not stated. Thus the source of the T cells is not clear. If the T cells were from mice with arthritis then they would have already encountered signals driving their differentiation. The assay as designed does not actually determine whether T cells differentiated. What it shows is whether uMT iNKT cells can promote the production of IFN-g in cD4 T cells of an unknown activation/differentiated status. Thus the data are of little value. In addition, the change in the percentage of IFN-g+ cells is so small that it is not likely biologically relevant.

4. Fig. 5E. This figure does not show that IFN-g inhibited the differentiation of Th1 and Th17 cells. What it shows is that IFN-g inhibited T-bet and ROR-gt expression. This is not the same thing as preventing differentiation. Again the changes are so small that it is not clear if they are biologically relevant.

5. The studies in Fig. 5 do not directly demonstrate that Breg induced IFN-g production by iNKT cells. Thus the claims made in this figure are not supported by the data shown.

6. Figure 8D. The B cells utilized in this assay are not the same as those being examined throughout the rest of the study. Thus the experimental design fails to determine whether iNKT cells are required for the development of T2-MZP that can suppress via interaction with iNKT cells via CD1d. Since the mechanism is IL-10-independent it is not clear why IL-10 producing B cells were examined. It is not clear whether IL-10-producing B cells that are not CD1d+T2-MZP are required for suppression in the studies shown.

7. Line 328. B10 are not a definitive Breg subset as suggested. They are simply IL-10 producing B cells.

Minor comments

8. Some of the journal names in the reference list are not abbreviated.

9. Isolation of liver cells is described in the Materials and Methods, but it is not clear where these cells were used in the figures.

Reviewer #3 (Breg, tolerance)(Remarks to the Author):

This paper aims to characterise a novel, IL-10-independent mechanism by which CD1d+ B cells present lipid to iNKT cells which in turn, through IFNg release, inhibit Th1 and Th17 immune responses in a mouse model of arthritis.

The authors make excellent use of chimeric mouse models to address their hypotheses. This is a clearly written, detailed study.

There are issues which should be addressed to clarify and confirm some of the authors' claims. Some of the experiments have only been repeated once.

1a. If Breg are important in this arthritis mouse model, one would expect to see an exacerbation of arthritis in the uMT mouse compared to the WT, regardless of a-GalCer. Can the authors explain why this does not appear to be the case?

1b. It would be relevant here to show the iNKT levels within the draining lymph node, as well as the spleen.
To claim that there is an equal increase in iNKT levels of uMT and WT mice, this increase between the two models needs to be compared in the statistical analysis. Currently, only the increase in iNKT when a-GalCer is added within each model, is shown.

1e. A substantial proportion of iNKT cells still express IFNg in the uMT model – what about other cytokines, such as IL-4 and IL-10 etc?

1g. The authors should add a label to the bar chart indicating no significance between WT and uMT liver IFNg+iNKT when a-GalCer is added.

2c. To claim that there is an equal increase in iNKT levels of WT and Rituximab-treated mice, this increase between the two conditions needs to be compared in the statistical analysis. Currently, only the increase in iNKT when a-GalCer is added within each model, is shown.

S1a-d. There is a substantial decrease in iNKT cells to negligible levels, when DCs are depleted and a-GalCer is added. Can the authors explain how such a decrease in iNKT levels still does not affect the amelioration of arthritis when a-GalCer is added upon DC-depletion? One would expect some clinical difference, given the complete lack of iNKT cells upon a-GalCer administration in this model, as this would surely translate to negligible levels of iNKT cells expressing IFNg?

Fig. S2. CD1d expression within the IL-10 transgenic mice should be demonstrated.

Fig. 4d. Can this increase in gene expression of IL-10, IL-21 etc be detected as proteins i.e. ELISA/Facs, to support functional relevance?

Fig. 5a-b. When anti-IFNg is added to WT with vehicle (positive control), it appears that inflammation and IFNg+CD4+ T cell levels are reduced compared to WT with vehicle and no anti-IFNg (negative control), indicating that the anti-IFNg itself is having an effect on inflammation. Thus it is not possible to deduce that the increased inflammation upon a-GalCer therapy in the presence of anti-IFNg is directly due to blockade of IFNg normally elicited by a-GalCer. i.e. The effect on IFNg expression by T cells may be due to the fact that IFNg has been blocked by the inhibitor, rather than the action of a-GalCer being blocked by the inhibitor.

The bar chart in Fig. 5a also does not represent what is described in the legend. In Fig. 5b-c, representative dot plots should be provided, particularly given such low percentages.

Fig. 5c. It is not clear what the statistical bars on the graph are comparing. Why is there such a large relative increase in ROR-gt+ T cells by uMT mice when a-GalCer is added? What non-B cells express CD1d in this model?
Fig. 5d. To directly show that iNKT cells can inhibit IFNg expression in CD4+ T cells via an IFNg-dependent mechanism, the authors should isolate iNKT cells from WT mice treated with anti-IFNg and culture these IFNg blocked iNKT cells with T cells from arthritic WT mice.

Fig. 5e. Given that % levels have been demonstrated throughout the manuscript, it is not clear why the authors choose to illustrate MFI. % levels should be included for consistency.

S1f. Given that this model is set up differently to the model described in Fig 1-2, is there an overall difference in levels of IFNg+iNKT cells between the uMT model and DC-depletion model?

S4c. The x axis on the second facs plot showing pre-sort strategy should read, I assume,'CD23,' not 'CD24.' Whilst Facs staining is shown, purities are not actually stated, this should be amended.

Fig 6. Whilst this figure is interesting and suggests iNKT-dependency of T2MZP Breg, some clarity is required. Given that T2MZP Breg-mediated suppression has been previously shown to be IL-10-dependent in similar models (by the same group), they should demonstrate the IL-10 expression by T2MZP Breg in both WT and CD1d-/- mice. Is there any difference in IL-10 expression to account for the different clinical outcomes? Also, when T2MZP Breg are transferred into WT mice, is there a decrease in IFNg expression by iNKT cells but maintenance of frequency, as described earlier in the paper? This needs to be established before extrapolating that T2MZP Breg represent the earlier described CD1d+ Breg.

Fig 6b, 6d, 6e. The statistics on the bar charts are poorly labelled and it is unclear why different experimental groups are being compared upon different charts. On some graphs, the statistics are completely absent, despite comments within the text.

Fig. 7b-e. The text states that the levels of iNKT cells Ki-67, PLZF and IFNγ+iNKT cells are comparable between control and MZ B cell-depleted mice. The statistics on the graphs compare a-GalCer treatment to vehicle only, and do not compare the levels between the control and experimental groups. This should be amended.

Given that MZ B cells express significantly higher levels of CD1d than any other splenic B cell subset (as demonstrated in the supplementary data), and the authors claim that suppression is mediated by CD1d expressing B cells, why cannot MZ B cells suppress inflammatory responses similar to T2MZP Breg or CD1d+ B cells? The suggestion that MZ B cells may not come in contact with iNKT cells in Fig. 3h, is associative at best.

Fig. 8a-c. Representative Facs plots of IL-10 staining should be shown.

Given that a-GalCer presentation by B cells to iNKT cells is known to result in differentiation of ab-producing B cells, the authors should look to see whether a) this is the case in this model, b) ab-producing B cells/plasma cells are subsequently regulatory and express IL-35.

7/23/2017

Dear Editor:

On behalf of my co-authors, I write to resubmit the enclosed manuscript entitled "Bioinspired Leaves-on-branchlet Hybrid Carbon Nanostructure for Supercapacitors" for consideration of publication in *Nature Communications*. Please note that a previous version of this paper was submitted to *Nature Communications* (NCOMMS-16-18426) and reviewed in October 2016. The related reviews identified some areas for further in-depth study and a series of additional experiments, which I am now pleased to report are complete.

The reviews of the original manuscript (NCOMMS-16-18426) were quite valuable to us in expanding our work. I note that, in spite of a consistent desire for more testing (for details see the response to the reviewers), the reviews were highly complementary of our work, particularly of the novelty with respect to materials. Two of the reviewers suggested that **it should be considered for publication in *Nature Communications*** and the other one indicated it could be published after addressing the issues raised in the comments (emphasis **added**):

• Review 1: "This manuscript presents a micro-conduit in a bioinspired leaves-on-branchlet structure consisting of carbon nanotube (CNT) arrays serving as branchlets and graphene petals (GPs) as leaves for supercapacitor electrodes. The idea is **original**, which is inspired by the phenomenon in nature that leaves significantly increase exposed tree surface area to absorb CO2 (like ions) from the environments (like electrolyte) for photosynthesis. The as-obtained supercapacitors exhibit high specific capacitance, high rate performance, and high stability. Theoretical calculations were conducted to elucidate the underlying mechanism of charge transfer and storage governed by sharp GP edges. This manuscript was **well written** and **organized**, and **the originality and broad impact are obvious**. **The referee recommends its publication in Nature Communications. Minor comments.**"

• Review 2: "The authors report on the fabrication of micro-conduits based on carbon nanotube/graphene petal (CNT/GP) architecture that show **significant improvement** on both **mechanical and electrochemical properties** compared to CNT-based micro-conduits. It is **impressive** that CNT/GP electrodes yield (1) much higher current density than other nano-structured carbon electrodes and (2) considerably better (specific) capacitance than existing all-carbon electrodes in supercapacitor applications. The authors also demonstrated that the CNT/GP architecture can be used as a nanotemplate for pseudocapacitive materials. As such**, I think that this work merits publication in Nature Communication.**"

• Review 3: "This manuscript reports **a new type of interesting** carbon nanotube (CNT) arrays/graphene petals (GPs) core-branch all-carbon micro-conduit electrode for supercapacitors."

We completely agree with the thorough and constructively critical review comments, and to resolve these concerns comprehensively we have conducted extensive additional experiments and provided relevant analysis in the revised manuscript.

Moreover, the main purpose of this letter is to address the concerns from the third review. Again, we deeply appreciate the third review's constructive suggestions. However, we respectfully disagree with the notion regarding **the focus of this work**. Although MPCVD has been reported in prior literature as an effective means to synthesize GPs on substrates including carbon cloth (CC), and our previous work has reported such an electrode structure, the purpose (or the theme) of this work is ***NOT*** to emphasize the GP growth itself nor MPCVD methods, but instead *to solve the main issue that hinders the development of highly ordered carbon electrodes, particularly CNT arrays, with commonly observed problems such as easy destruction of the tube orientation and poor mechanical robustness and electrochemical performance. The all-carbon hybrid CNT/GP micro-conduits reported here have been, for the first time, systematically and controllably synthesized and applied to electrochemical energy storage.* We apologize for some of the unclear/ambiguous sentences in the abstract and introduction that may have caused any confusion. However, we modestly emphasize the uniqueness and novelty of the micro-conduits in a leaves-on-branchlet nanostructure to clarify any such confusion and to enumerate specifically the reasons that this submission is a major step forward in comparison to prior work:

1) This manuscript mainly focuses on two aspects: a) how to maintain the orientation of the highly ordered CNT arrays, namely mechanical robustness; and b) how to design 3D electrodes based on CNT arrays to enhance overall electrochemical performance. In other words, CNT arrays designed in a shape of micro-conduits as an efficient scaffold synergistically strengthened and enhanced by GPs are far more important than GP growth itself or the MPCVD method (We note that review 3 also took a tremendous amount of the interest in this structure judging from the detailed questions raised concerning CNT micro-conduits). To solve these two issues, inspired by the common phenomenon in nature, we adopted GPs as an effective the solution to successfully achieve outstanding mechanical robustness and electrochemical performance by decorating GPs on CNT micro-conduit walls in a leaves-on-branchlet nanostructure. The substantial differences in the novelty of this work from earlier work are highlighted for a clear comparison:

— *Completely distinct design concept of the CNT/GP micro-conduits as compared to earlier work*: The heart-shape CNT/GP micro-conduits prove more than "eye-catching" (we of course do not apologize for their aesthetic appeal, which is also a feature of the natural world) but also practically favorable in terms of mechanical robustness, ion transfer kinetics and electrochemical performance: (i) Design concept difference from 2D GP active materials (approx. 500-nm-thick) to 3D CNT/GP micro-conduits (with CNT array length in the range of 100 microns in the micro-conduit wall) on carbon fibers (the differences of the two structures are shown in Fig. 1). Because the major issue facing today's supercapacitors is limited energy density, the design of lightweight 3D graphene-based electrode materials is crucial. (ii) High structural orientation in CNT array micro-conduits facilitates ionic charge transfer and results in good electrochemical performance, and GP decoration on the micro-conduits achieves this goal by mechanically and electrochemically strengthening the scaffolds. (iii) Hollow channels within the micro-conduits over an efficient scaffold with open channels facilitating ionic penetration and

transfer within the 3D micro-conduit electrodes, leading to low internal resistance and high rate capability while maintaining at high capacitance.

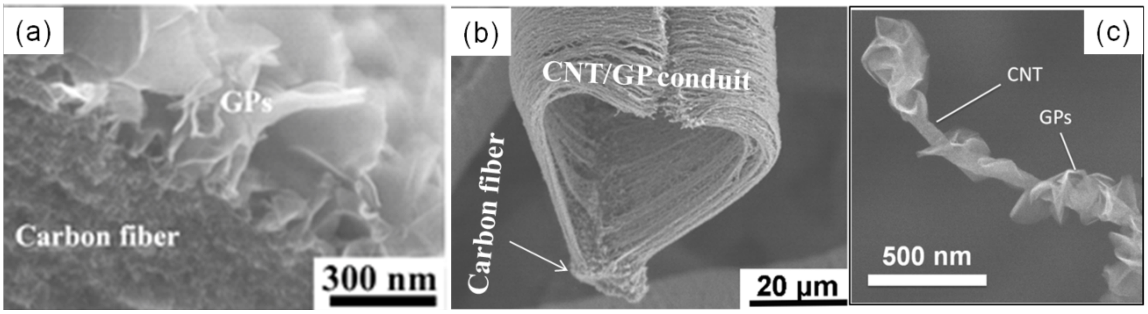

Fig. 1. Structural difference between (a) 2D GP layers and (b) 3D micro-conduits on a carbon fiber. (c) Magnified SEM image of the leaves-on-branchlet structure in (b).

— *Significantly superior electrochemical performance of CNT/GP micro-conduits was observed as compared to earlier work*. Because of the foregoing structural advantages of CNT micro-conduits as a 3D scaffold and the synergic role of CNT/GPs in a leaves-on-branchlet structure, the areal capacitance of the 3D CNT/GP micro-conduit electrodes exhibit approx. 3 times higher capacitance than earlier work (see Fig. 2).

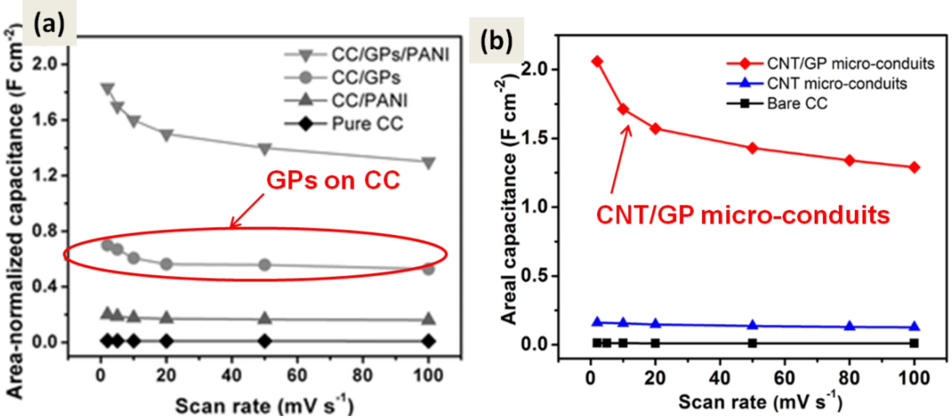

Fig. 2. Comparison of electrochemical performance of this work and earlier work. (a) Earlier work shows that GPs on CC exhibit an areal capacitance of approx. 0.7 F cm^{-2} at a scan rate of 2 mV s^{-1}. (b) This work shows that CNT/GP micro-conduit electrodes exhibited approx. 2.1 F cm^{-2}, which is about 3 times as high as that of earlier work at the same scan rate.

— *Substantial advantages of CNT/GP array* ***micro-conduits*** *over conventional arrays without micro-conduits*. Micro-conduits with hollow channels facilitate favorable GP decoration on CNT arrays in the conduit walls (see Fig. 3). Hollow channels within the micro-conduit and uniform GP decoration results in approximately 10-fold improvement in area capacitance (> 2 F cm^{-2}) as compared to that (< 0.2 F cm^{-2}) of arrays without micro-conduits on flat Si substrates because of the non-uniform GP decoration on

conventional CNT arrays on flat substrates with highly non-uniform petal density because of poor coupling between plasma and inner CNTs. This comparison between the cases with/without micro-conduits clearly demonstrates the uniqueness of the design of the micro-conduits and hierarchal CNT/GPs with a leaves-on-branchlet structure.

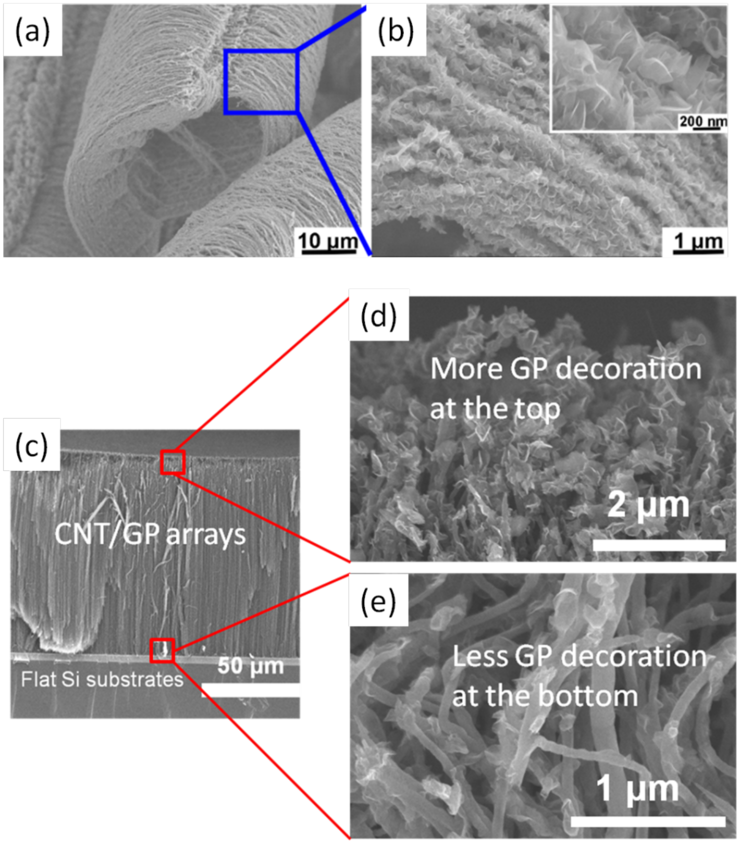

Fig. 3. (a, b) CNT micro-conduits facilitate uniform GP growth on entire CNT arrays. (c, d, e) Non-uniform GP coverage within the CNT arrays because of the poor coupling between plasma and inner CNTs. The excessive GP coverage on the top blocks the pores of the hybrid structure, resulting in higher resistance during ion penetration process and thus deteriorating the electrochemical performance.

2) Whether the "electrode structure with a core-branch architecture is not a new concept"? Although core-shell (or branch) structures consisting of metal oxides/sulfide have been reported in prior work (see refs. *Nature Communications* 2015, 6, 6694; *Nature Communications* 2011, 2, 381; *ACS Nano* 2012, 6, 5531–5538; *Advanced Materials* 2011, 23, 2076–2081; Nano Letters 2011, 11, 1215–1220; *Advanced Materials* 2012, 24, 938–944), a leaves-on-branchlet nanostructure consisting of all-carbon CNT/GP directly grown by MPCVD without any binder has, to the best of our knowledge, ***never*** been employed as electrode materials in electrochemical energy storage. These hierarchical, lightweight, all-carbon nanostructures with a controllable and systematic growth prove to be high-performance active materials and efficient nanotemplates for

highly-pseudocapacitive materials. This work therefore provides a first milestone toward the fabrication of high-performance, binder-free, flexible, lightweight and scalable power supplies for electronic devices.

3) We also clarify that our objective is to design a bioinspired leaves-on-branchlet hybrid carbon nanostructure, and our Nernst-Planck-Poisson calculations thus aim to elucidate the underlying mechanisms of charge transfer and storage, as well as ion diffusion for such a structure based on experimentally measured and/or previously reported materials (e.g., graphene) and their structural geometries. To the best of our knowledge, cited paper in Journal of Power Source reports a molecular dynamics simulation of only a simplified vertically oriented single-layer graphene array, and it is difficult to extend the model to the proposed leaves-on-branchlet structure because of the high computational cost of this complex geometry. We also emphasize that the computational model is applicable to different materials and is expected to inspire new material combinations for this specific structure.

Given the clarifications above and the positive comments from reviewers, we request your re-consideration of our manuscript's suitability for the journal. The development of electrode materials with innovative, new structures for energy storage has been a research topic frequently highlighted in *Nature Communications* and continues to receive extensive attention. We believe this manuscript including both new ***experimental*** and ***simulation*** elements will elicit much interest from the community and will attract broad attention from the journal's readership.

The previous version of this paper was submitted to *Nature Communications* (NCOMMS-16-18426) and in what follows, we provide a response to each point raised and outline the changes made to the revised manuscript:

Author response to Review No. 1

Bioinspired Leaves-on-branchlet Hybrid Carbon Nanostructure for Supercapacitors
by
Guoping Xiong, Pingge He, Zhipeng Lyu , Tengfei Chen, Boyun Huang, Lei Chen, Timothy S. Fisher

We thank the reviewer for careful reading of the manuscript. In what follows, we provide a response to each point raised and outline the changes made (in blue font below) to the revised manuscript. For convenience, the various cited references in the response are provided at the end of this document.

"This manuscript presents a micro-conduit in a bioinspired leaves-on-branchlet structure consisting of carbon nanotube (CNT) arrays serving as branchlets and graphene petals (GPs) as leaves for supercapacitor electrodes. The idea is original, which is inspired by the phenomenon in nature that leaves significantly increase exposed tree surface area to absorb CO2 (like ions) from the environments (like electrolyte) for photosynthesis. The as-obtained supercapacitors exhibit high specific capacitance, high rate performance, and high stability. Theoretical calculations were conducted to elucidate the underlying mechanism of charge transfer and storage governed by sharp GP edges. This manuscript

well written and organized, and the originality and broad impact are obvious. The referee recommends its publication in Nature Communications. Minor comments:"

Comment 1. Please give more discussion on the mechanical stability of the hybrid structure.

Authors' Response: We appreciate this comment. The mechanical stability of the hybrid structure is crucial to the electrochemical performance of such electrode materials. To further demonstrate the outstanding mechanical performance of the hybrid structure, CNT/GPs micro-conduits were ultrasonically treated in a water bath through an ultrasonic cleaner instrument for 30 mins. As shown in Supplementary Figure 14, after 30 mins, typical micro-conduit channels still remain, with intact leaves-on-branchlet structure after strongly ultrasonic treatment. From the high-magnification SEM image in Figure 14d, GPs were still uniformly and densely coated on CNT arrays, demonstrating the high mechanical robustness of the CNT/GP micro-conduit structure. Furthermore, the CNT/GP micro-conduit electrode exhibits approx. 95% capacitance retention over 10,000 charge/discharge cycles and high coulombic efficiencies close to 100%, indicating excellent long-term cyclic stability, which is attributed to the outstanding structural and mechanical stability of the hybrid electrode.

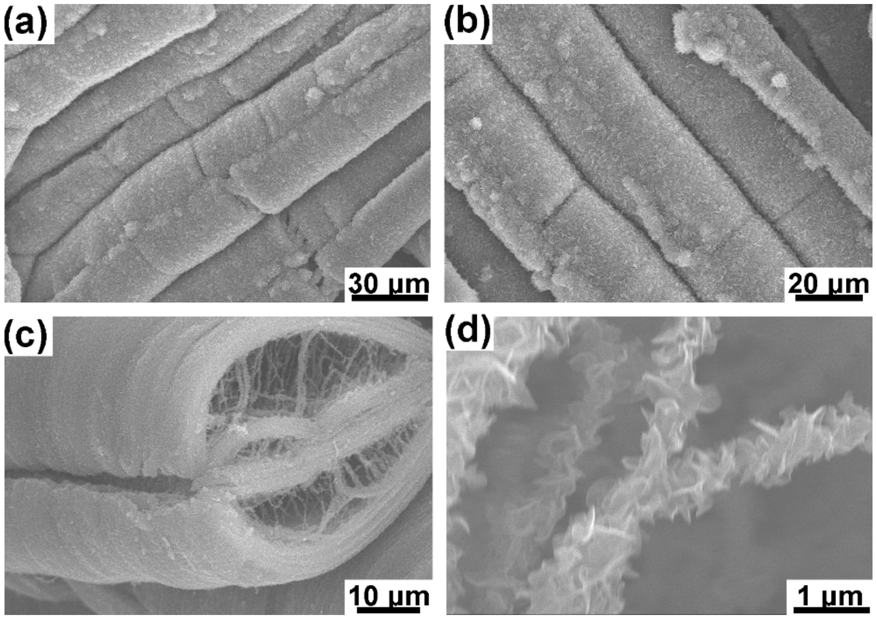

Supplementary Figure 14 | SEM images of the CNT/GP micro-conduit structure after ultrasonic treatment for 30 mins. (a, b) Top views of treated CNT/GP micro-conduits. (c) A typical treated CNT/GP micro-conduit structure in a heart shape. (d) Leaves-on-branchlet structure at high magnification after treatment.

Changes to Manuscript: According to this comment, we have made the following changes on **page 11** of the modified manuscript:

"Moreover, the CNT/GP micro-conduit electrodes were ultrasonically treated in a water bath for 30 mins and the results are provided in Supplementary Fig. 14, further revealing the high mechanical robustness of CNT/GP micro-conduit structures."

Moreover, the SEM images of the CNT/GP micro-conduit electrodes after ultrasonic treatment have been added in Supplementary Figure 14:

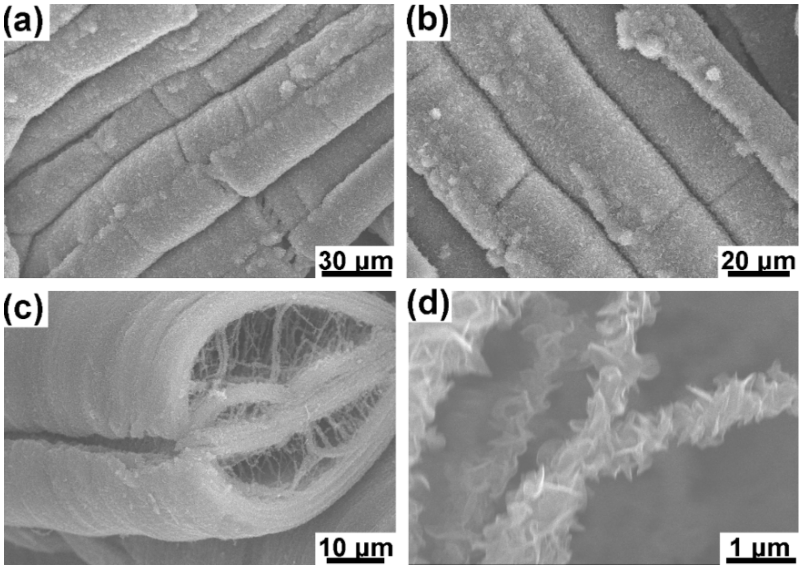

"**Supplementary Figure 14 | SEM images of the CNT/GP micro-conduit structure after ultrasonic treatment for 30 mins.** (a, b) Top views of treated CNT/GP micro-conduits. (c) A typical treated CNT/GP micro-conduit structure in a heart shape. (d) Leaves-on-branchlet structure at high magnification after treatment."

Comment 2. The authors mentioned the roles of the sharp edges of GPs on the capacitive behavior. It is correct. Please give more discussion on the possible influences of the edge density.

Authors' Response: We appreciate this comment. The edge density of GPs has a close relationship to the capacitive behavior of electrodes. As demonstrated in Supplementary Figure 9, the areal capacitances of electrode vary with different GP growth durations (18, 25 and 35 min). As the growth duration increases from 18 min to 35 min, more GPs grow on the micro-conduit surface, leading to higher edge density of GPs. As shown in Supplementary Figure 9, at low scan rates, the areal capacitance of the micro-conduit electrode increases with the longer growth duration, which indicates that increasing edge density facilitates better capacitive performance, consistent with the findings reported in ref. [1]. However, at high scan rates, the electrodes with higher edge density exhibit lower capacitances. Increasing edge density could significantly improves the surface area, but in the meanwhile it might block pores, which inhibits the ion transfer and thus leading to

poor electrochemical performance. The results indicate that an optimal edge density of GPs exists for superior electrochemical performance because of a balance between surface area and ion transfer kinetics.

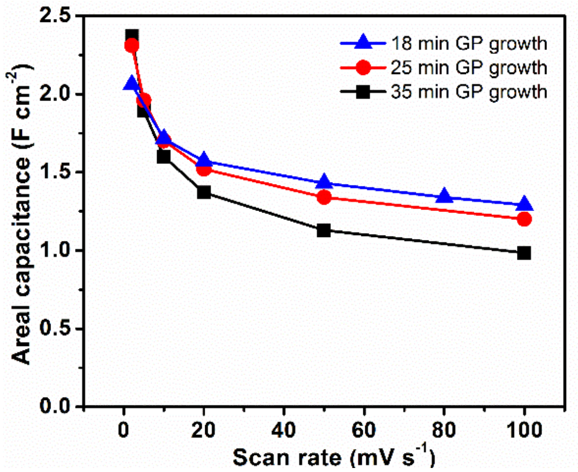

Supplementary Figure 9 | Areal capacitances of electrodes with different GP growth durations (18, 25 and 35 min) as functions of scan rates.

Changes to Manuscript: According to this comment, we have made the following changes on **page 9** of the modified manuscript:

"The areal capacitances of CNT/GP micro-conduit electrodes with different GP growth durations have been plotted as a function of scan rates in Supplementary Fig. 9. The results reveal a relationship between the edge density of GP and capacitance of CNT/GP micro-conduit electrodes, and indicate that an optimal GP growth time exists for superior electrochemical performance because of a balance between surface area and ion transfer kinetics."

Moreover, following changes are made on **page 9** of the modified Supplementary:

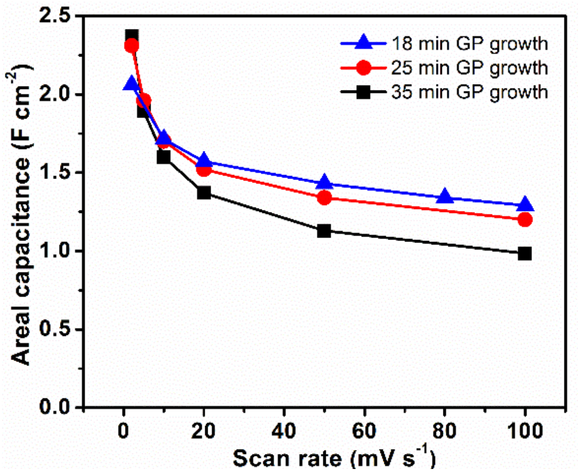

"**Supplementary Figure 9 |** Areal capacitances of electrodes with different GP growth durations (18, 25 and 35 min) as functions of scan rates. The results reveal a relationship

between the edge density of GP and capacitance of CNT/GP micro-conduit electrodes, and indicate that an optimal GP growth time exists for superior electrochemical performance because of a balance between surface area and ion transfer kinetics"

Comment 3. The introduction of GPs increased the energy storage ability. Will it also increase the internal resistance?

Authors' Response: We appreciate this comment. We further measured and compared the internal resistances of CNT micro-conduit and CNT/GP micro-conduit electrodes. The comparative Nyquist plots of CNT micro-conduit and CNT/GP micro-conduit electrodes are now included as Supplementary Figure 10. As shown in Supplementary Figure 10, after the introduction of GPs, the R_e increases slightly from 1.85 to 2.8 Ω, since dense GPs grown on CNT micro-conduits would block some pores, and in the meanwhile inhibit ions transfer, consequently increasing the internal resistance. Moreover, negligible semicircles in the high frequency region from impedance spectra of both electrodes indicates low charge transfer resistances of CNT and CNT/GP micro-conduit electrodes.

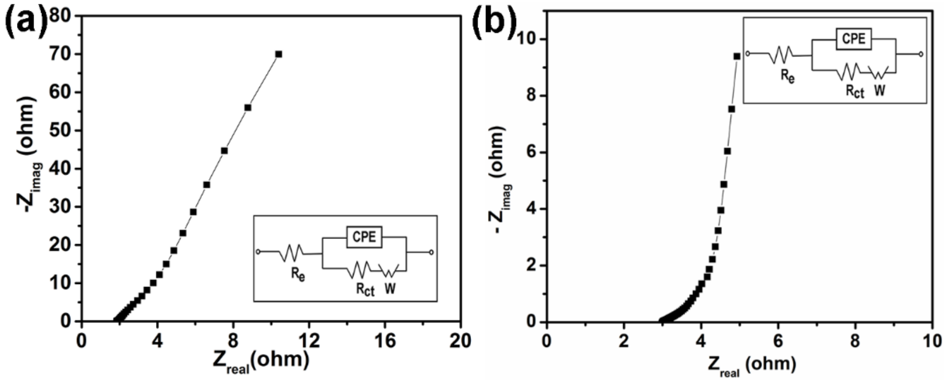

Supplementary Figure 10 | (a) Nyquist plot recorded from 0.1 Hz to 1 MHz for a CNT micro-conduit electrode (the inset shows the equivalent circuit). (b) Nyquist plot recorded from 0.1 Hz to 1 MHz for a CNT/GP micro-conduit electrode (the inset shows the equivalent circuit).

Changes to Manuscript: According to this comment, we have made the following changes on **page 9** of the modified manuscript:

"Comparative Nyquist plots for bare CNT and CNT/GP micro-conduit electrodes recorded from 0.1 Hz to 1 MHz with an alternating current (AC) perturbation amplitude of 5 mV are provided in Supplementary Figure 10, from which bulk electrolyte resistance R_e of CNT and CNT/GP micro-conduit electrodes is measured to be 1.85 and 2.8 Ω, respectively. Moreover, negligible semicircles in the high frequency region from impedance spectra of both electrodes indicates a low charge transfer resistance R_{ct} of CNT and CNT/GP micro-conduit electrodes."

Moreover, we have added comparative Nyquist plots of CNT and CNT/GP micro-conduit electrodes in the modified Supplementary Figure 10 and related statements in the modified Supplementary:

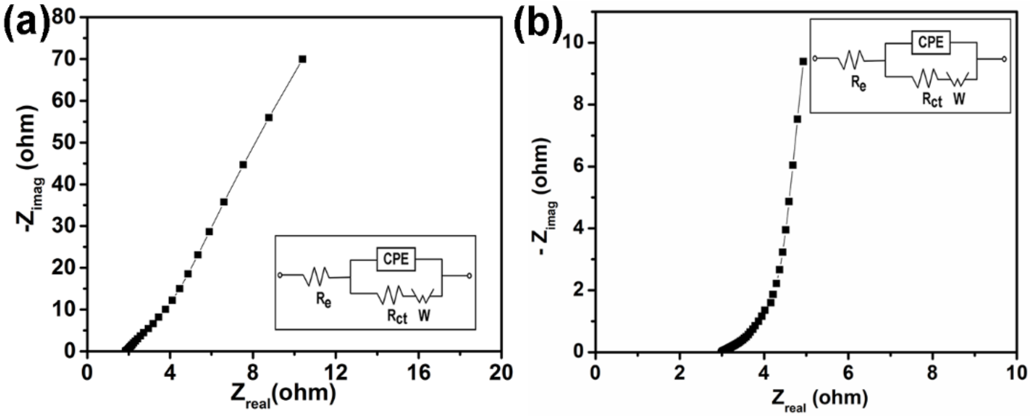

"**Supplementary Figure 10 |** (a) Nyquist plot recorded from 0.1 Hz to 1 MHz for a CNT micro-conduit electrode (the inset shows the equivalent circuit). (b) Nyquist plot recorded from 0.1 Hz to 1 MHz for a CNT/GP micro-conduit electrode. The bulk electrolyte resistance Re of CNT and CNT/GP micro-conduit electrodes is measured to be 1.85 and 2.8 Ω, respectively. Moreover, negligible semicircles in the high frequency region from impedance spectra of both electrodes indicates a low charge transfer resistance R_{ct} of CNT and CNT/GP micro-conduit electrodes."

Reference

1. Yang H, Yang J, Bo Z, Zhang S, Yan J, Cen K. Edge effects in vertically-oriented graphene based electric double-layer capacitors. *J. Power Sources* **324**, 309-316 (2016).

Author response to Review No. 2

Bioinspired Leaves-on-branchlet Hybrid Carbon Nanostructure for Supercapacitors
by
Guoping Xiong, Pingge He, Zhipeng Lyu, Tengfei Chen, Boyun Huang, Lei Chen, Timothy S. Fisher

We thank the reviewer for careful reading of the manuscript. In what follows, we provide a response to each point raised and outline the changes made (in blue font below) to the revised manuscript. For convenience, the various cited references in the response are provided at the end of this document.

"The authors report on the fabrication of micro-conduits based on carbon nanotube/graphene petal (CNT/GP) architecture that show significant improvement on both mechanical and electrochemical properties compared to CNT-based micro-conduits. It is impressive that CNT/GP electrodes yield (1) much higher current density than other nano-structured carbon electrodes and (2) considerably better (specific) capacitance than existing all-carbon electrodes in supercapacitor applications. The authors also demonstrated that the CNT/GP architecture can be used as a nanotemplate for pseudocapacitive materials. As such, I think that this work merits publication in Nature Communication. My only complaint is that explicit reference to the supplementary information is made too often (??), so that it disrupts the flow of the paper. I suggest that the authors make an effort to improve on this aspect."

Authors' Response: We appreciate this comment. We deleted Supplementary Figure 6 and Figure 21 in the original version. Moreover, the manuscript framework has been modified to make the entire paper more integrated and smooth. The corresponding changes have been made throughout the entire manuscript.

Changes to Manuscript: According to this comment, we have deleted the following sentences "Details of the electrochemical activation process are provided in Supplementary Fig. 6" (**page 8**) and "A schematic diagram of the MPCVD chamber for the CNT and GP growth process is shown in Supplementary Fig. 21" (**page 27**) of the original manuscript and modified the manuscript framework to make it more integrated and smooth.

Moreover, Supplementary Figure 6 and Figure 21 in the original Supplementary version have been deleted.

Author response to Review No. 3

Bioinspired Leaves-on-branchlet Hybrid Carbon Nanostructure for Supercapacitors
by
Guoping Xiong, Pingge He, Zhipeng Lyu, Tengfei Chen, Boyun Huang, Lei Chen, Timothy S. Fisher

We thank the reviewer for careful reading of the manuscript. In what follows, we provide a response to each point raised and outline the changes made (in blue font below) to the revised manuscript. For convenience, the various cited references in the response are provided at the end of this document.

"This manuscript reports a new type of interesting carbon nanotube (CNT) arrays/graphene petals (GPs) core-branch all-carbon micro-conduit electrode for supercapacitors."

Comment 1. The growing processes of CNT micro-conduit were recorded, but authors did not provide detailed mechanism of the formation of micro-conduit. Why the outside CNTs grows faster and longer than the inside ones?

Authors' Response: We appreciate this comment. The formation of CNT micro-conduit involves many factors such as growth duration, plasma, metal catalyst and substrate. As demonstrated in Supplementary Figure 2 and Figure 3, the CNT growth duration and plasma have significant influences on the formation of CNT micro-conduits. To further investigate the mechanism, the catalyst effect has also been studied to get a deep understanding of the CNT micro-conduit formation. Various metal layers of only Fe (nominally 5 nm), Ti/Fe (nominally 30/5 nm) and Ti/Al/Fe (nominally 30/10/5 nm) were deposited on carbon cloth respectively as catalysts for CNT growth and results are now included in Supplementary Figure 4 to show the morphologies of products prepared using different catalysts. When utilizing only Fe deposited on carbon cloth as catalyst, few is there CNTs randomly grown on carbon fiber surface (see Figure 4a) and numerous Fe nanoparticles still remain on the surface (see Figure 4b). Figure 4c and 4d show the product morphology when using Ti/Fe as catalyst for CNT growth. Though some CNT micro-conduits form on carbon fiber surface, CNT arrays within the micro-conduit structure grow loosely and the surface of such micro-conduits presents fluffy. When Ti/Al/Fe was used as catalyst, CNT micro-conduits successfully grow on carbon fiber surface. Moreover, highly-ordered CNT arrays grow densely within the micro-conduit (see Figure 4e and 4f).

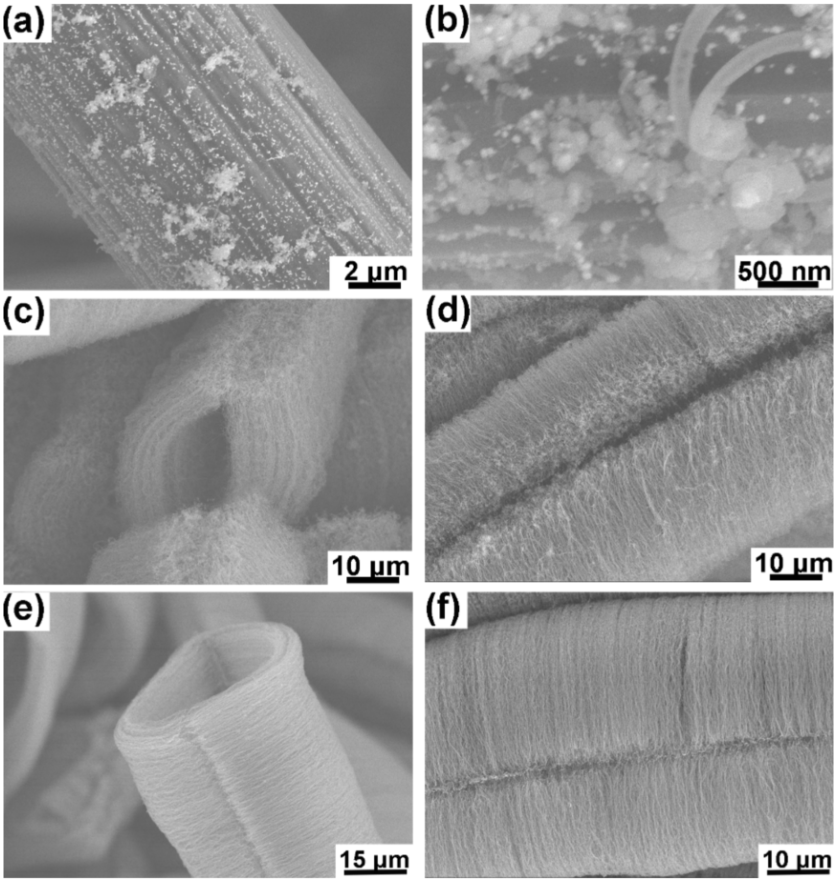

Supplementary Figure 4 | SEM images to show morphologies of CNTs grown on carbon cloth using different metal catalysts: (a, b) Only Fe as catalyst. (c, d) Ti/Fe as catalyst. (e, f) Ti/Al/Fe as catalyst.

Furthermore, we chose substrates with different geometric sizes to grow CNTs, keeping all other experimental parameters the same and results are now included in Supplementary Figure 5 to show comparative SEM images of CNT arrays grown on commercial Buckypaper (within randomly interconnected CNT), carbon cloth (within woven carbon microfibers) and Si wafer. When using Buckpaper as a substrate, within which carbon nanotubes possess a diameter in a range of several tens of nanometers, CNT arrays grow vertically on Buckypaper surface (see Figure 5a), with single CNT interconnected with each other. Subsequently, GPs further decorate on CNT surface, forming an interconnected web structure (see Figure 5b). However, when Si wafer was utilized as a substrate, of which diameter seems to be infinite, CNT arrays grow vertically with high orientation on this flat substrate (see Figure 5c). From a cross-section SEM image in Figure 5d, CNT arrays are well aligned and small GPs were densely and uniformly coated on CNT surface, making it fluffy. CNT micro-conduits were successfully formed only when using carbon cloth as a substrate (see Figure 5e), within which carbon microfibers exhibit a diameter of several micrometers. After further growth of GPs, uniform GPs decorate CNTs along the axial direction on the micro-conduit

surfaces (see Figure 5d). The results reveal that the formation of CNT micro-conduit is highly dependent on the geometric size of substrate.

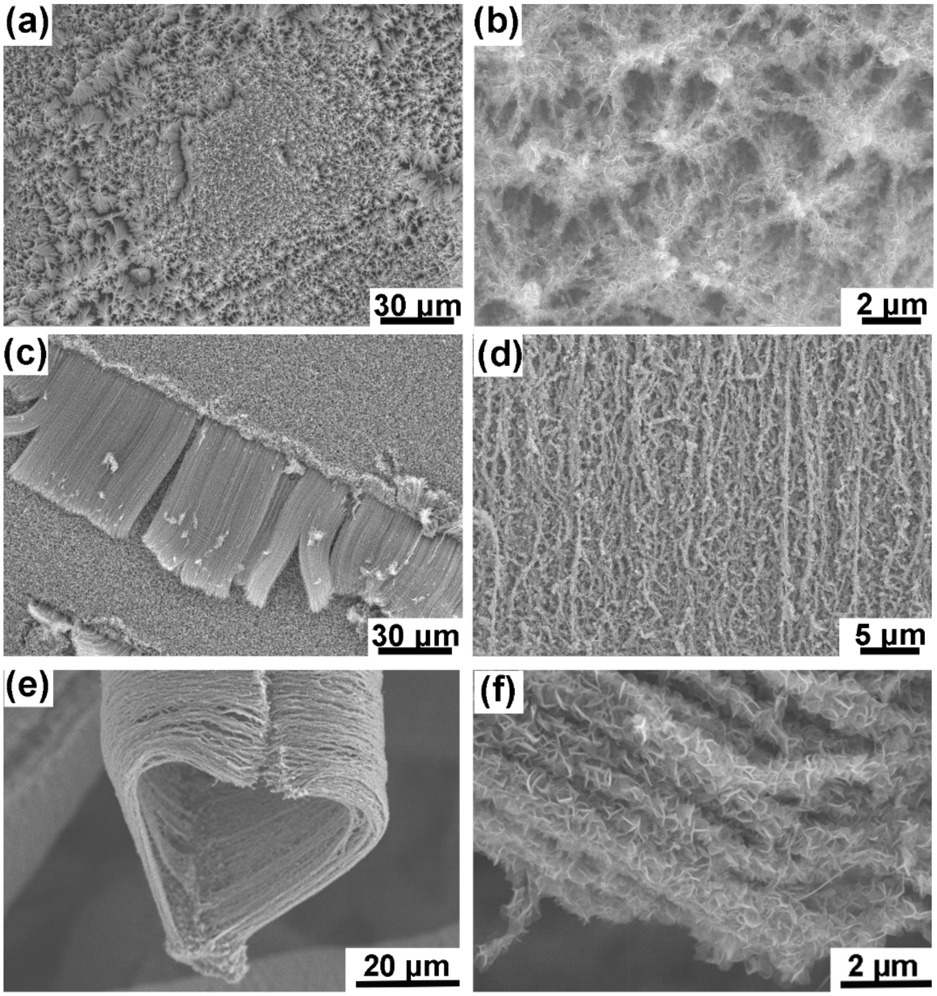

Supplementary Figure 5 | SEM images to show morphologies of CNTs grown on various substrates with further decoration of GPs: (a) Buckypaper as substrate at a low magnification. (b) Buckypaper as substrate at a high magnification. (c) Si wafer as substrate at a low magnification. (d) Si wafer as substrate at a high magnification. (e) Carbon cloth as substrate at a low magnification. (f) Carbon cloth as substrate at a high magnification.

In summary, CNT micro-conduits could successfully form on carbon fiber surface through a MPCVD process for 10 mins by using Ti/Al/Fe as catalyst. Specifically, under the coupling between plasma and carbon fiber, CNTs grow uniformly from catalysts deposited on carbon fibers at an initial stage, and then gradually form two thin open walls with the influence of plasma. Due to the cylinder morphology of carbon fiber (with a diameter of several micrometers), CNT walls incurve gradually as growth time extends. CNTs continue to grow until the tips touch each other, forming a hollow conduit with a microscale size. The formation mechanism of CNT micro-conduits has been

systematically discussed in this work, revealing that various factors such as plasma, growth duration, substrate and catalyst significantly affect the growth of CNT micro-conduits. The formation of CNT micro-conduits is achieved by the synergetic effect between these factors, which might involve complicated processes and will be further exploited in our future work.

Changes to Manuscript: According to this comment, we have made the following changes on **page 5** of the modified manuscript:

"Moreover, the catalyst and substrate effects on the formation of CNT micro-conduit have also been provided in Supplementary Figs. 4 and 5, respectively, indicating that the growth of CNT micro-conduit is highly dependent on triple-layer metal catalyst and the geometric size of substrate."

Moreover, we have added the SEM images of CNTs grown using different metal catalysts in Supplementary Figure 4, SEM images of CNTs grown on various substrates in Supplementary Figure 5 and related statements in the modified Supplementary:

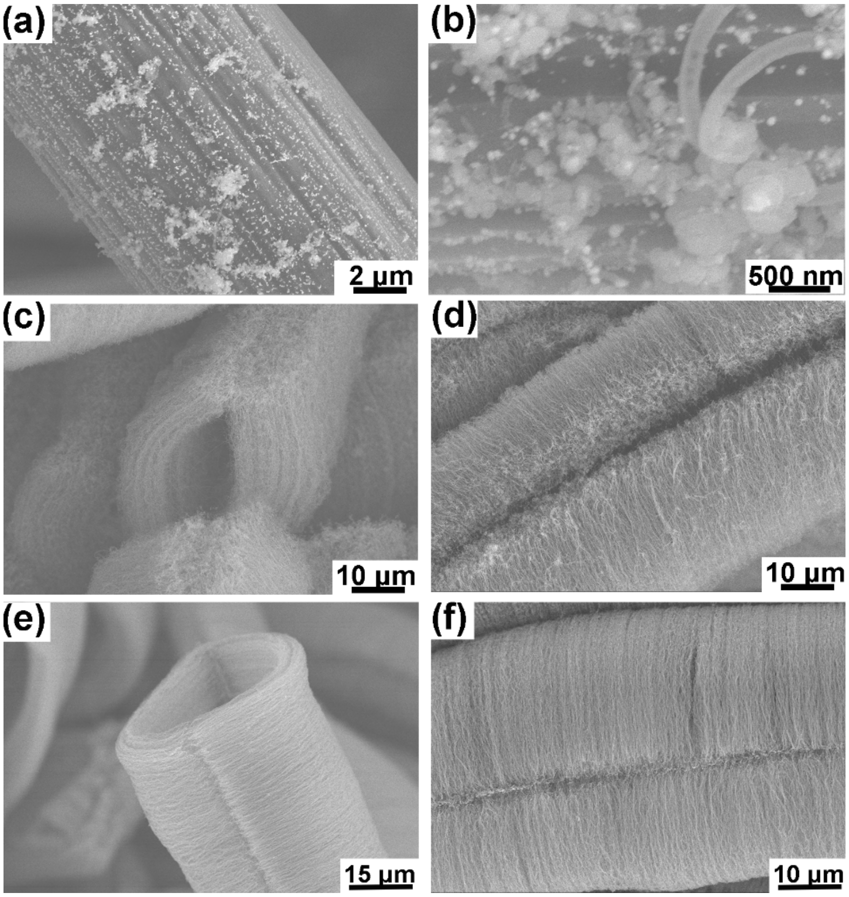

"**Supplementary Figure 4 | SEM images to show morphologies of CNTs grown on carbon cloth using different metal catalysts:** (a, b) Only Fe as catalyst. (c, d) Ti/Fe as catalyst. (e, f) Ti/Al/Fe as catalyst."

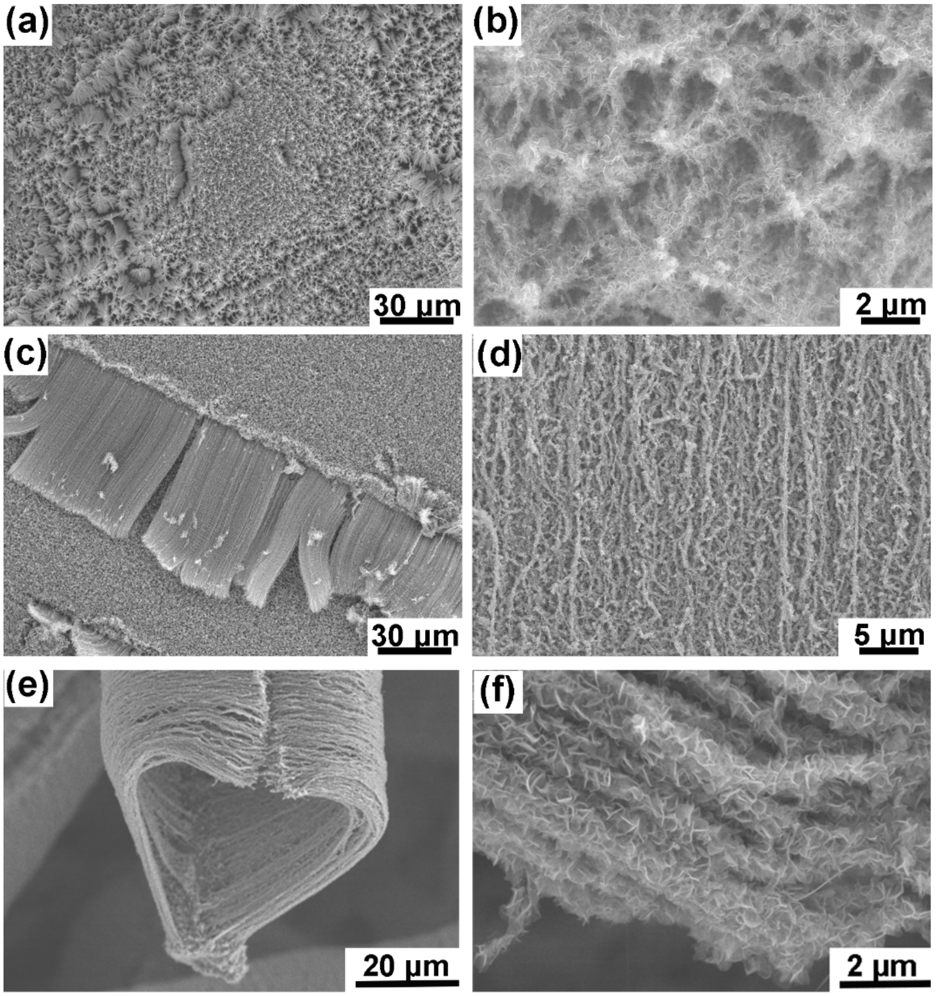

"**Supplementary Figure 5 | SEM images to show morphologies of CNTs grown on various substrates with further decoration of GPs:** Buckypaper as substrate at (a) a low magnification and (b) a high magnification. Si wafer as substrate at (c) a low magnification and (d) a high magnification. Carbon cloth as substrate at (e) a low magnification and (f) a high magnification."

Comment 2. What's the purpose of the Ti/Al/Fe tri-layer catalysts for the CNT micro-conduit growth? How about just one or two?

Authors' Response: The Ti/Al/Fe metal tri-layer was deposited on the carbon cloth surface as catalyst for the CNT micro-conduit growth and metal layers act different roles in the growth of CNT micro-conduits. Ti is deposited to increase the surface roughness and to provide more active nucleation sites.[2] Al is expected to surround the catalyst particles, impeding aggregation of the catalyst and enabling long-term catalytic activity for CNT synthesis.[3] Fe is the active catalyst for the CNT growth.

To investigate the catalyst effect on the CNT micro-conduit growth, various metal layers of only Fe (nominally 5 nm), Ti/Fe (nominally 30/5 nm) and Ti/Al/Fe (nominally 30/10/5 nm) were deposited on carbon cloth respectively as catalysts for CNT growth and results are now included in Supplementary Figure 4 to show the morphologies of products prepared using different catalysts. When utilizing only Fe deposited on carbon cloth as catalyst, few is there CNTs randomly grown on carbon fiber surface (see Figure 4a) and numerous Fe nanoparticles still remain on the surface (see Figure 4b). Figure 4c and 4d show the product morphology when using Ti/Fe as catalyst for CNT growth. Though some CNT micro-conduits form on carbon fiber surface, CNT arrays within the micro-conduit structures grow loosely and the surface of such micro-conduits presents fluffy. When Ti/Al/Fe was used as catalyst, CNT micro-conduits successfully grow on carbon fiber surface. Moreover, highly-ordered CNT arrays grow densely within the micro-conduit structures (see Figure 4e and 4f).

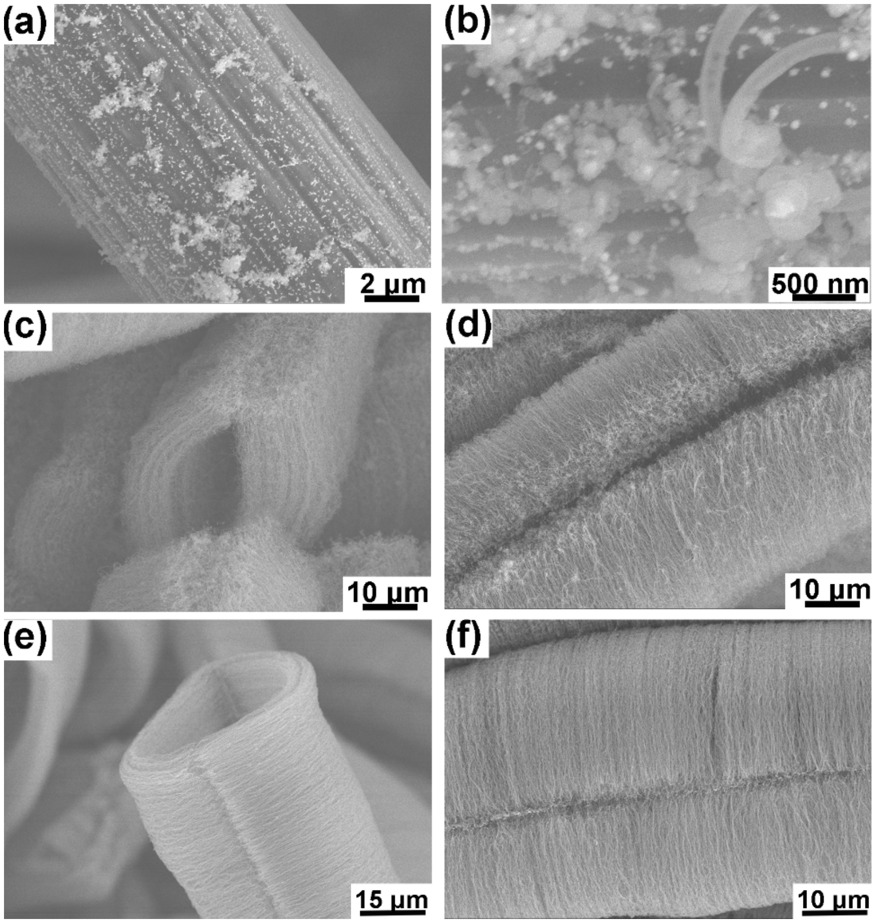

Supplementary Figure 4 | SEM images to show morphologies of CNTs grown on carbon cloth using different metal catalysts: (a, b) Only Fe as catalyst. (c, d) Ti/Fe as catalyst. (e, f) Ti/Al/Fe as catalyst.

Changes to Manuscript: According to this comment, we have made the following changes on **page 5** of the modified manuscript:

"Moreover, the catalyst and substrate effects on the formation of CNT micro-conduit have also been provided in Supplementary Figs. 4 and 5, respectively, indicating that the growth of CNT micro-conduit is highly dependent on triple-layer metal catalyst and the geometric size of substrate."

Additionally, we have added the SEM images of CNTs prepared by various metal catalysts in Supplementary Figure 4 and related statements in the modified Supplementary:

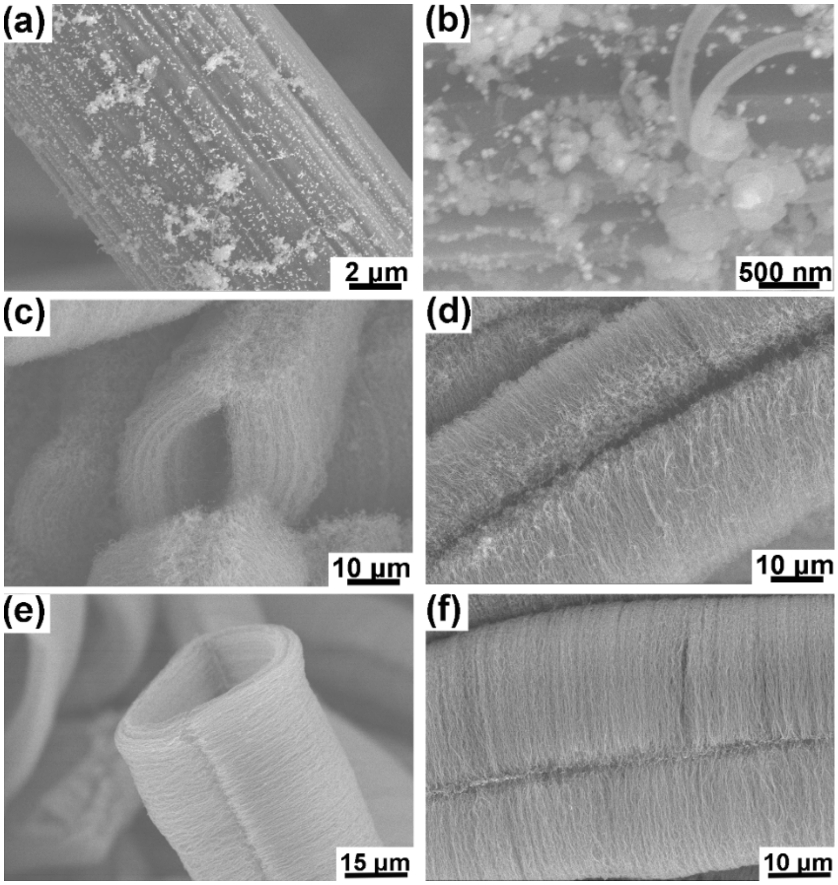

"**Supplementary Figure 4 | SEM images to show morphologies of CNTs grown on carbon cloth using different metal catalysts:** (a, b) Only Fe as catalyst. (c, d) Ti/Fe as catalyst. (e, f) Ti/Al/Fe as catalyst."

<u>Comment 3</u>. How did the authors calculate the mass ratio and loading of the GPs and CNTs. Usually, the mass of substrates (carbon cloth and CNTs in this MS) reduce during the plasma treatment growth of vertical CNTs and graphene. The growth mechanism of GPs on CNT should be introduced.

Authors' Response: We appreciate this comment. The loading of CNT was measured by the weight difference of carbon cloth before and after the MPCVD process using a microbalance with an accuracy of 1 μg. Similarly, the loading of GPs was measured by the weight difference of CC/CNT micro-conduits before and after the MPCVD process using a microbalance with an accuracy of 1 μg. We agree with the reviewer's suggestion that the mass of substrates usually reduce during the plasma treatment and the mass loss during the MPCVD process is inevitable but negligible. Thus, the mass loading is an approximate value to evaluate the specific capacitance of electrode materials while the areal capacitance adopted in this work (or energy and power densities normalized by area) of electrodes is more meaningful and suitable for supercapacitors in practical applications.

The growth mechanism of GPs has been systematically investigated by our previous work in Ref. 4, demonstrating the growth of GPs on CNT micro-conduit substrate by the dissociation of methane in a MPCVD chamber could involve two possible growth processes: (i) a rapid growth mechanism from the top of a GP which is dominated by C species from the decomposition of CH_4, and (ii) a slower growth from the side of a GP which is governed by the precipitation and diffusion of carbon atoms from the substrate, forming multiple layers and thus allowing the GPs to grow in thickness as well as vertical height. There is also an inherent self-limiting aspect to the growth process. When smaller petals merge to form a larger interconnected network, the resultant petal structure forms an electrostatic equipotential surface, reducing the effect of the inhomogeneous, local electric field distribution, leading to a more uniform growth of GPs.

Changes to Manuscript: According to this comment, we have made the following changes on **page 7** and **page 9** of the modified manuscript:

"The GP growth mechanism has been demonstrated in our previous work[25]" (**page 7**).

"With a GP growth time of 18 min, the mass ratio of GPs to CNTs is approximately 4:1, with a total areal mass density of 4.75 mg cm^{-2} (The mass loading was measured by the weight difference of substrate before and after the MPCVD process using a microbalance with an accuracy of 1 μg)." (**page 9**)

<u>Comment 4</u>. **BET should be provided. Morphology of CNT/GP-X and loading of X should be added.**

Authors' Response: We appreciate this comment. The nitrogen (N_2) adsorption–desorption isotherm of CNT/GP micro-conduits on CC is measured and now included as Supplementary Figure 15. The measured BET surface area of CNT/GP micro-conduit electrodes is 30.5970 ± 0.1095 m^2 g^{-1}, nearly 100 times greater than that of CC/GPs (0.32 m^2 g^{-1}) [4] and 300 times greater than that of pure CC (0.11 m^2 g^{-1}),[4] indicating significantly increased specific surface area of CNT/GP micro-conduits. The adsorption average pore diameter of CNT/GP micro-conduit on CC is shown to be 13.2678 nm.

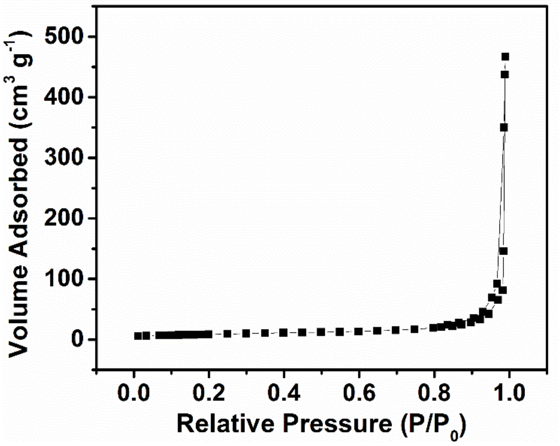

Supplementary Figure 15 | Nitrogen (N_2) adsorption–desorption isotherm of CNT/GP micro-conduit electrodes.

TEM images of CNT/GP/Ni-Co hydroxide micro-conduits have been added in Supplementary Figure 16. As shown in the Supplementary Figure 16a, the Ni-Co hydroxide "small petals" with typical lateral sizes of tens of nanometers are uniformly and densely distributed on the surface of large GP. The visible dark lines are the folded edges, wrinkles or ripples of Ni-Co hydroxide petals. Energy dispersive X-ray spectrometry (EDX) elemental mapping of CNT/GP/Ni-Co hydroxide micro-conduits shown in Supplementary Figure 16b to Figure 16f reveals the spatial elemental distribution within the hierarchical structure. Figure 16c to Figure 16f correspond to C, Ni, Co and O element maps, respectively. These spatial elemental mappings not only confirm the unique hierarchical petal-on-petal structure of GP/Ni-Co hydroxide, but more importantly reveal a homogeneous spatial distribution of the mapped elements (Ni, Co and O) in the Ni-Co hydroxide petal structure. The loading of Ni-Co hydroxide active material is measured by the weight difference of CNT/GP micro-conduit before and after electrodeposition process and the mass loading is approx. 3.5 mg cm^{-2}.

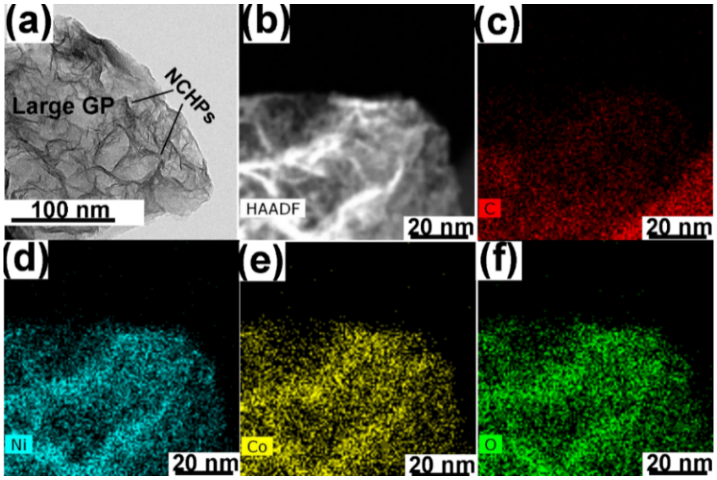

Supplementary Figure 16 | (a) TEM image of a GP covered with a large amount of Ni-Co hydroxide petals (NCHPs). (b) High-angle annular dark field (HAADF) scanning

TEM image of a magnified part of GP covered with many small NCHPs. (c-f) Elemental mapping showing uniform spatial distribution of mapped elements in (b): (c) to (f) correspond to C, Ni, Co and O element maps, respectively.

SEM images of PANI electrodeposited on CNT/GP micro-conduit electrodes are now provided in Supplementary Figure 17. After electrodeposition of PANI for 5 mins, GP surface presents rough and fluffy, indicating a thin layer of PANI electrodeposited on the micro-conduit electrode surface. The loading of PANI active material is measured by the weight difference of CNT/GP micro-conduit before and after electrodeposition process and the mass loading is approx. 1.1 mg cm^{-2}.

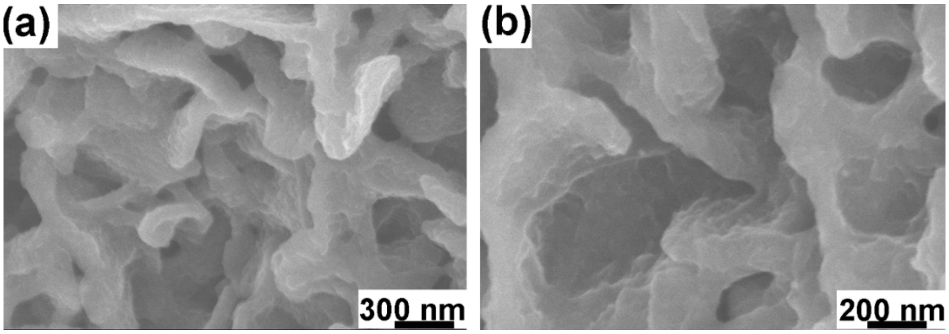

Supplementary Figure 17 | (a) A typical SEM image of CNT/GP/PANI micro-conduit electrodes. (b) Rough surface of electrodes after electrodeposition of PANI.

Changes to Manuscript: According to this comment, we have made the following changes on **page 11** and **page 13** of the modified manuscript:

"The nitrogen (N_2) adsorption–desorption isotherms of CNT/GP micro-conduit electrodes is provided in Supplementary Figure 15 and the measured BET surface area is 30.5970 ± 0.1095 m^2 g^{-1}, nearly 100 times greater than that of CC/GPs (0.32 m^2 g^{-1}) and 300 times greater than that of pure CC (0.11 m^2 g^{-1}), confirming the large surface area of CNT/GP micro-conduit electrodes."

"The morphologies of Ni-Co hydroxide and PANI electrodeposited on nanotemplates are provided in Supplementary Figure 16 and Figure 17, respectively."

Moreover, the nitrogen (N_2) adsorption–desorption isotherm of CNT/GP micro-conduit electrodes has been added in Supplementary Figure 15. TEM images of Ni-Co hydroxide on nanotemplates have been added in Supplementary Figure 16. SEM images of PANI on nanotemplates have been added in Supplementary Figure 17.

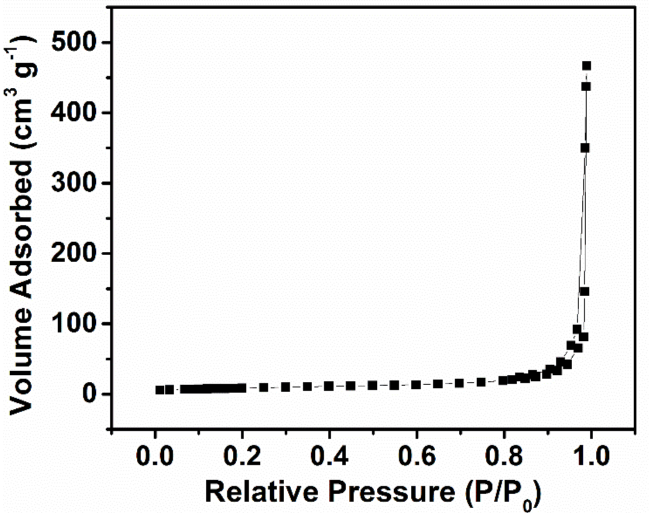

Supplementary Figure 15 | Nitrogen (N_2) adsorption–desorption isotherm of CNT/GP micro-conduit electrodes.

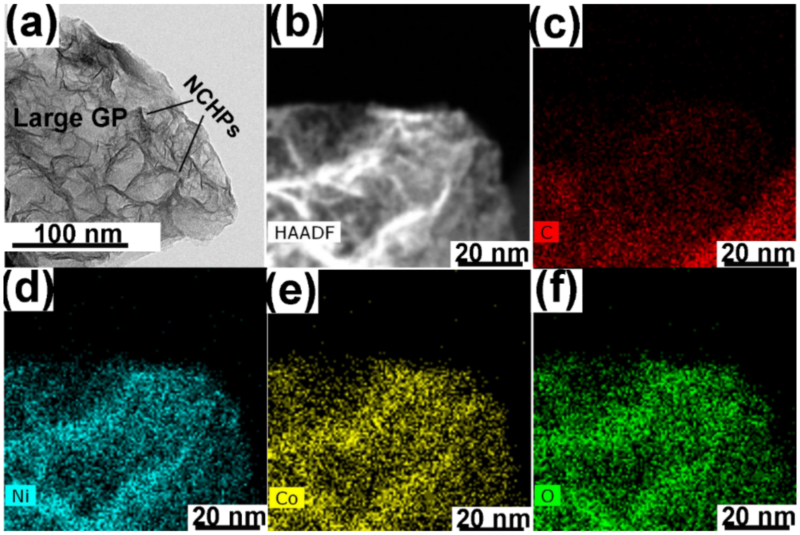

Supplementary Figure 16 | (a) TEM image of a GP covered with a large amount of Ni-Co hydroxide petals (NCHPs). (b) High-angle annular dark field (HAADF) scanning TEM image of a magnified part of GP covered with many small NCHPs. (c-f) Elemental mapping showing uniform spatial distribution of mapped elements in (b): (c) to (f) correspond to C, Ni, Co and O element maps, respectively.

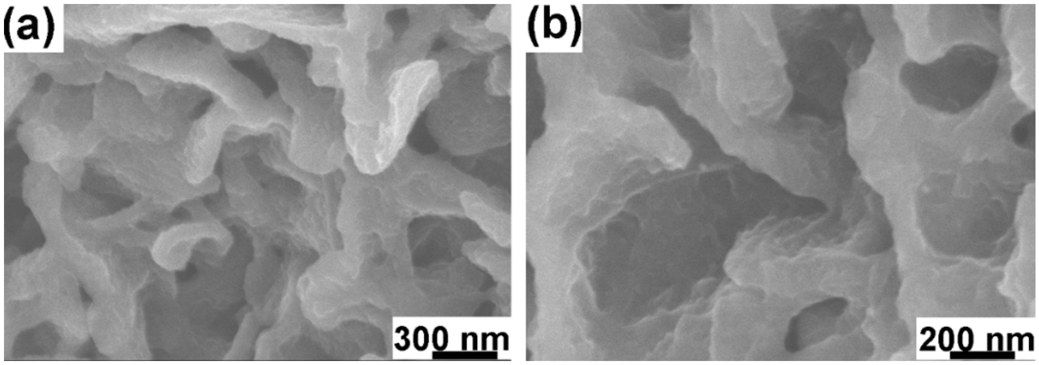

Supplementary Figure 17 | (a) A typical SEM image of CNT/GP/PANI micro-conduit electrodes. (b) Rough surface of electrodes after electrodeposition of PANI.

Comment 5. The mechanistic study by modelling the charge and electric field distributions is not so significant (also reported previously in a Journal of power source paper). In fact, such modelling should not be specific to graphene, but very fundamental to any material of tapered morphology. So not sure if it's necessary to put so much elaboration in main text.

Authors' Response: We greatly appreciate this comment. Here we modestly emphasize the significance of the modeling part here. Firstly, to render the atomically graphene-informed modelling, we have combined the Nernst-Planck-Poisson calculations and the atomic level molecular dynamics simulations (MD). Specifically, the size effect of the ion diffusion coefficients in the confined graphene nanochannels is investigated using MD simulations. Secondly, we would like to clarify that our objective is to design a bioinspired leaves-on-branchlet hybrid carbon nanostructure, and our Nernst-Planck-Poisson calculations thus aim to investigate the underlying mechanisms of charge transfer and storage, as well as ion diffusion for such a specific structure, based on experimentally measured and/or previously reported materials (e.g., graphene) properties and structural geometries. To the best of our knowledge, the paper[1] in the Journal of Power Source reports a molecular dynamics simulation of only a simplified vertically oriented single-layer graphene arrays, and it is difficult to extend the model to the proposed leaves-on-branchlet structure limited to its high-cost computation. Moreover, we also want to emphasize that the current computational model applicable to various materials is expected to be more promising to inspire new material combinations for this specific structure.

Changes to Manuscript: According to this comment, we condensed the modeling part in the revised manuscript:

"**Nernst-Planck-Poisson modeling.** Nernst-Planck-Poisson (NPP) calculations of the Gouy-Chapman model were employed to elucidate the underlying mechanisms of charge transfer and storage, as well as ion diffusion, governed by the Poisson equation and the Nernst-Plank equation respectively. The governing equations were simultaneously solved

with an implicit algorithm for the purpose of iterative coupling by the finite element (FE) method using the commercial software COMSOL 5.2. The FE-based NPP calculations run with the inputs of properties from the molecular dynamics simulations (the schematic representation of the MD simulation systems has been provided in Figure S26) to render the atomically informed modelling. The simulation details are provided in Supporting Information."

Moreover, we moved the fundamental Nernst-Planck-Poisson equations and MD simulation details into the Supplementary. A MD schematic has also been added in Supplementary Fig. 26 (**page 28**) to show the atomic arrangement.

"**Nernst-Planck-Poisson modeling details:**

Nernst-Planck-Poisson (NPP) calculations of the Gouy-Chapman model were employed to elucidate the underlying mechanisms of charge transfer and storage, as well as ion diffusion, governed by the Poisson equation

$$\nabla \cdot (\varepsilon_0 \varepsilon_r \nabla \varphi) = -\sum_i z_i e C_i \tag{1}$$

and the Nernst-Plank equation

$$\frac{\partial C_i}{\partial t} = \nabla \cdot \left[D_i \nabla C_i + \frac{D_i C_i}{k_B T} z_i e \nabla \varphi \right] \tag{2}$$

respectively, where ε_0 is the permitivity of vacuum, ε_r is the relative permittivity of the medium, φ is the elestrostatic potential, D_i is the diffusivity of chemical species i, C_i is the density of the species, z_i is the valency of the species, e is the elementary charge, k_B is Boltzmann's constant, and T is temperature."

"**Molecular Dynamics simulation details:**

Molecular dynamics (MD) simulations were performed to calculate the size effect of the ion diffusion coefficients in the confined graphene nanochannels. Fig. S26 shows a schematic representation of the simulation model of confined eletrolyte NaCl. The simulations were performed in a cubic box at room temperature. The pressure for bulk systems is 300 ±150 atmospheres, similar to in the literature.[22] The results show that the behavior of ions diffusion is almost unchanged once the size of confined nanochannels exceed 5 nm. According to Ref. [23], the effective ionic diameters of NaCl eletrolyte and H_2SO_4 electrolyte are basically comparable, as 0.19-0.76 nm for Na^+, 0.36-0.66 nm for Cl^-, 0.22-0.56 nm for H^+ and 0.58-0.76 nm for SO_4^{2-}. Thus, such a over-5 nm size-indepence bahavior is fairly spectulated for the H_2SO_4 electrolyte. Based on statistically calculation of experimental results indicates the gap between two graphene petals are about 10-40 nm. In this regard, the size effect of confined nanochannels is reasonably ignored."

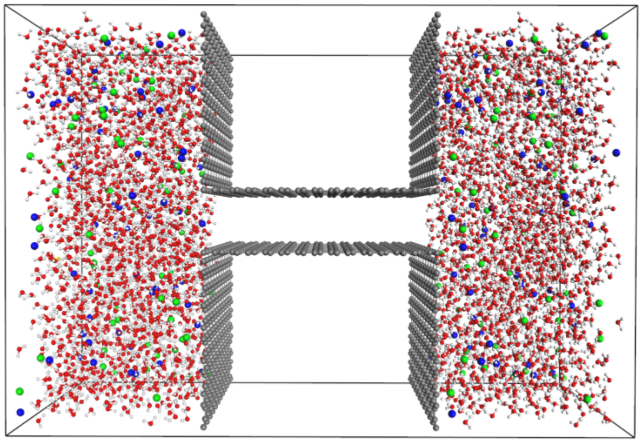

"**Supplementary Figure 26|** Schematic representation of the Molecular dynamics simulation systems for a confined NaCl electrolyte. Grey spheres represent graphene carbon atoms, where two face-to-face graphene sheets construct a channel. Blue spheres represent Na^{+} ions, green spheres represent Cl^{-} ions, and red and white spheres represent oxygen and hydrogen atoms of water, respectively."

Reference

1. Yang H, Yang J, Bo Z, Zhang S, Yan J, Cen K. Edge effects in vertically-oriented graphene based electric double-layer capacitors. *J. Power Sources* **324**, 309-316 (2016).
2. Delzeit L, Chen B, Cassell A, Stevens R, Nguyen C, Meyyappan M. Multilayered metal catalysts for controlling the density of single-walled carbon nanotube growth. *Chem. Phys. Lett.* **348**, 368-374 (2001).
3. Maschmann MR*, et al.* Vertical single-and double-walled carbon nanotubes grown from modified porous anodic alumina templates. *Nanotech.* **17**, 3925 (2006).
4. Xiong G, Hembram K P S S, Zakharov D N, et al. Controlled thin graphitic petal growth on oxidized silicon. Diam. Relat. Mater. **3**, 1-9 (2012).
5. Xiong G, Meng C, Reifenberger RG, Irazoqui PP, Fisher TS. Graphitic Petal Electrodes for All-Solid-State Flexible Supercapacitors. *Adv Energy Mater* **4**, (2014).

As the reviewers suggested, we herewith resubmit the revised manuscript to *Nature Communications*. All authors of this research paper have read and approved the final version submitted. The contents of this manuscript have not been copyrighted or published in whole or in part, nor is it being considered for publication elsewhere. We thank you for your time and kind consideration of this manuscript.

Sincerely yours,

Timothy S. Fisher
Professor of Mechanical & Aerospace Engineering, UCLA

Reviewers' comments:

Reviewer #1 (Remarks to the Author):

The revised manuscript can be accepted in the current form.

Reviewer #3 (Remarks to the Author):

I do appreciate the efforts authors have spent in making such a 25-page long response particularly to comments by Referee 3, and revising the manuscript. It took a lot of patience to read through. I fully agree that this structure is really fancy, more robust, and advantageous in supercapacitive property. Modelling also supports that this all-carbon unique structure has an outstanding charge transfer kinetics which is the key to high area capacitance and rate property. The reasons why I think publication of this work in NC is optional are as follows. This structure needs a complicated synthesis process and maybe also recipe, so that not many groups will be able to follow. The fabrication also requires an expensive MPCVD system, which prevents the practical application of this material in supercapacitor applications relative to the commercial activated carbon. If Editor thinks this is not a relevant issue, please go ahead to publish.

Reviewer #4 (Remarks to the Author):

This manuscript demonstrated a carbon nanotube/graphene core-branch micro-conduit electrode for supercapacitor application, which is interesting. After carefully reading throughout the manuscript, the authors failed to address some important issues even in the revised manuscript. Therefore, this manuscript may be considered to be published in Nature Communications if the following issues were well addressed.

1. The mechanism of the formation of CNT micro-conduit was not convincing even many supplementary experiments have been done. As the authors demonstrated, the formation of CNT micro-conduit is highly dependent on the geometric size of substrate, it is write, but failed to explain why. The catalyst was deposited onto the carbon cloth from both sides, which must result a gradually different thickness of catalyst layer around the carbon fibers because of the cylindrical structure of carbon fibers. The different thick catalyst layer will lead different sizes of catalyst nanoparticle for CNT growth, which resulting different catalytic activity and growth rate. This is the keypoint!

2. The capacitance of supercapacitors based on CNT/graphene micro-conduit decreased as the graphene growth time increased (Figure S9), how about the CNT/graphene micro-conduit with growth time of 10 min and 5 min, did their capacitance increase or decrease?

3. In the revised manuscript, only one BET data of CNT/graphene micro-conduit was provided, how about other samples with different graphene growth time. The specific surface area and the pore size of electrodes largely determine the performance of the resulting supercapacitors, which can provide a convincing evidence of your results!

4. To calculate the gravimetric specific capacitance (500 F/g) of supercapacitors based on CNT/graphene micro-conduits and their composites, the mass loading of CNT, graphene and other pseudocapacitive materials should be evaluated convincingly. However, the explanation on calculation of CNT/graphene loading is not acceptable in the response to the comments, because very little deviation may result the specific capacitance overevaluated.

5. “…the bulk electrolyte resistance Re of CNT and CNT/GP micro-conduit electrodes is measured…” in Page 9 and other places in the manuscript is not correct. The X-axis intercept of Nyquist Plot represents the series resistance derived from electrode, electrolyte and the interface between electrode and electrolyte.

6. Did the CNT/GP micro-conduit well maintain their 3D micro-conduit structure after loading PANI and Ni-Co hydroxide? It cannot be observed from Figure S16 and S17.

Author response to Review No. 1

Bioinspired Leaves-on-branchlet Hybrid Carbon Nanostructure for Supercapacitors

by

Guoping Xiong, Pingge He, Zhipeng Lyu , Tengfei Chen, Boyun Huang, Lei Chen, Timothy S. Fisher

We thank the reviewer for careful reading of the manuscript. In what follows, we provide a response to each point raised and outline the changes made to the revised manuscript (in blue font below). For convenience, the various cited references in the response are provided at the end of this document.

"The revised manuscript can be accepted in the current form."

Authors' Response: We thank the reviewer for careful reading and recognition of the importance of our work.

Author response to Review No. 3

Bioinspired Leaves-on-branchlet Hybrid Carbon Nanostructure for Supercapacitors

by

Guoping Xiong, Pingge He, Zhipeng Lyu, Tengfei Chen, Boyun Huang, Lei Chen, Timothy S. Fisher

We thank the reviewer for careful reading of the manuscript. In what follows, we provide a response to each point raised and outline the changes made to the revised manuscript (in blue font below). For convenience, the various cited references in the response are provided at the end of this document.

"I do appreciate the efforts authors have spent in making such a 25-page long response particularly to comments by Referee 3, and revising the manuscript. It took a lot of patience to read through. I fully agree that this structure is really fancy, more robust, and advantageous in supercapacitive property. Modelling also supports that this all-carbon unique structure has an outstanding charge transfer kinetics which is the key to high area capacitance and rate property. The reasons why I think publication of this work in NC is optional are as follows. This structure needs a complicated synthesis process and maybe also recipe, so that not many groups will be able to follow. The fabrication also requires an expensive MPCVD system, which prevents the practical application of this material in supercapacitor applications relative to the commercial activated carbon. If Editor thinks this is not a relevant issue, please go ahead to publish."

Authors' Response: We appreciate this comment. At the laboratory scale, MPCVD has been widely applied in the preparation of carbon structures such as diamond, CNT and graphene[1,2,3,4,5], and other types of nanomaterials such as metal oxides, silicon oxides, silicon carbides[6,7,8]. Compared to conventional CVD, MPCVD is more effective, less time-consuming and more controllable in designing tailored nanostructures for practical applications because of the existence of plasma[9,10,11]. The bioinspired leaves-on-branchlet hybrid structure with GPs grown on CNT micro-conduit was prepared through a two-step MPCVD process conducted within the same chamber to simplify the fabrication process of the hierarchical all-carbon electrode materials. The only difference between the two-step MPCVD growth processes is the varied growth conditions such as plasma power, growth time, and pressure. Therefore, the one-chamber, two-step fabrication process and corresponding recipes should be relatively easy for most research groups and eventually industrial users to follow.

For industrial applications, MPCVD is also a quite promising, cost-effective method for large-scale synthesis of graphene electrode materials [12,13,14]. In fact, in the past several years, our group and others have developed roll-to-roll fabrication of graphene and its derivatives at a large scale and with low cost. For instance, in one

of our ongoing projects, substrates such as Ni foam, carbon cloth, and Cu wire/foil can be transferred through the plasma zone by two controlled rollers at a constant speed in the chamber for large-scale GP growth (see Figure 1a). As typical examples, Figure 1b and 1c show SEM images of GPs grown by roll-to-roll RF plasma CVD on carbon cloth and cotton/stainless steel substrates, respectively. These results clearly demonstrate the feasibility of large-scale of GP synthesis by RF roll-to-roll CVD and offer promise for practical supercapacitor applications in industry. The project is ongoing, and this topic will be explained and discussed in the future work beyond the focus of this manuscript.

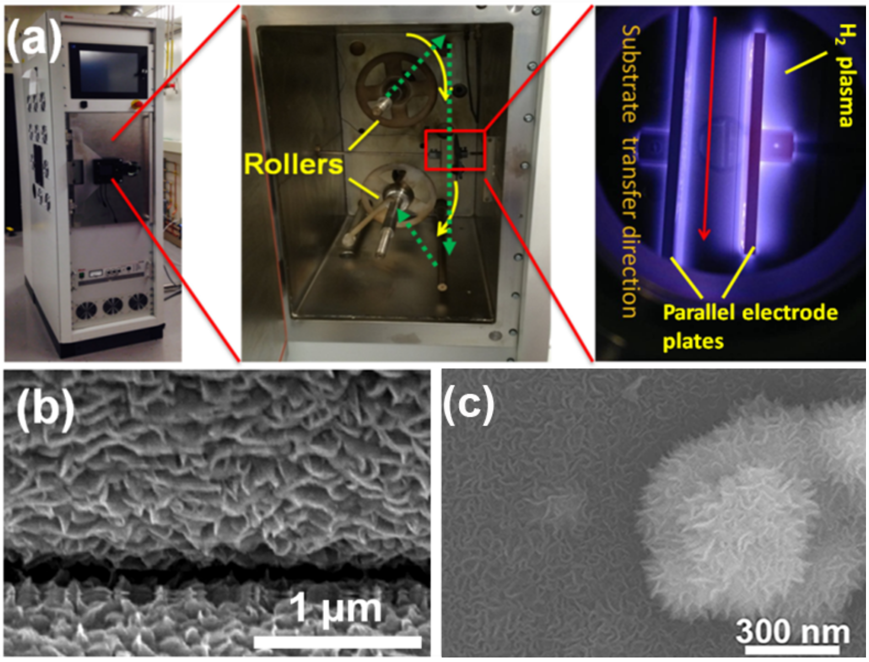

Figure 1. Scalable fabrication of GPs by roll-to-roll RF plasma CVD process. (a) The roll-to-roll RF plasma CVD system with a close-up of the chamber in which two rollers are installed to transfer substrates through the plasma zone defined by two parallel electrodes and substrates can be transferred through plasma zone generated between two parallel electrodes to realize scalable GP growth for industrial applications. (b) Scalable GP growth on carbon cloth substrate by roll-to-roll RF plasma CVD. (c) Scalable GP growth on cotton/stainless steel substrate by roll-to-roll RF plasma CVD.

Changes to Manuscript: According to this comment, we have made the following changes on **page 31** of the modified Supplementary Methods:

"Compared to conventional CVD methods, MPCVD is more efficient, less time-consuming and more controllable in designing tailored nanostructures for

practical applications because of the existence of plasma[19, 22, 21]. The bioinspired leaves-on-branchlet hybrid structure with GPs grown on CNT micro-conduit was prepared through a two-step MPCVD process conducted within the same chamber that simplifies the fabrication process of the hierarchical all-carbon electrode materials. The only difference between the two-step MPCVD growth processes is the variation of growth conditions such as plasma power, growth time, and pressure. Furthermore, for industrial applications, MPCVD is also a quite promising, cost-effective method for large-scale synthesis of graphene electrode materials."

References

1. Bower C, Zhou O, Zhu W, Werder D, Jin S. Nucleation and growth of carbon nanotubes by microwave plasma chemical vapor deposition. *Applied Physics Letters* **77**, 2767-2769 (2000).

2. Wu Y, Qiao P, Chong T, Shen Z. Carbon nanowalls grown by microwave plasma enhanced chemical vapor deposition. *Advanced Materials* **14**, 64-67 (2002).

3. Choi YC*, et al.* Controlling the diameter, growth rate, and density of vertically aligned carbon nanotubes synthesized by microwave plasma-enhanced chemical vapor deposition. *Applied physics letters* **76**, 2367-2369 (2000).

4. Malesevic A*, et al.* Synthesis of few-layer graphene via microwave plasma-enhanced chemical vapour deposition. *Nanotechnology* **19**, 305604 (2008).

5. Kobashi K, Nishimura K, Kawate Y, Horiuchi T. Synthesis of diamonds by use of microwave plasma chemical-vapor deposition: Morphology and growth of diamond films. *Physical review B* **38**, 4067 (1988).

6. Guruvenket S, Andrie S, Simon M, et al. Atmospheric-pressure plasma-enhanced chemical vapor deposition of a-SiCN:H films: role of precursors on the film growth and properties. *Acs Applied Materials & Interfaces* **4**, 5293 (2012).

7. Tanaka H, Chuanjie Z, Hayakawa Y, et al. High-Quality Silicon Oxide Film Formed by Diffusion Region Plasma Enhanced Chemical Vapor Deposition and Oxygen Radical Treatment Using Microwave-Excited High-Density Plasma. *Japanese Journal of Applied Physics* **42**, 1911-1915 (2003).

8. Tyczkowski J, Kapica R, Łojewska J. Thin cobalt oxide films for catalysis deposited by plasma-enhanced metal–organic chemical vapor deposition. *Thin Solid Films* **16**, 6590-6595 (2007).

9. Meyyappan M. A Review Of Plasma Enhanced Chemical Vapour Deposition Of Carbon Nanotubes. *Journal of Physics D Applied Physics* **42**, 103-109 (2009).

10. Kobashi K, Nishimura K, Kawate Y, et al. Synthesis of diamonds by use of microwave plasma chemical-vapor deposition: Morphology and growth of diamond films. *Physical Review B Condensed Matter* **38**, 4067 (1988).

11. Bo Z, Yang Y, Chen J, et al. Plasma-enhanced chemical vapor deposition synthesis of vertically oriented graphene nanosheets. *Nanoscale* **5**, 5180 (2013).

12. Yamada T, Ishihara M, Hasegawa M. Large area coating of graphene at low temperature using a roll-to-roll microwave plasma chemical vapor deposition. *Thin Solid Films* **532**, 89-93 (2013).

13. Jiang L*, et al.* Controlled synthesis of large- scale, uniform, vertically standing graphene for high- performance field emitters. *Advanced Materials* **25**, 250-255 (2013).

14. Kim J, Ishihara M, Koga Y, Tsugawa K, Hasegawa M, Iijima S. Low-temperature synthesis of large-area graphene-based transparent conductive films using surface wave plasma chemical vapor deposition. *Applied physics letters* **98**, 091502 (2011).

Author response to Review No. 4

Bioinspired Leaves-on-branchlet Hybrid Carbon Nanostructure for Supercapacitors
by
Guoping Xiong, Pingge He, Zhipeng Lyu, Tengfei Chen, Boyun Huang, Lei Chen, Timothy S. Fisher

We thank the reviewer for careful reading of the manuscript. In what follows, we provide a response to each point raised and outline the changes made to the revised manuscript (in blue font below). For convenience, the various cited references in the response are provided at the end of this document.

"This manuscript demonstrated a carbon nanotube/graphene core-branch micro-conduit electrode for supercapacitor application, which is interesting. After carefully reading throughout the manuscript, the authors failed to address some important issues even in the revised manuscript. Therefore, this manuscript may be considered to be published in Nature Communications if the following issues were well addressed."

Comment 1. The mechanism of the formation of CNT micro-conduit was not convincing even many supplementary experiments have been done. As the authors demonstrated, the formation of CNT micro-conduit is highly dependent on the geometric size of substrate, it is write, but failed to explain why. The catalyst was deposited onto the carbon cloth from both sides, which must result a gradually different thickness of catalyst layer around the carbon fibers because of the cylindrical structure of carbon fibers. The different thick catalyst layer will lead different sizes of catalyst nanoparticle for CNT growth, which resulting different catalytic activity and growth rate. This is the keypoint!

Authors' Response: We greatly appreciate the reviewer's comment on the growth mechanism of CNT micro-conduits, and we agree in principle with the reviewer's viewpoint. As demonstrated in Supplementary Figure 5, CNT micro-conduits were successfully formed only when using carbon cloth as a substrate, within which carbon microfibers exhibit a diameter of several micrometers. We agree with the reviewer's comment that the catalyst layers (Ti/Al/Fe: 30/10/5 nm) were deposited onto the carbon cloth from both sides, resulting in a gradually varying thickness of catalyst layers around the carbon fibers because of the cylindrical structure of carbon fibers. The different thickness of catalyst layers will lead to different sizes of catalyst nanoparticles depending on the surface mobilities of the species, resulting in different catalytic activities and thus different CNT growth rates and morphologies. Consequently, as growth time extends, the height of CNT walls increases, and eventually the tips of the two walls touch each other under the influence of plasma, forming a hollow conduit with microscale size.

Changes to Manuscript: According to this comment, we have made the following changes on **page 6** of the modified manuscript:

"Due to the cylindrical structure of carbon microfibers, the varying thickness of catalyst layers on these microfibers will lead to different sizes of catalyst nanoparticles, resulting in different catalytic activity and thus different CNT growth rates and morphologies."

Moreover, we have made the following changes on **page 6** of the modified Supplementary Figure 5:

"Supplementary Figure 5 | SEM images of morphologies of CNTs grown on various substrates with further decoration of GPs: Buckypaper as substrate at (a) low magnification and (b) high magnification. Si wafer as substrate at (c) low magnification and (d) high magnification. Carbon cloth as substrate at (e) low magnification and (f) high magnification. CNT micro-conduits successfully formed only when using carbon cloth as a substrate, within which carbon microfibers exhibit a diameter of several micrometers. The catalyst layers (Ti/Al/Fe: 30/10/5 nm) were deposited on both sides of the carbon cloth substrates, resulting in a gradually varying thickness of catalyst layers around carbon fibers because of the cylindrical structure of the carbon fibers. The different thicknesses of catalyst layers lead to different sizes of catalyst nanoparticles, resulting in different catalytic activity and thus different CNT growth rates and morphologies."

<u>Comment 2</u>. The capacitance of supercapacitors based on CNT/graphene micro-conduit decreased as the graphene growth time increased (Figure S9), how about the CNT/graphene micro-conduit with growth time of 10 min and 5 min, did their capacitance increase or decrease?

Authors' Response: We appreciate this concern. We have additionally provided the capacitances (at different scan rates) of electrodes based on CNT/GP micro-conduit structures with GP growth times of 10 min and 5 min in modified Supplementary Figure 9. As shown in Supplementary Figure 9, when the GP growth time increased from 5 to 18 min, the capacitances of CNT/GP micro-conduit electrodes significantly increase at all scan rates. The significantly increased capacitance is attributed to the higher specific surface area and edge density of GPs. However, with further increase of GP growth time, the capacitance of CNT/GP micro-conduit electrodes decreases at higher scan rates. The result indicates that longer GP growth time produces more GPs on the CNT micro-conduit surface, thus increasing the resistance of ion transport into nanopores at higher scan rates and thereby reducing the effective accessible surface area of electrodes. These results

indicate that an optimal GP growth time exists for superior electrochemical performance because of a balance between surface area and ion transfer kinetics.

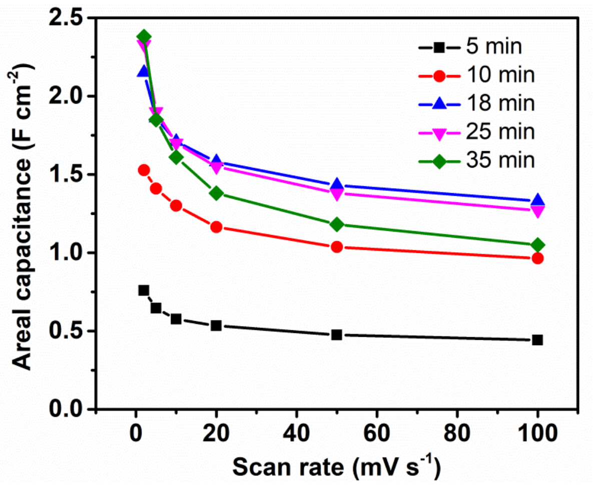

Supplementary Figure 9 | Areal capacitances of electrodes with different GP growth durations (5, 10, 18, 25 and 35 min) as a function of scan rates.

Changes to Manuscript: According to this comment, we have made the following changes on **page 10** of the modified manuscript:

"The areal capacitances of CNT/GP micro-conduit electrodes with different GP growth durations (5, 10, 18, 25 and 35 min) have been plotted as a function of scan rates in Supplementary Fig. 9".

Moreover, we have added comparative capacitances of CNT/GP micro-conduit electrodes with GP growth durations of 5 and 10 min on **page 10** of the modified Supplementary Figure 9 and related statements in the modified Supplementary Information:

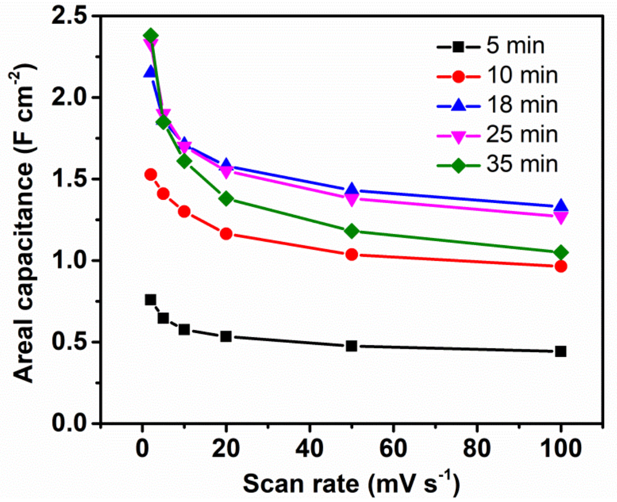

"**Supplementary Figure 9** | Areal capacitances of electrodes with different GP growth durations (5, 10, 18, 25 and 35 min) as a function of scan rates. The results

reveal a relationship between the edge density of GPs and capacitance of CNT/GP micro-conduit electrodes, and indicate that an optimal GP growth time should exist for superior electrochemical performance because of a balance between surface area and ion transfer kinetics."

Comment 3. In the revised manuscript, only one BET data of CNT/graphene micro-conduit was provided, how about other samples with different graphene growth time. The specific surface area and the pore size of electrodes largely determine the performance of the resulting supercapacitors, which can provide a convincing evidence of your results!

Authors' Response: (Note: We experienced some equipment difficulties with BET measurements when we first began this revision; much of the delay in our response was due to this factor, which was resolved a few weeks ago). We appreciate the reviewer's comment. We agree that the specific surface area and the pore size of electrodes largely determine the performance of the resulting supercapacitors. Therefore, nitrogen (N_2) adsorption–desorption isotherms of bare CNT micro-conduits and CNT/GP micro-conduits with GP growth durations of 10 min, 18 min and 45 min have been chosen as typical examples for comparison, and the corresponding BET data have been additionally provided in modified Supplementary Figure 15. The measured BET specific surface areas of CNT/GP micro-conduit with GP growth times of 0 min (bare CNT micro-conduit), 10 min, 18 min and 45 min are 15.5 $m^2 g^{-1}$, 23.8 $m^2 g^{-1}$, 30.6 $m^2 g^{-1}$, and 26.2 $m^2 g^{-1}$, respectively. The BET surface area of CNT/GP micro-conduit with GP growth time of 18 min (30.6 $m^2 g^{-1}$) is nearly 2 times greater than that of bare CNT micro-conduit structure (15.5 $m^2 g^{-1}$), 100 times greater than that of CC/GPs (0.32 $m^2 g^{-1}$) and 300 times greater than that of pure CC (0.11 $m^2 g^{-1}$), confirming the large surface area of CNT/GP micro-conduit electrodes.

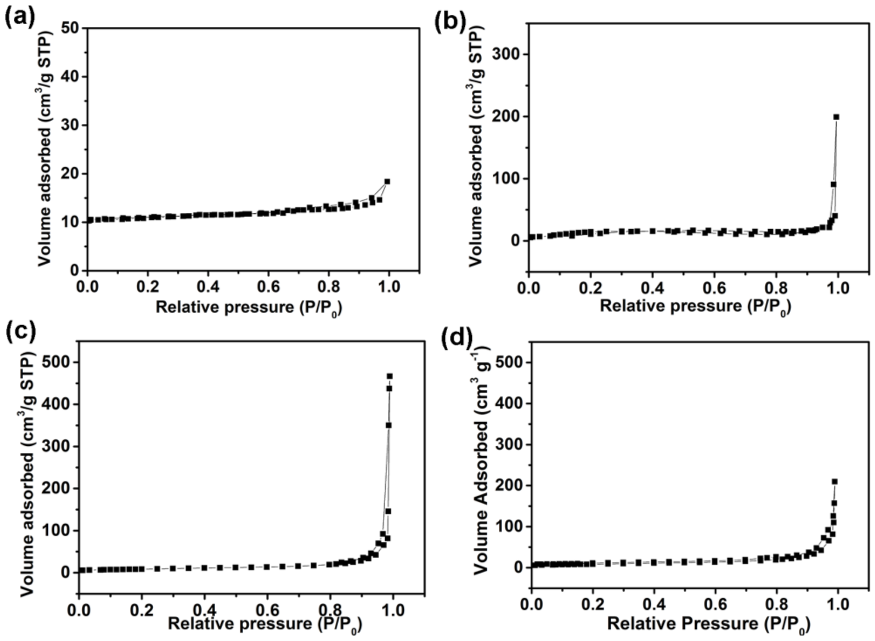

Supplementary Figure 15 | The nitrogen (N_2) adsorption–desorption isotherm curves of (a) bare CNT micro-conduit electrode, (b) CNT/GP micro-conduit electrode with GP growth time of 10 min, (c) CNT/GP micro-conduit electrode with GP growth time of 18 min, (d) CNT/GP micro-conduit electrode with GP growth time of 45 min

Changes to Manuscript: According to this comment, we have made the following changes on **page 13** of the modified manuscript:

"The nitrogen (N_2) adsorption–desorption isotherms of CNT/GP micro-conduit electrodes with different GP growth times are provided in Supplementary Fig. 15, and the measured BET surface area (with GP growth time of 18 min) is 30.6 m^2 g^{-1}, which is nearly 2 times greater than that of the bare CNT micro-conduit structure (15.5 m^2 g^{-1}) (see the Supplementary Fig. 15), 100 times greater than that of CC/GPs (0.32 m^2 g^{-1}) and 300 times greater than that of pure CC (0.11 m^2 g^{-1}), confirming the large specific surface area of CNT/GP micro-conduit electrodes."

Moreover, the nitrogen (N_2) adsorption–desorption isotherms of bare CNT micro-conduit and CNT/GP micro-conduit electrodes with GP growth times of 10 min, 18 min and 45 min have been added on **page 16** of the modified Supplementary Figure 15.

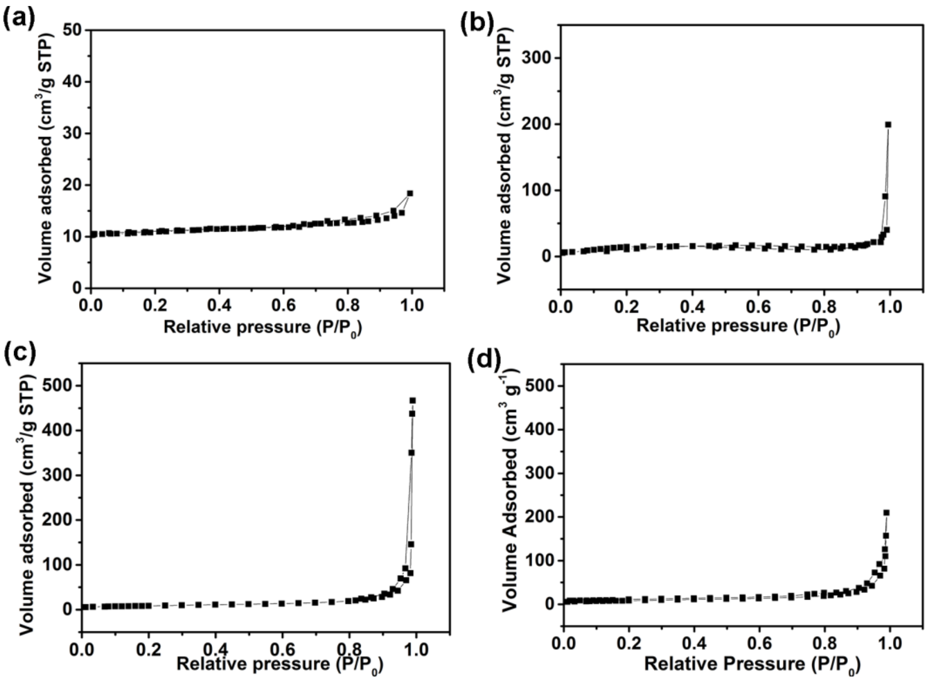

Supplementary Figure 15 | Nitrogen (N_2) adsorption-desorption isotherm curves of (a) bare CNT micro-conduit electrode, (b) CNT/GP micro-conduit electrode with GP growth time of 10 min, (c) CNT/GP micro-conduit electrode with GP growth time of 18 min, (d) CNT/GP micro-conduit electrode with GP growth time of 45 min.

Comment 4. To calculate the gravimetric specific capacitance (500 F/g) of supercapacitors based on CNT/graphene micro-conduits and their composites, the mass loading of CNT, graphene and other pseudocapacitive materials should be evaluated convincingly. However, the explanation on calculation of CNT/graphene loading is not acceptable in the response to the comments, because very little deviation may result the specific capacitance overevaluated.

Authors' Response: We appreciate this concern and agree with the suggestion that the mass loading of CNT, graphene and other pseudocapacitive materials should be reported convincingly to calculate the gravimetric specific capacitance. In our work, the mass loading of active materials (e.g. CNT, GPs) was evaluated by measuring the weight difference of substrate before and after the MPCVD process using a microbalance with an accuracy of 1 μg. Mass measurements were carried out at least three times with different samples, and the averaged value was used as the active mass of CNT/GPs. The mass difference of a substrate before and after MPCVD processing has been generally adopted in prior work to evaluate the mass loading of active materials as supercapacitor electrodes[1, 2, 3, 4]. Thus, in order to compare with the state-of-the-art carbon nanostructure-based electrodes (see

Supplementary Table 1), we should use the same calculation method (mass difference) while ensuring accurate and precise measurements to evaluate the gravimetric specific capacitance of CNT/GP micro-conduit electrodes. Moreover, to comprehensively evaluate the electrochemical performance of the electrodes, we also provide the specific capacitance based on the total mass of CNT/GP/CC (including the mass of current collector, carbon cloth), which is 73.6 F g^{-1}.

Furthermore, to evaluate the electrochemical performance of the electrodes thoroughly, we also provided areal capacitances of the CNT/GP/CC electrodes because areal capacitances (or energy and power densities normalized by area) of electrodes are often more meaningful and suitable for supercapacitors in practical applications[5, 6, 7]. The areal capacitances of the state-of-the-art carbon electrodes are generally low (fall in the range of 0.001 to 0.1 F cm^{-2}), difficult to meet requirements for high-performance supercapacitors in practical applications. In our work, CNT/GP micro-conduits on CC as all-carbon electrodes exhibit ultrahigh areal-capacitance (2.35 F cm^{-2}) and high gravimetric-specific-capacitance (500 F g^{-1}). Our intention was and is to be as accurate and comprehensive as possible; as such readers of our paper can choose capacitances based on mass of active materials, mass of total mass of electrodes, or area of the electrodes in this work for comparison with the performance of the state-of-the-art electrodes reported on a relevant basis.

Changes to Manuscript: According to this comment, we have made the following changes on **page 32** of the modified Supplementary Methods:

"The mass loading of active materials (e.g., CNT, GPs) was evaluated by measuring the weight difference of a substrate before and after the MPCVD process using a microbalance with an accuracy of 1 μg. Mass measurements were carried out at least three times with different samples, and the averaged value was used as the active mass of CNT/GPs. The specific capacitance based on total mass of CNT/GP/CC is calculated to be 73.6 F g^{-1} at 1 mA cm^{-2}."

Comment 5. "…the bulk electrolyte resistance Re of CNT and CNT/GP micro-conduit electrodes is measured…" in Page 9 and other places in the manuscript is not correct. The X-axis intercept of Nyquist Plot represents the series resistance derived from electrode, electrolyte and the interface between electrode and electrolyte.

Authors' Response: We greatly appreciate this comment. The X-axis intercept of Nyquist Plot indeed represents the series resistance derived from electrode, electrolyte and the interface between electrode and electrolyte. We have made relevant modifications throughout the modified manuscript.

Changes to Manuscript: According to this comment, we have made relevant modifications throughout the revised manuscript that "bulk electrolyte resistance R_e" has been correctly modified to "the series resistance R_e (derived from electrode, electrolyte and the interface between electrode and electrolyte)".

Comment 6. Did the CNT/GP micro-conduit well maintain their 3D micro-conduit structure after loading PANI and Ni-Co hydroxide? It cannot be observed from Figure S16 and S17.

Authors' Response: We appreciate this comment. SEM images at low magnification of 3D micro-conduit structure after loading PANI and Ni-Co hydroxide have been additionally provided in modified Supplementary Figure S16 and S17 to demonstrate the outstanding mechanical robustness of the CNT/GP micro-conduit structures. As shown in Supplementary Figure S16a-b and S17a-b, after loading of PANI and Ni-Co hydroxide, the 3D micro-conduit structure is well preserved, further indicating the outstanding mechanical robustness of such CNT/GP micro-conduit structures.

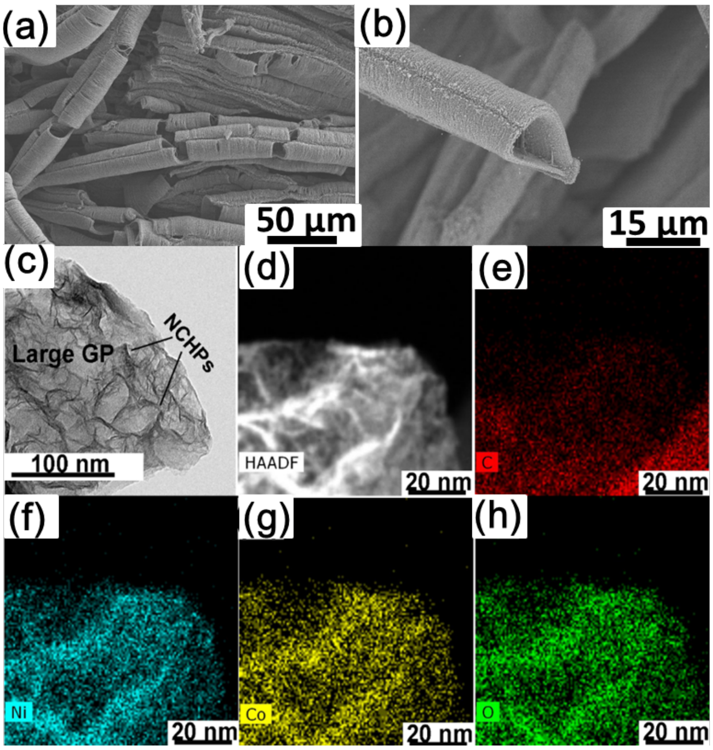

Supplementary Figure 16 | Microstructure of Ni-Co hydroxide petals (NCHPs) electro-deposited on CNT/GP micro-conduit: (a) SEM image of CNT/GP/NCHP micro-conduits at low magnification. (b) SEM image of typical "heart-shape" morphology after NCHP deposition. (c) TEM image of a GP covered with NCHPs. (d) High-angle annular dark field (HAADF) scanning TEM image of a magnified part of a GP covered with many small NCHPs. (e-h) Elemental mapping showing

uniform spatial distribution of elements in (d): (e) to (h) correspond to C, Ni, Co and O element maps, respectively.

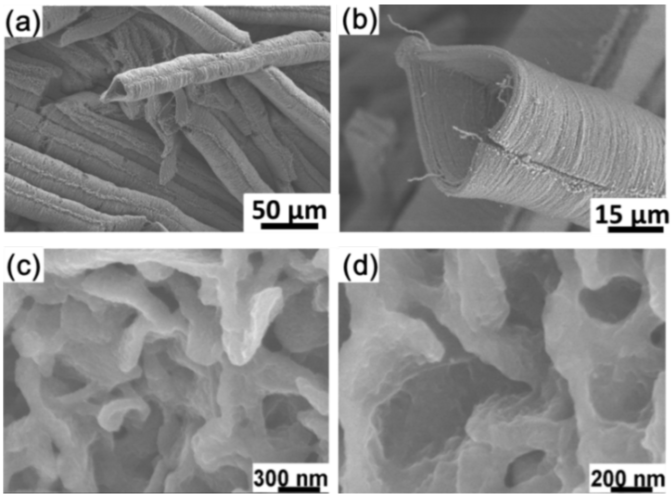

Supplementary Figure 17 | SEM images of CNT/GP/PANI micro-conduits: (a) At low magnification. (b) A typical micro-conduit in a heart-shape. (c) Uniform PANI coating on GP surface. (d) Rough surface of the electrode after electrodeposition of PANI.

Changes to Manuscript: According to this comment, we have added the SEM images at low magnification of 3D micro-conduit structures after loading PANI and Ni-Co hydroxide in modified Supplementary Figure S16 (**Page 17**) and S17 (**Page 18**) and related statements in the modified Supplementary Information:

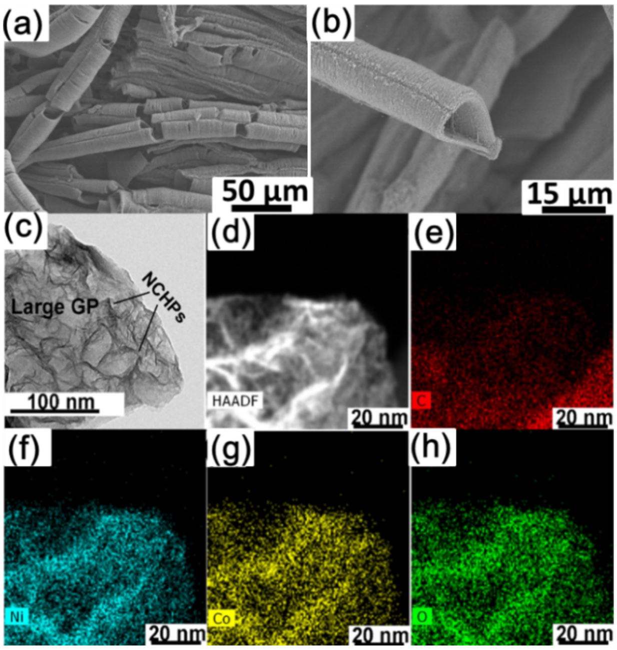

Supplementary Figure 16 | Microstructure of Ni-Co hydroxide petals (NCHPs) electro-deposited on CNT/GP micro-conduit: (a) SEM image of CNT/GP/NCHP

micro-conduits at low magnification. (b) SEM image of typical "heart-shape" morphology after NCHP deposition. (c) TEM image of a GP covered with NCHPs. (d) High-angle annular dark field (HAADF) scanning TEM image of a magnified part of a GP covered with many small NCHPs. (e-h) Elemental mapping showing uniform spatial distribution of elements in (d): (e) to (h) correspond to C, Ni, Co and O element maps, respectively.

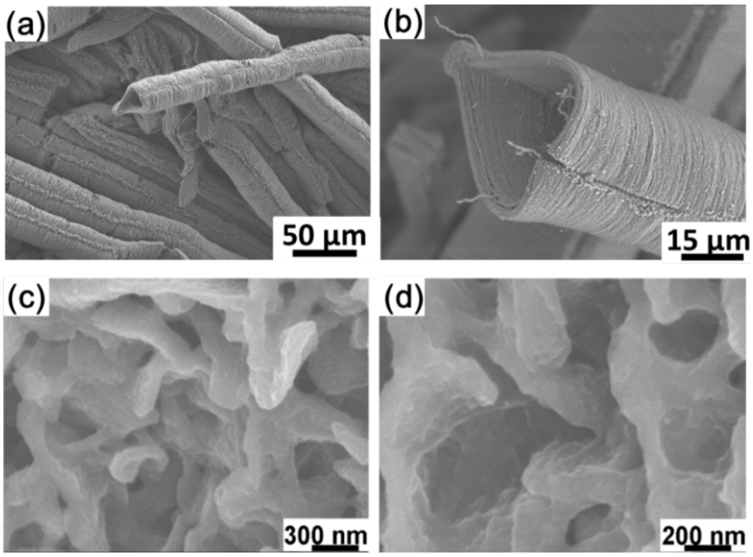

Supplementary Figure 17 | SEM images of CNT/GP/PANI micro-conduits: (a) At low magnification. (b) A typical micro-conduit in a heart-shape. (c) Uniform PANI coating on GP surface. (d) Rough surface of the electrode after electrodeposition of PANI.

References:

1. Wu AM, Feng C C, Huang H, *et al.* Microwave plasma-assisted chemical vapor deposition of porous carbon film as supercapacitive electrodes. *Applied Surface Science* **409**, 261-269 (2017).

2. Ding Q, Li W L, Zhao W L, *et al.* Plasma assisted fabrication of multi-layer graphene/nickel hybrid film as enhanced micro-supercapacitor electrodes. *IOP Conference Series: Materials Science and Engineering* **182**, 012014 (2017).

3. Hsu HC, Wang C H, Nataraj S K, *et al.* Stand-up structure of graphene-like carbon nanowalls on CNT directly grown on polyacrylonitrile-based carbon fiber paper as supercapacitor. *Diamond & Related Materials* **25**, 176-179 (2012).

4. Wei S, Kang WP, Davidson JL, *et al.* Supercapacitive behavior of CVD carbon nanotubes grown on Ti coated Si wafer. *Diamond & Related Materials* **17**, 906-911 (2008).

5. Gogotsi Y, Simon P. Materials science. True performance metrics in electrochemical energy storage. *Science* **334**, 917 (2011).

6. Miller JR. Valuing Reversible Energy Storage. *Science* **335**, 1312-1313 (2012).

7. Stoller MD, Ruoff RS. Best practice methods for determining an electrode material's performance for ultracapacitors. *Energy & Environmental Science* **3**, 1294-1301 (2010).

REVIEWERS' COMMENTS:

Reviewer #4 (Remarks to the Author):

The authors did more relative experiments to support their claim, and well responded the comments from reviewers. Therefore, I think this manuscript could be published in Nature Communications.

Author response to Review No. 4

Bioinspired Leaves-on-branchlet Hybrid Carbon Nanostructure for Supercapacitors

by

Guoping Xiong, Pingge He, Zhipeng Lyu , Tengfei Chen, Boyun Huang, Lei Chen, Timothy S. Fisher

"The authors did more relative experiments to support their claim, and well responded the comments from reviewers. Therefore, I think this manuscript could be published in Nature Communications."

Authors' Response: We thank the reviewer for careful reading and appreciation of our work.
